# Supplementary material for: Exogenous prion-like proteins and their potential to trigger cognitive dysfunction
Source: Mol Syst Biol. 2025 May 27;21(8):1004–29. doi: 10.1038/s44320-025-00114-4 (PMC12322145; doi:10.1038/s44320-025-00114-4)
Supplement: Supplementary file 1 — Appendix [file 44320_2025_114_MOESM1_ESM.pdf]

Appendix

Microbiome-Derived Prion-Like Proteins and Their Potential to Trigger Neurodegeneration

Table of Content

Supplementary Figures

Appendix Figure S1 .... Page 2
Appendix Figure S2 .... Page 3
Appendix Figure S3 .... Page 4
Appendix Figure S4 .... Page 5
Appendix Figure S5 .... Page 6
Appendix Figure S6 .... Page 7
Appendix Figure S7 .... Page 8
Appendix Figure S8 .... Page 9
Appendix Figure S9 .... Page 10
Appendix Figure S10 .... Page 11
Appendix Figure S11 .... Page 12
Appendix Figure S12 .... Page 13
Appendix Figure S13 .... Page 14
Appendix Figure S14 .... Page 15
Appendix Figure S15 .... Page 16
Appendix Figure S16 .... Page 17
Appendix Figure S17 .... Page 18
Appendix Figure S18 .... Page 19
Appendix Figure S19 .... Page 20
Appendix Figure S20 .... Page 21
Appendix Figure S21 .... Page 22
Appendix Figure S22 .... Page 23

Appendix Supplementary Data

Predictions performed on the Sup35p variants .... Page 24
Protein sequences expressed in *S. cerevisiae* .... Page 39
Protein sequences expressed in *E. coli* .... Page 40

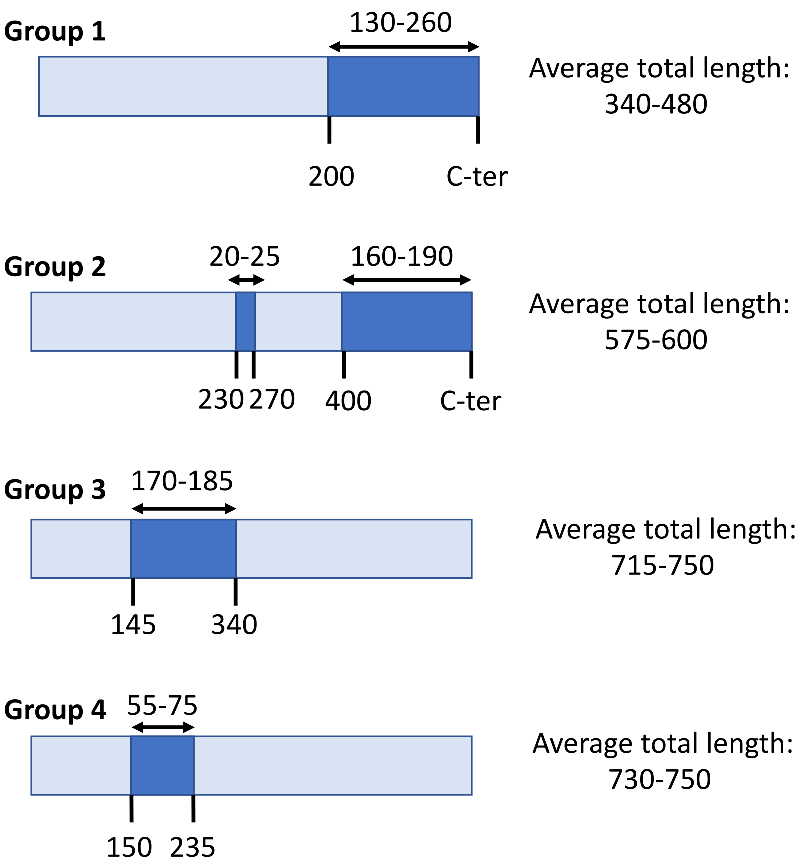

Appendix Figure S1. Common Disordered Regions Identified in Uncharacterized Proteins. The diagram displays four sequences, highlighting the consensus locations identified within the gut microbiome. This classification is based on information sourced from MobiDB Lite (Supplementary Table 1). In Supplementary Table 1, there are 370 uncharacterized sequences, constituting 40% of the dataset. Among these, 214 sequences have been determined to contain disordered regions according to MobiDB Lite. The groups shown in this figure consist of the following numbers of sequences: Group 1, 78 sequences; Group 2, 39 sequences; Group 3, 35 sequences; Group 4, 23 sequences.

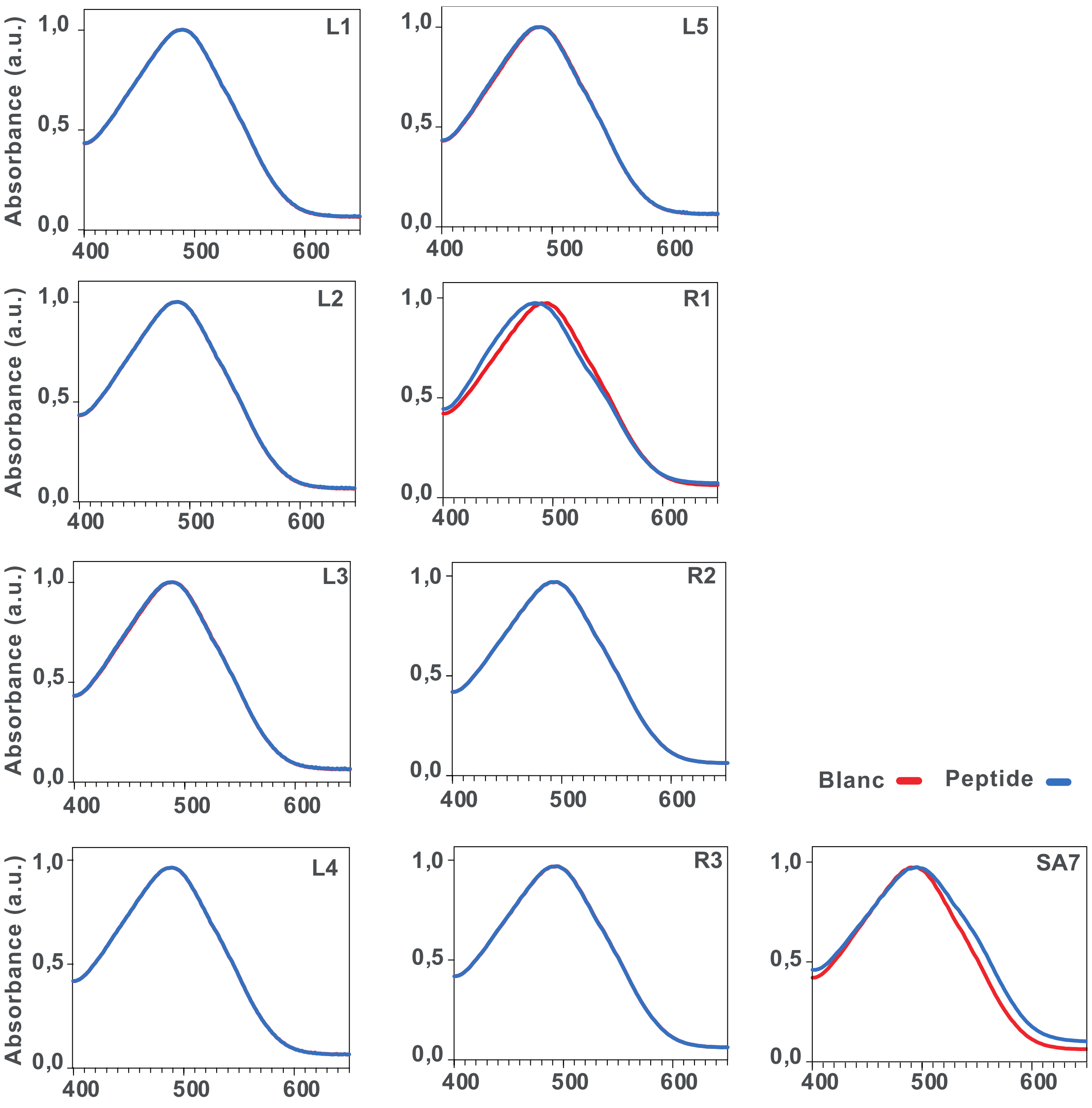

Appendix Figure S2. Congo Red binding analysis of the aggregates formed by the ten selected amyloid cores. Red indicates Congo Red absorbance without aggregates, while blue represents absorbance with aggregates.

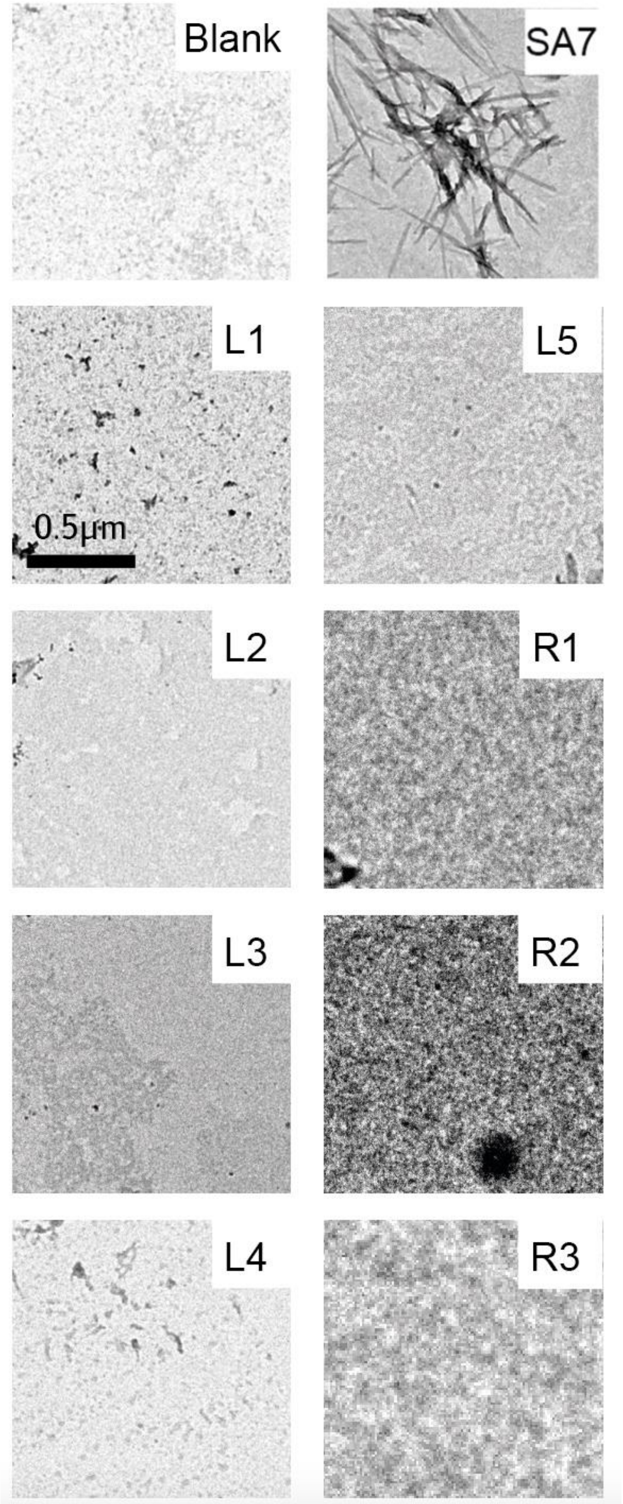

Appendix Figure S3. TEM images showing the samples of low aggregation-prone peptides.

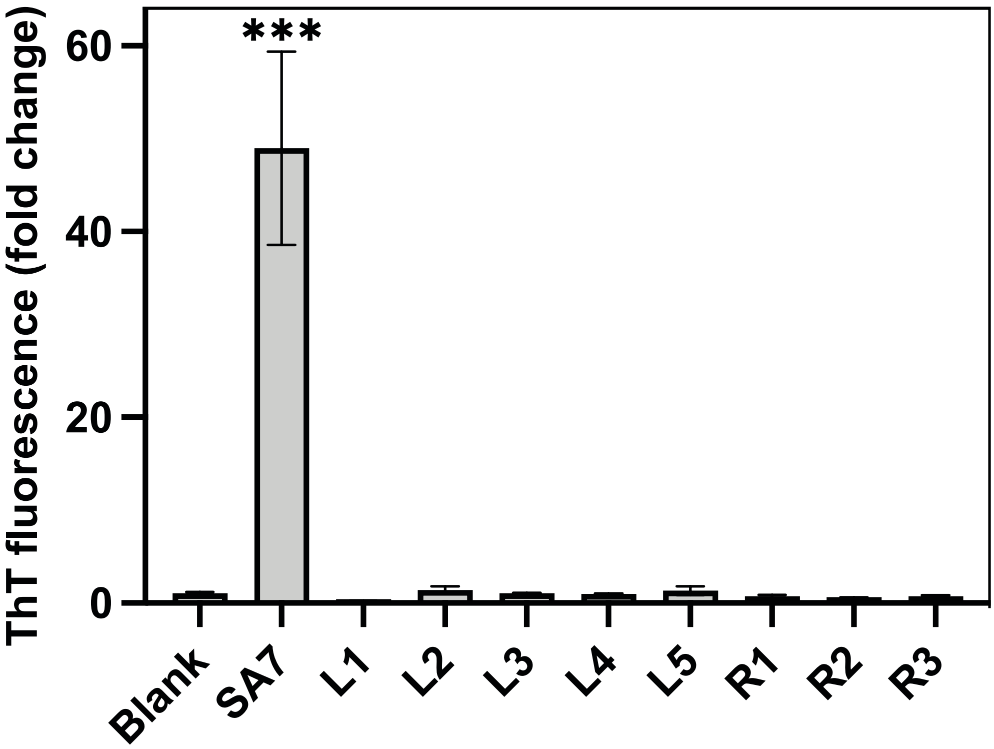

Appendix Figure S4. Thioflavin-T binding of the low aggregation-prone control peptides. Thioflavin-T fluorescence increase, fold change with respect to the blank (just buffer and ThT). The binding of the peptide SA7 was also measured as a positive control (unpaired two tail t-test, N=3).

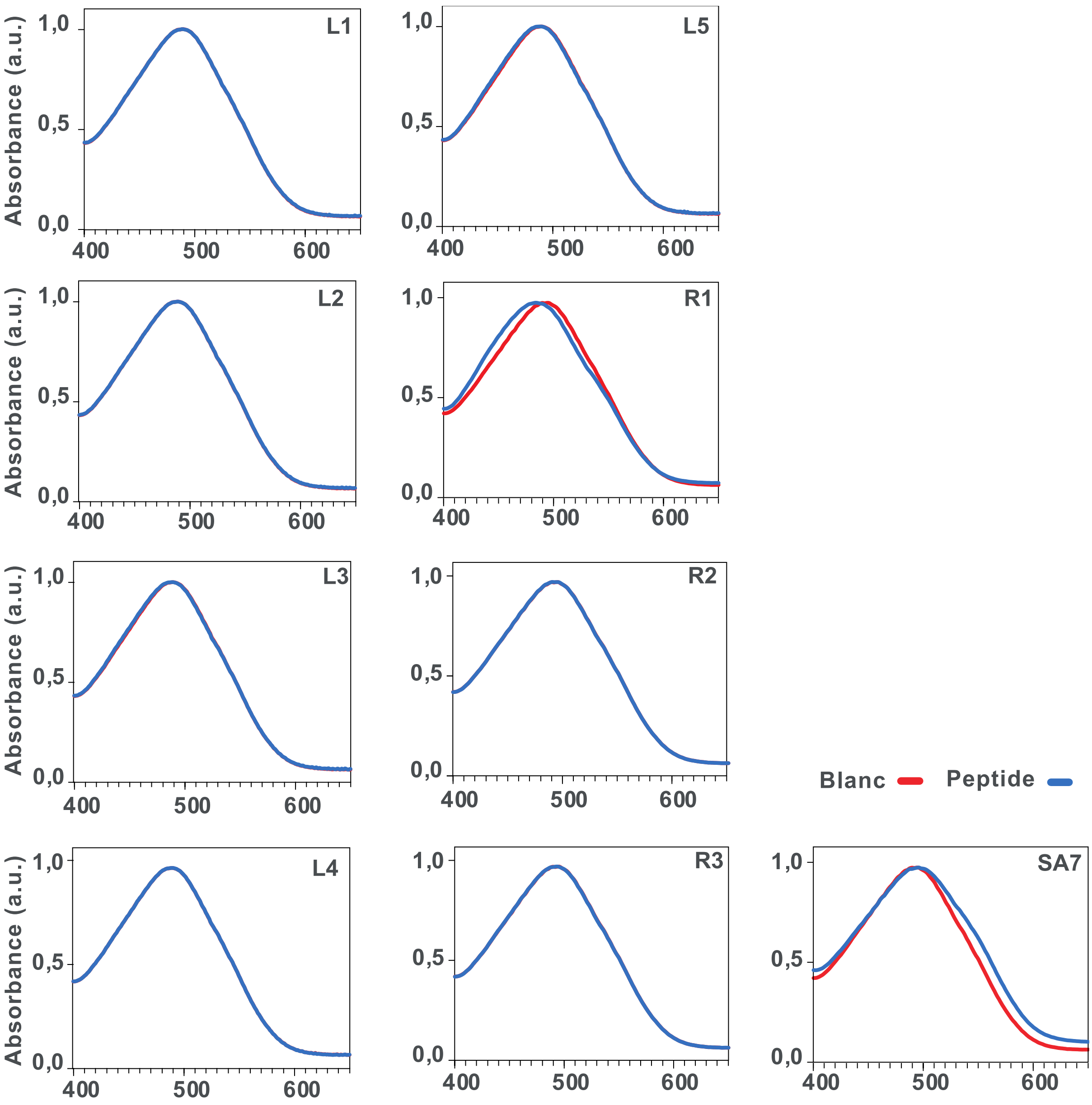

Appendix Figure S5. Congo Red binding analysis of the aggregates formed by the eighth low aggregation-prone sequences. Red indicates Congo Red absorbance without aggregates, while blue represents absorbance with the control peptides.

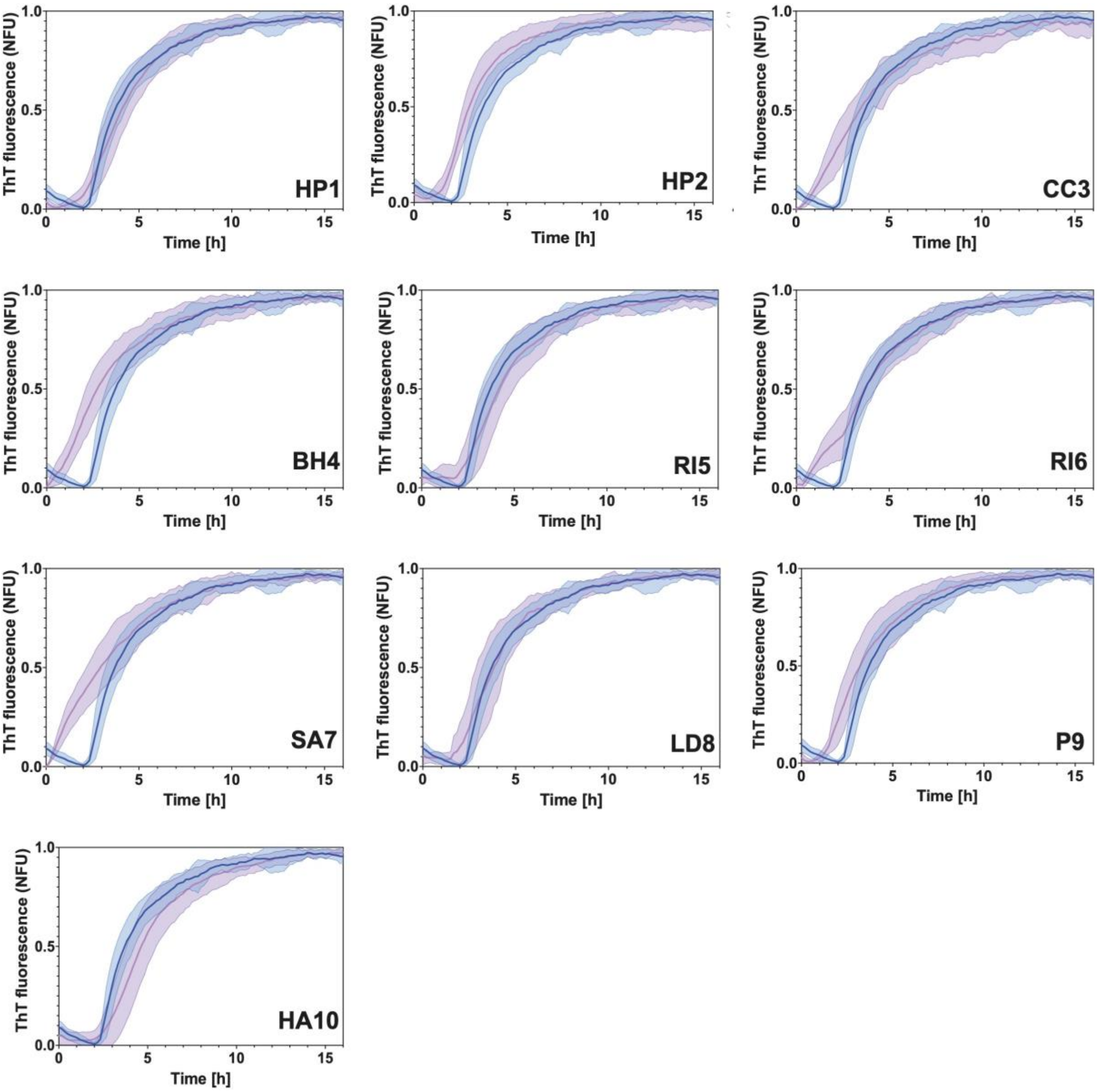

Appendix Figure S6. Aggregation kinetics plots of Aβ40 seeded with ten peptides derived from the gut microbiome. The purple curve represents the aggregation kinetics of Aβ40 without any seeding, whereas the blue curve represents the aggregation kinetics in the presence of pre-aggregated peptides. The shaded regions show the standard error of the mean, which has been calculated from four independent replicates, with each replicate comprising three repeated samples.

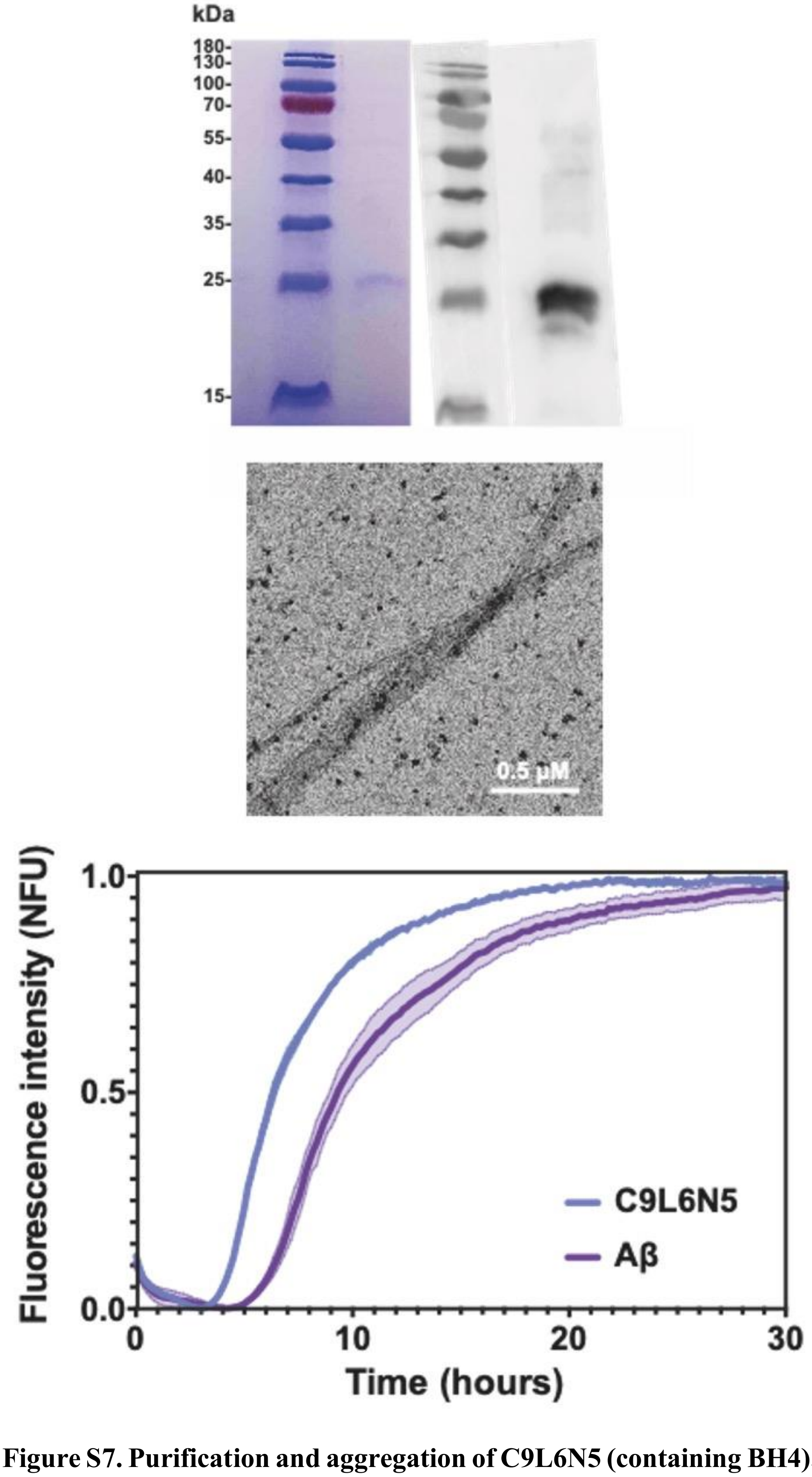

Appendix Figure S7. Purification and aggregation of C9L6N5 (containing BH4). Up, SDS-PAGE gel and western blotting (anti-His) showing one protein band after C9L6N5 purification. Middle, TEM image showing fibrillar aggregates of C9L6N5. Bottom, Aβ40 aggregation kinetics not seeded (purple) and seeded (blue) with aggregates of C9L6N5.

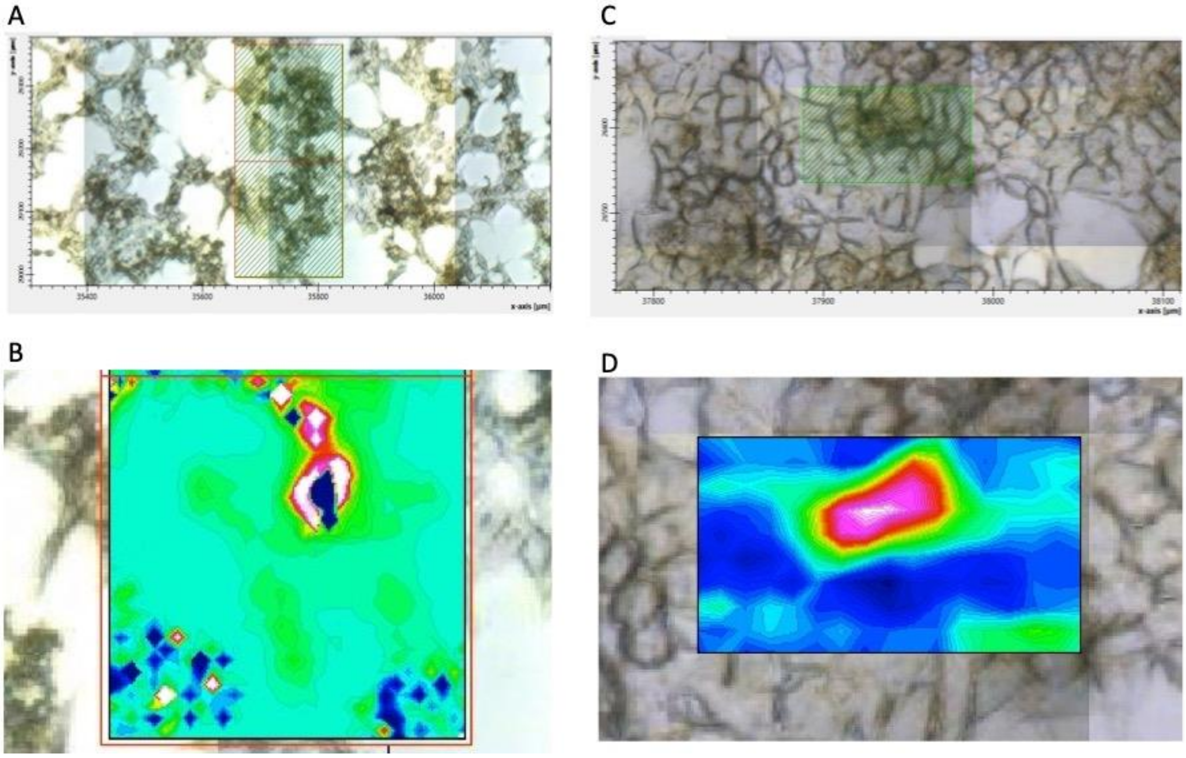

Appendix Figure S8. µFTIR maps of cells treated with aggregated peptides derived from the microbiota. The upper panels show microscope images of cells incubated with the peptides (A) HP2 and (C) BH4. The lower panels illustrate the beta/alpha ratio (1740/2921 cm-1), utilized for detecting the presence of amyloid fibrils formed by the peptides (B) HP2 and (D) BH4. The images indicate the distribution of preformed aggregates throughout the samples. Amyloid deposits were identified in all samples, confirming the persistence of the preaggregated peptide's under cell culture conditions.

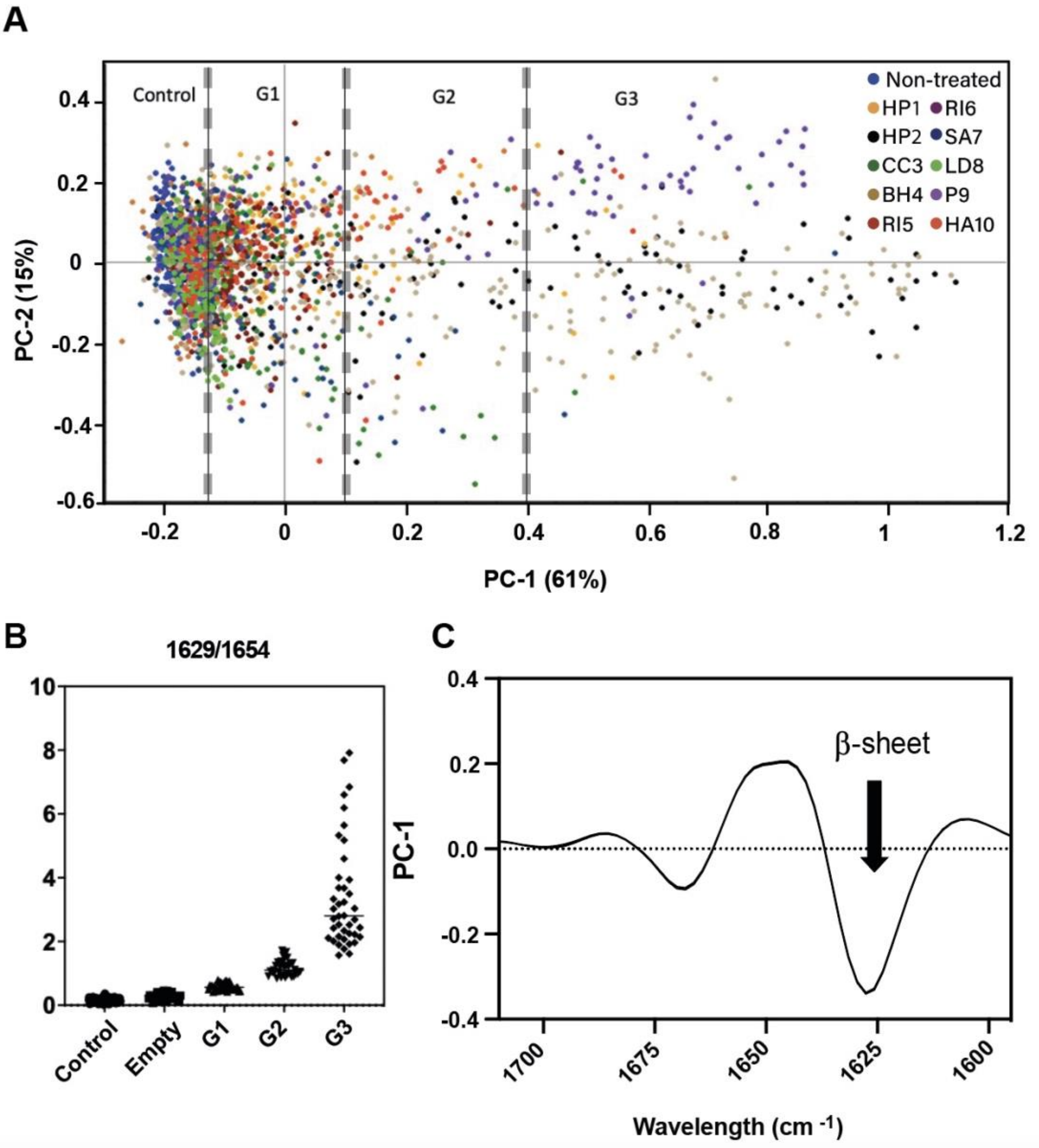

Appendix Figure S9. Results of µFTIR analysis showing that the absorbance of β-sheet differentiates between cells incubated with and without aggregates. Neuron differentiate SH-SY5Y cells were incubated with and without the aggregates of the amyloid cores selected. Then for each sample, the presence and structure of amyloid-like aggregates were measured with µFTIR. Two negative controls were considered: cells growth without aggregates (Control) and the zones where no aggregate was visualized under the microscope (Empty). A) Principal component analysis of the signal at the amide I region. The measurements were separated into three groups (grey dashed line) based on the PC-1, which (as seen in panel C) is principally supported by the β-sheet signal. In this line, the groups G1, G2 and G3 arrange the measurements from less to more β-sheet signal. B) Distribution of the amyloid aggregation ratio (1629/1654) for the three groups derived from PCA analysis (Control, N=1181; Empty, N=139, G1, N=465; G2, N=185; G3, N=220). This result supports that the group division, at the PCA, separates progressively increasing aggregate signals. C) Summary µFTIR spectra of PC-1, representing 61% of the variances between the samples. The peak from 1635 cm-1 to 1625 cm-1 corresponds to β-sheet signal, indicating that the difference between the groups is linked to amyloid fibrils. Amyloid deposits (G1, G2, G3) were identified in all samples, confirming the persistence of aggregated peptides under cell culture conditions.

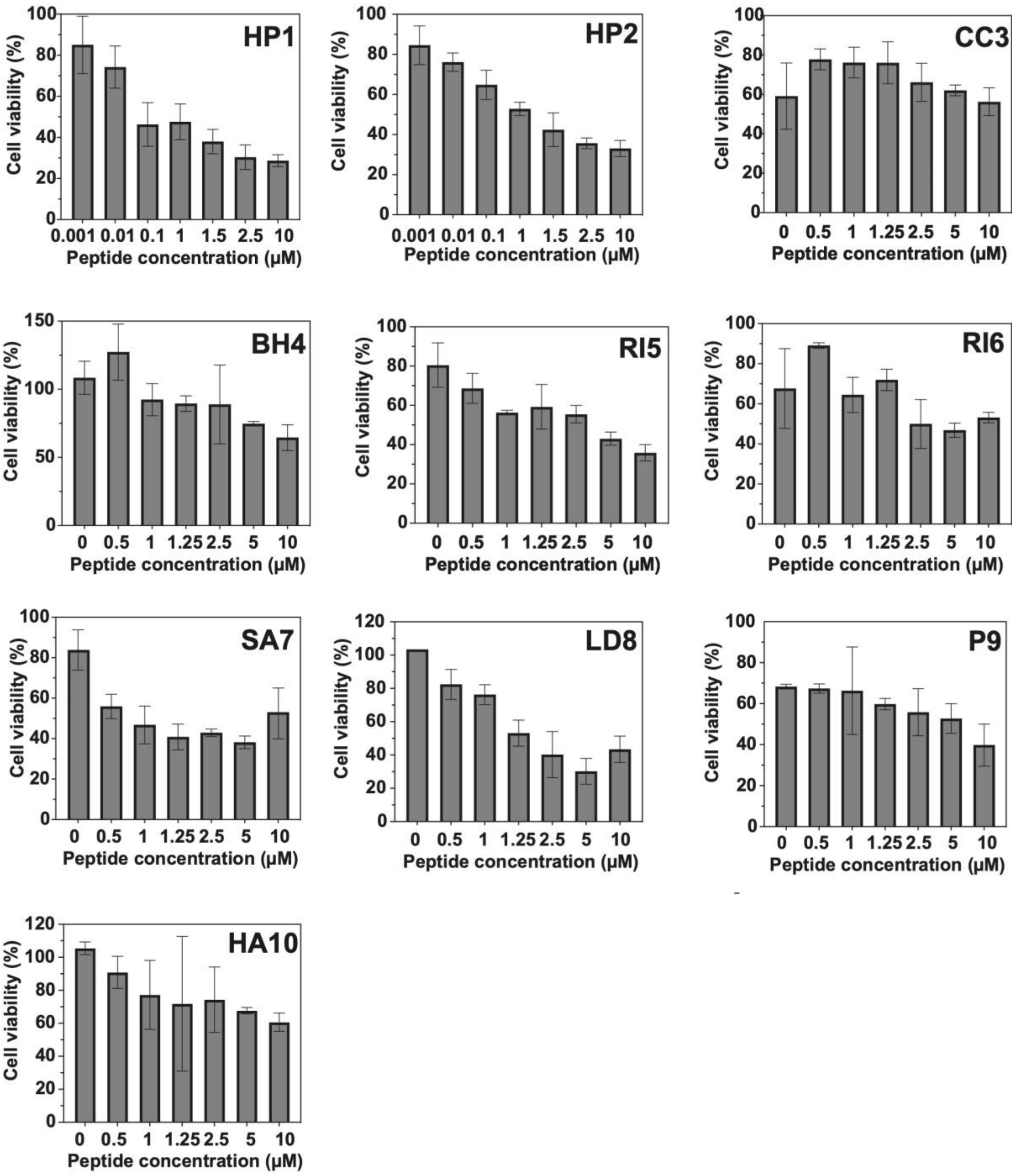

Appendix Figure S10. Cytotoxicity of amyloid-forming cores derived from the gut microbiome. Cell viability of SH-SY5Y cells after 24 hours of exposure to various peptides (N=3). Due to their high toxicity, C1 and C2 have a wider concentration range. The error bars represent the standard error of the mean (SEM).

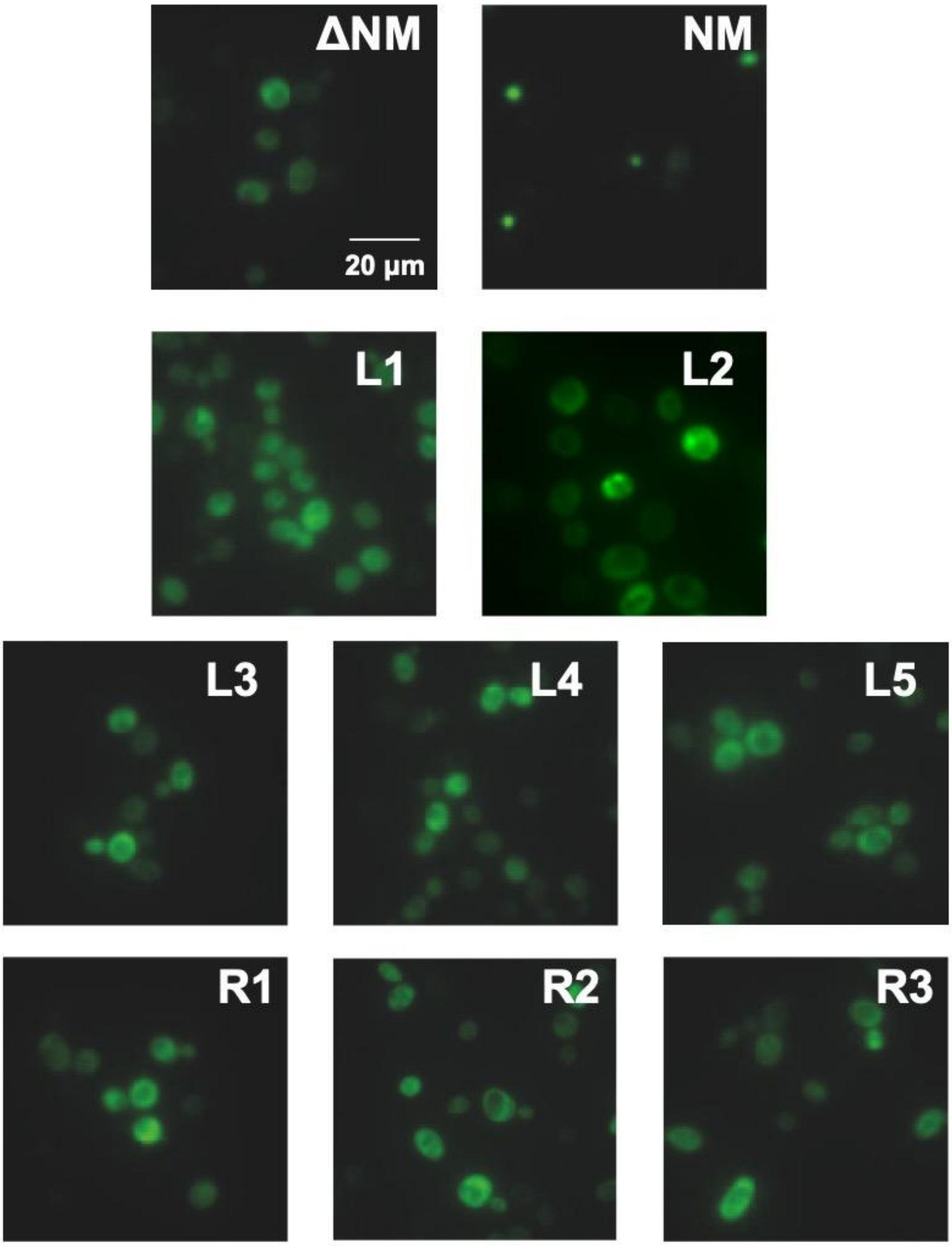

Appendix Figure S11. Yeast expressing low aggregation-prone variants of Sup35-GFP. Fluorescent images showing the location of the Sup35-GFP variants.

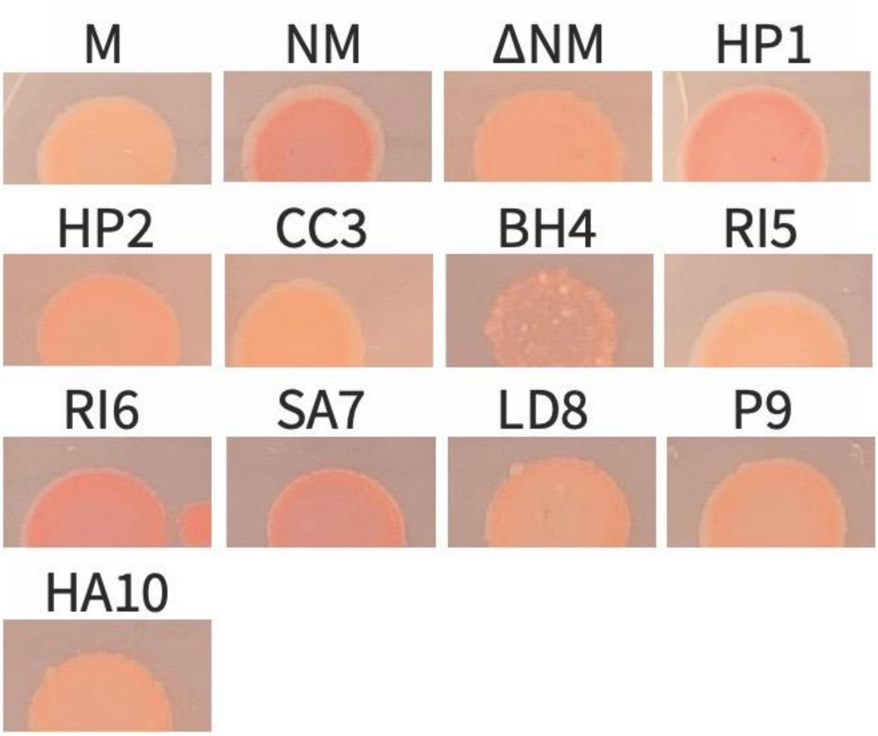

Appendix Figure S12. E. coli C-DAG colonies on Congo Red-containing plates. The images show the colonies of the different *E. coli* expressing the Sup35 chimeras grown on congo-red containing plates (Methods).

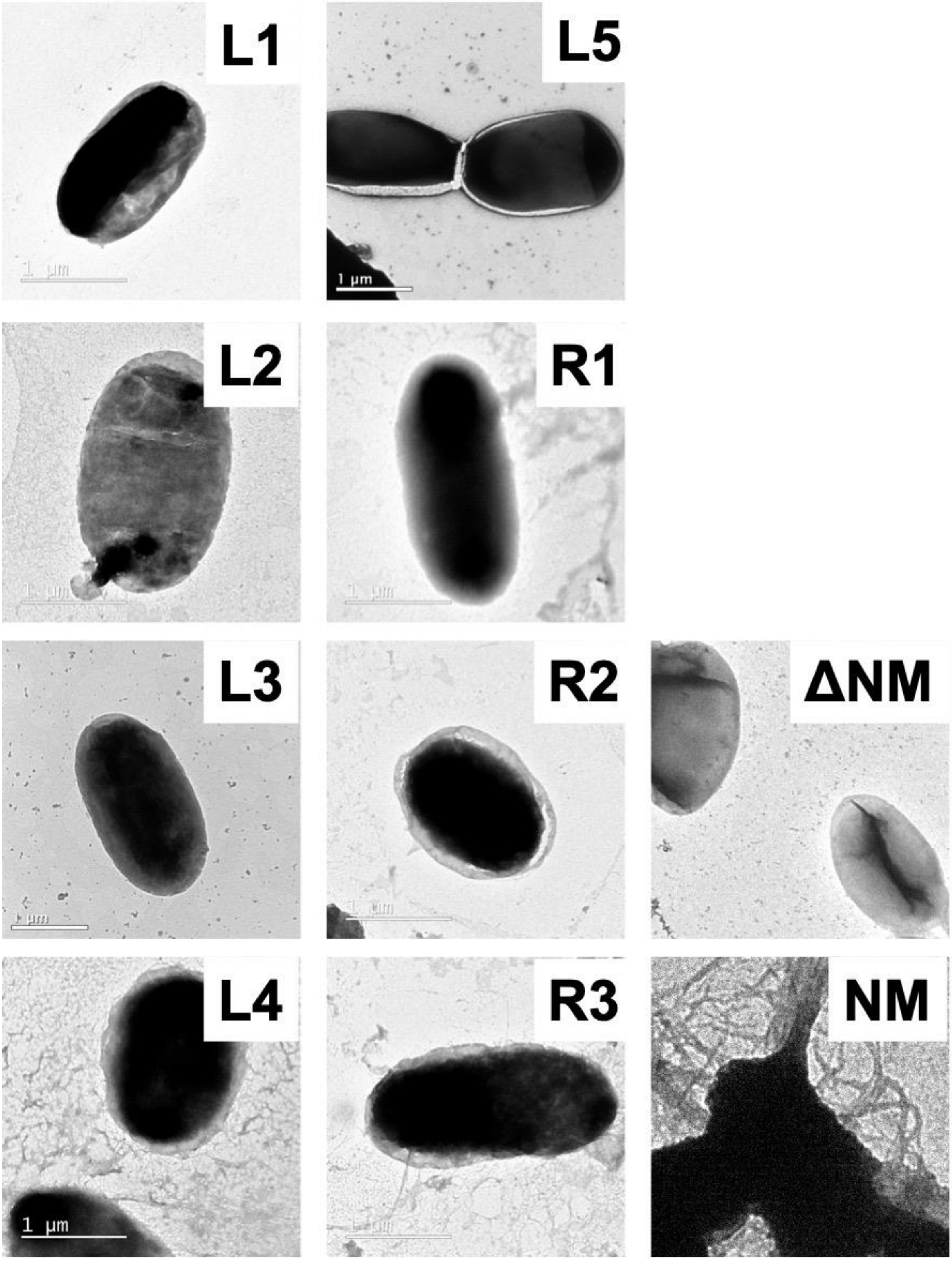

Appendix Figure S13. TEM images of the *E. coli* C-DAG system expressing low aggregation prone Sup35 variants.

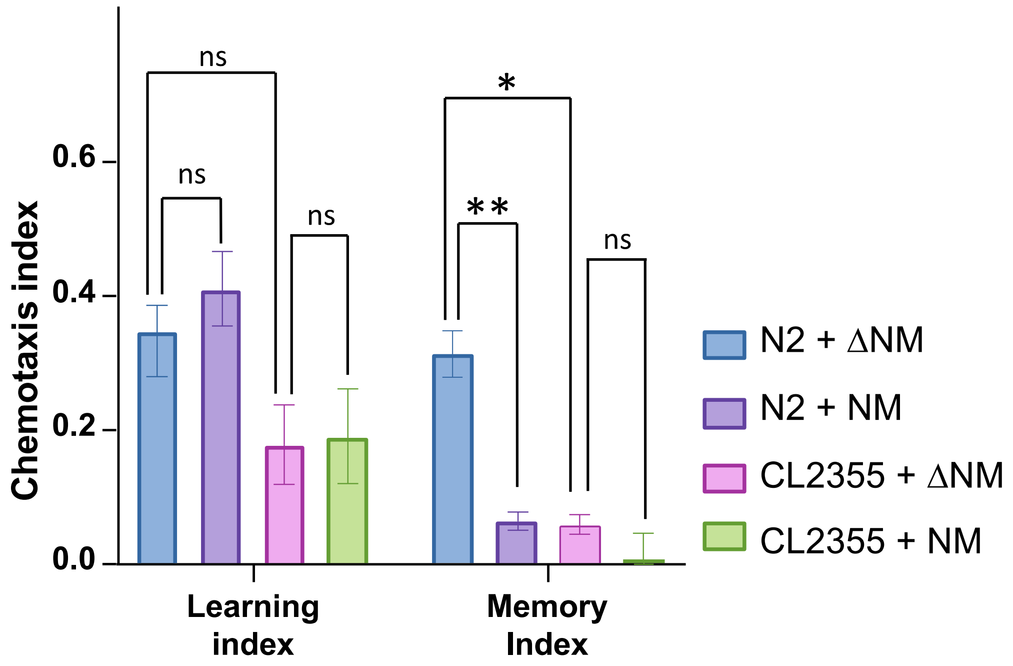

Appendix Figure S14. STAM comparison between N2 (wildtype) and CL2355 (Alzheimer's model). Chemotaxis index for learning and memory assays measured for worms fed with ΔSup35 and Sup35NM (One-way ANOVA, N=3, *$p < 0.05$, **$p < 0.01$, ns = not significant).

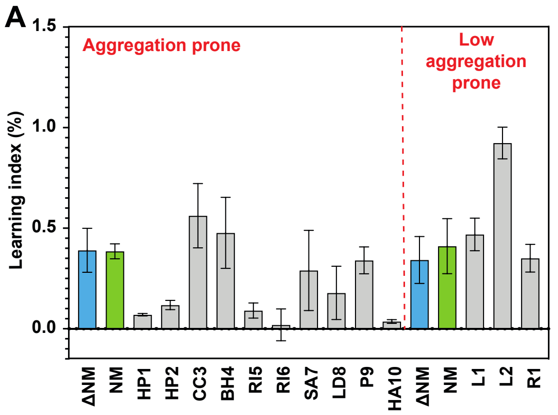

B

Learning	q value	Significantly
NM	0,9421	No
HP1	0,0135	Lower
HP2	0,0071	Lower
CC3	0,1995	No
BH4	0,5103	No
RI5	0,0109	Lower
RI6	0,0089	Lower
SA7	0,4870	No
LD8	0,0991	No
P9	0,5356	No
HA10	0,0050	Lower
NM	0,5450	No
L1	0,3733	No
L2	0,0024	Higher
R1	0,6238	No

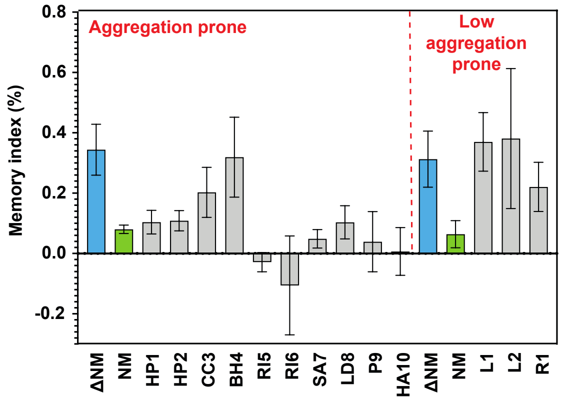

Memory	q value	Significantly
NM	0,0059	Lower
HP1	0,0110	Lower
HP2	0,0109	Lower
CC3	0,1075	No
BH4	0,7984	No
RI5	0,0020	Lower
RI6	0,0134	Lower
SA7	0,0047	Lower
LD8	0,0144	Lower
P9	0,0156	Lower
HA10	0,0072	Lower
NM	0,0140	Lower
L1	0,5021	No
L2	0,6608	No
R1	0,2670	No

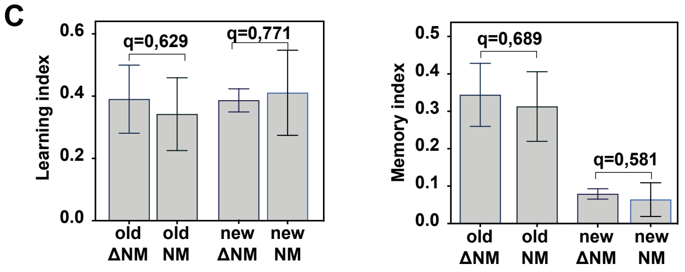

Appendix Figure S15. Low aggregation-prone STAM comparison. A) The plots show the learning (top) and memory (bottom) indices for all the samples analyzed in this study. The vertical dashed line separates two different sets of experiments: on the left, the assays performed with aggregation-prone Sup35 chimeras, and on the right, those conducted with the low-aggregation-prone variants. Samples analyzed in both experiments (internal controls) are highlighted in blue and green. B) Tables showing that the significances against their corresponding ΔNM controls, obtained when including all samples, are similar to those reported in the main article (Figure 6, which only included the aggregation-prone variants). The analysis was performed using multiple unpaired two-tailed t-tests, corrected for multiple comparisons using the Benjamini–Hochberg method (N = 3 biological replicates of 300–500 worms each, FDR set at 5%). C) Comparison between the same samples across the two repeated assays (ΔNM and NM). The analysis was performed using multiple unpaired two-tailed t-tests, corrected for multiple comparisons using the Benjamini–Hochberg method (N = 3 biological replicates of 300–500 worms each, FDR set at 5%). The learning and memory indices obtained for both samples (ΔNM and NM) in the repeated assays showed no significant differences.

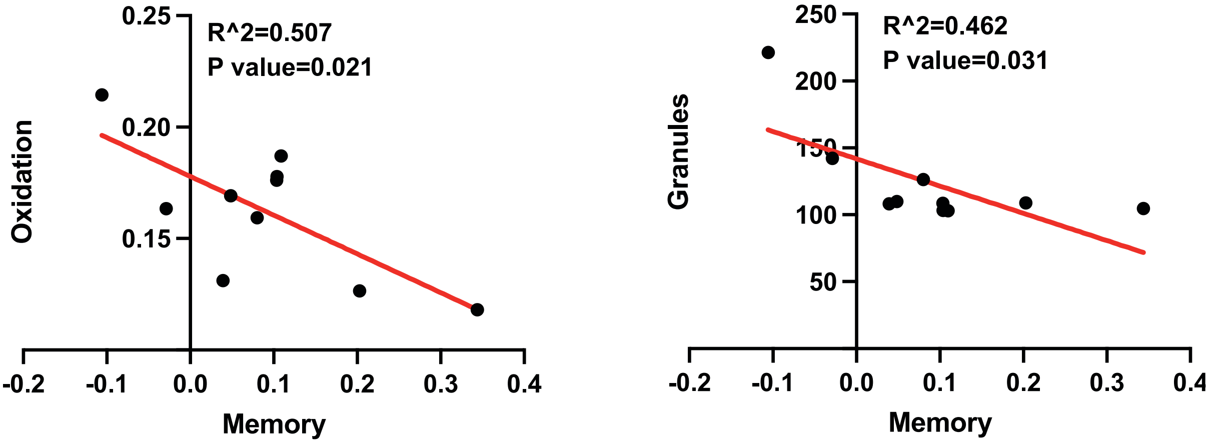

Appendix Figure S16. Scatter plots showing how lipid oxidation and number of granules correlate with the associative memory in *C. elegans*. The red line indicates the linear regression for each dataset (derived from Figures 6D and 7C). BH4 and HA10 were excluded from the analysis as they presented especially high or low values in oxidation and the number of granules, respectively. Statistical significance was assessed using Pearson correlation analysis.

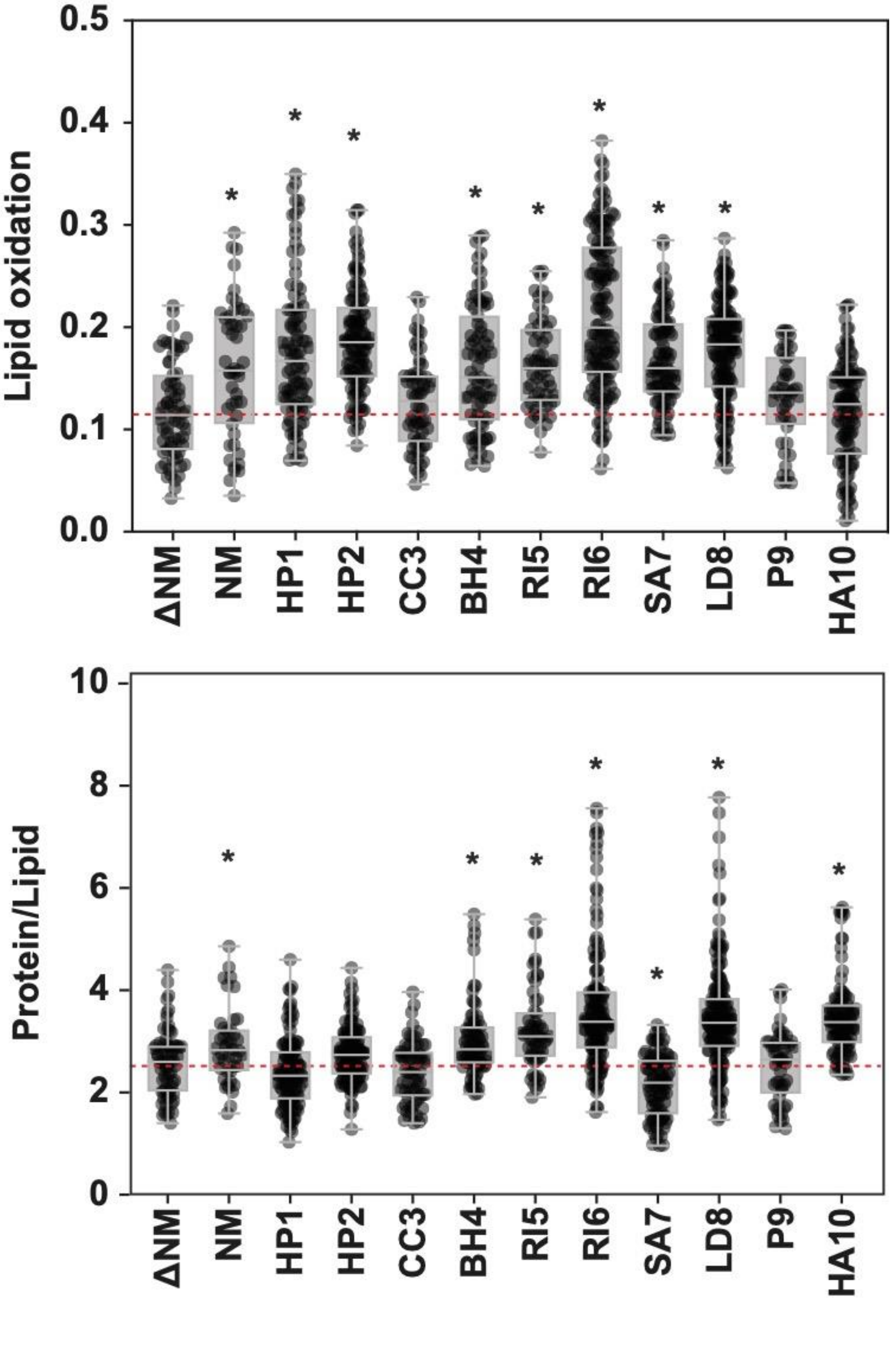

Appendix Figure S17. IR measurements of the whole body of *C. elegans* strains fed with the Sup35 chimeras. Up, distribution of the lipid peroxidation signal (1740 cm^{-1}/(2960 cm^{-1} – 2919 cm^{-1})). Down, proportion protein/lipid signal (1654 cm^{-1}/(2960 cm^{-1} – 2919 cm^{-1})). Each box represents the measurements taken within at least 10 worms. ΔNMdelta N=67; NM N=42; C1 N=110; C2 N=120; C3 N=67; C4 N=82; C5 N=58; C6 1 N=35; C7 N=94; C8 N=159; C9 N=49; C10 N=97. The box plots bars show the minimum and maximum values, and the box lines show the median and the interquartile range. Significance was measured using an unpaired t-test (*p < 0.05).

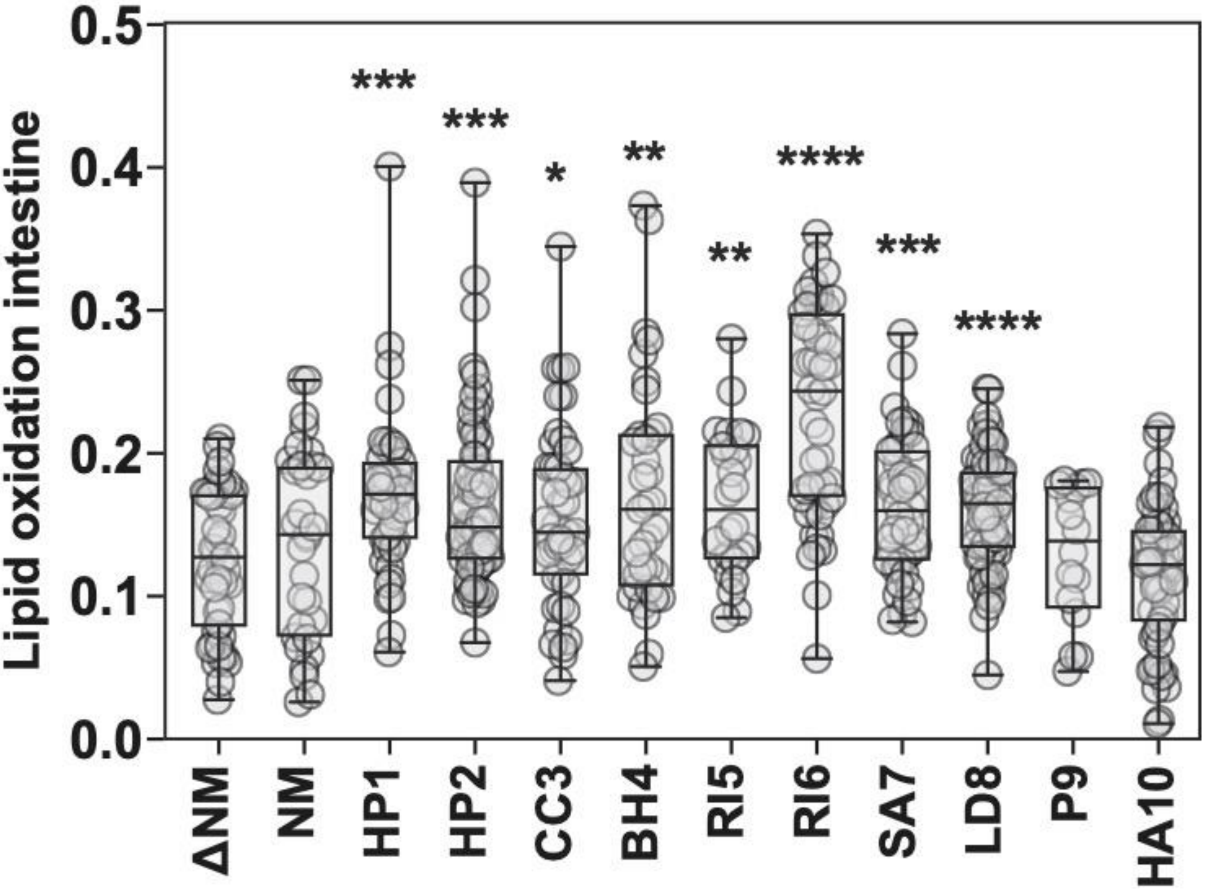

Appendix Figure S18. IR measurements focused on the intestine of *C. elegans* strains fed with the Sup35 chimeras. Distribution of the lipid peroxidation signal (1740 cm^{-1}/(2960 cm^{-1} – 2919 cm^{-1})). Each box represents the measurements taken within the intestine of a minimum of 10 worms per sample and a minimum of 25 points per sample. The box plots bars show the minimum and maximum values, and the box lines show the median and the interquartile range. Significance was measured using an unpaired t-test (*$p < 0.05$, **$p < 0.01$, ***$p < 0.001$, ****$p < 0.0001$).

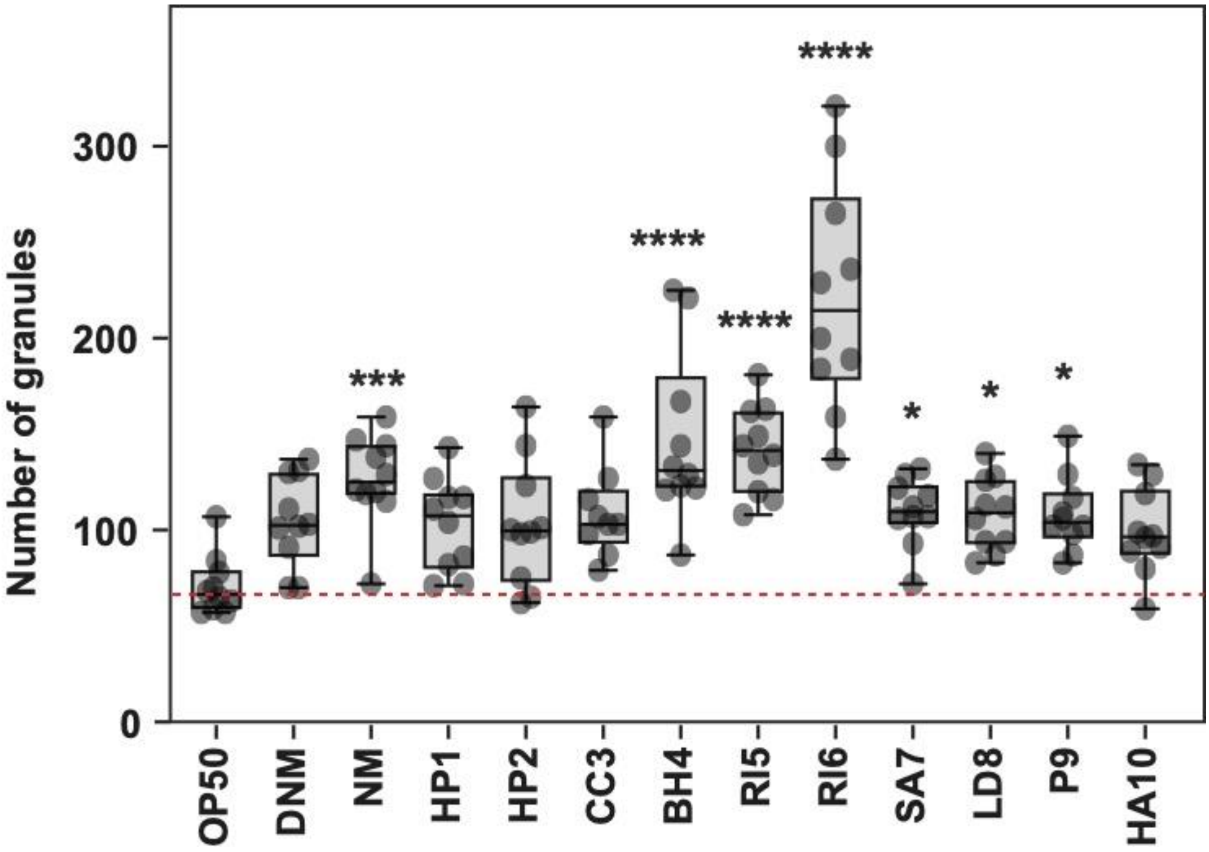

Appendix Figure S19. *C. elegans* gut granules counting. Number of gut granules in the two first intestinal rings of *C. elegans* N2 fed with *E. coli* OP50 and *E. coli* expressing various Sup35NM variants. OP50 *E. coli* strain is the standard nematode food and a biofilm-defective mutant. Error bars represent the SEM of 8-10 measurements. Each dot contains the information of one worm. Statistical significance was determined using one-way ANOVA (*p < 0.05, **p < 0.01, ***p < 0.001, ****p < 0.0001).

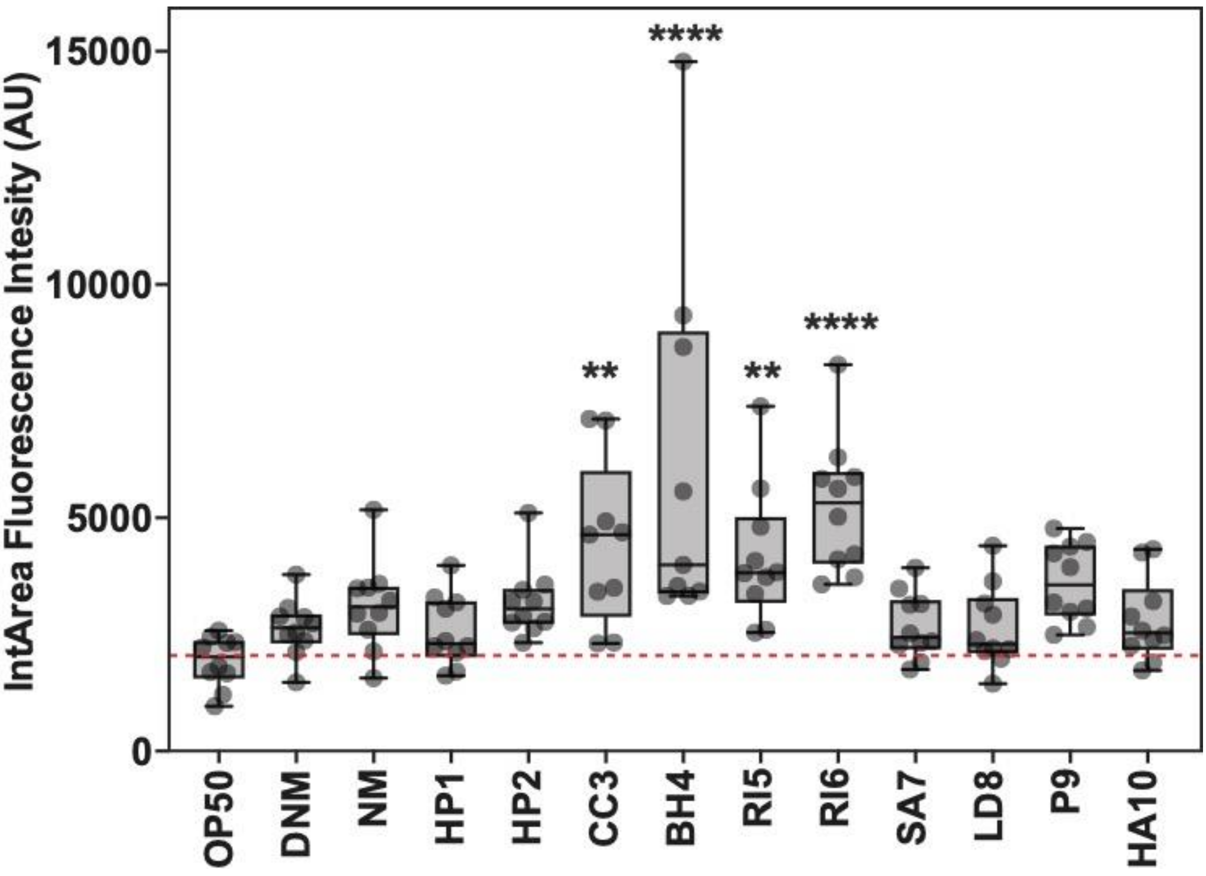

Appendix Figure S20. ***C. elegans*** **gut granules fluorescent intensity.** Plot showing the distribution of the gut granules fluorescent intensity adjusted by area (IntArea, ImageJ) in the *C. elegans* fed with *E. coli* OP50 and *E. coli* expressing different variants of Sup35NM. *E. coli* strain is the standard nematode food and a biofilm-defective mutant. Error bars represent the SEM of 8-10 measurements. Statistical significance was determined using one-way ANOVA (*p < 0.05, **p < 0.01, ***p < 0.001, ****p < 0.0001).

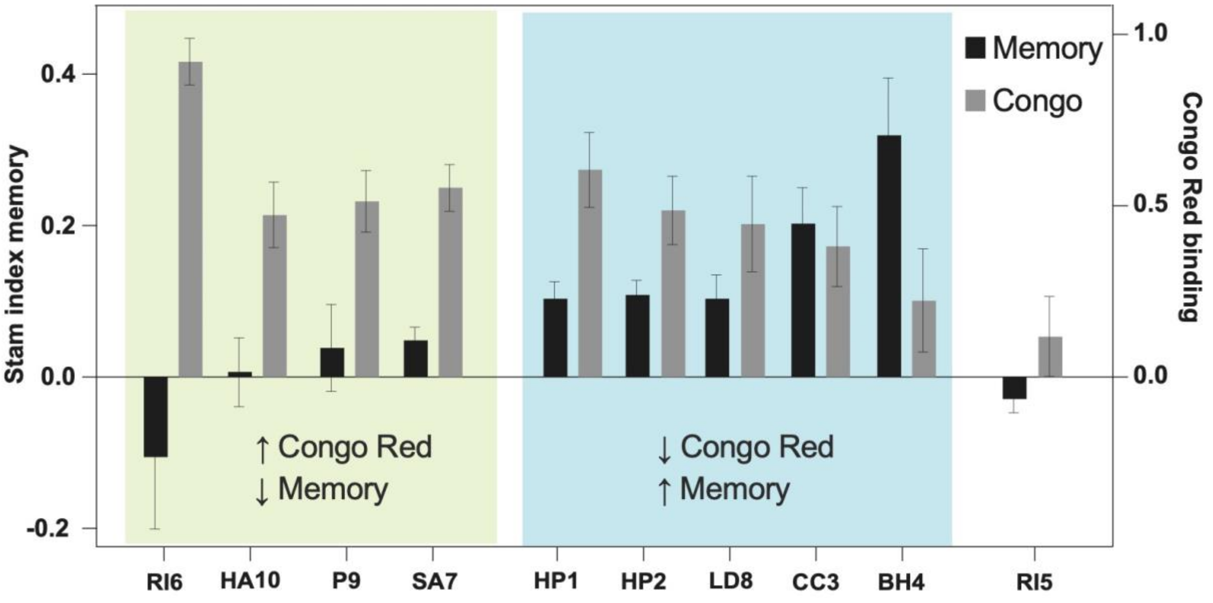

Appendix Figure S21. Memory index and Congo Red binding comparison. Plot comparing the memory index and the congo red binding (from Figure 5B) of *E. coli* expressing different variants of Sup35NM.

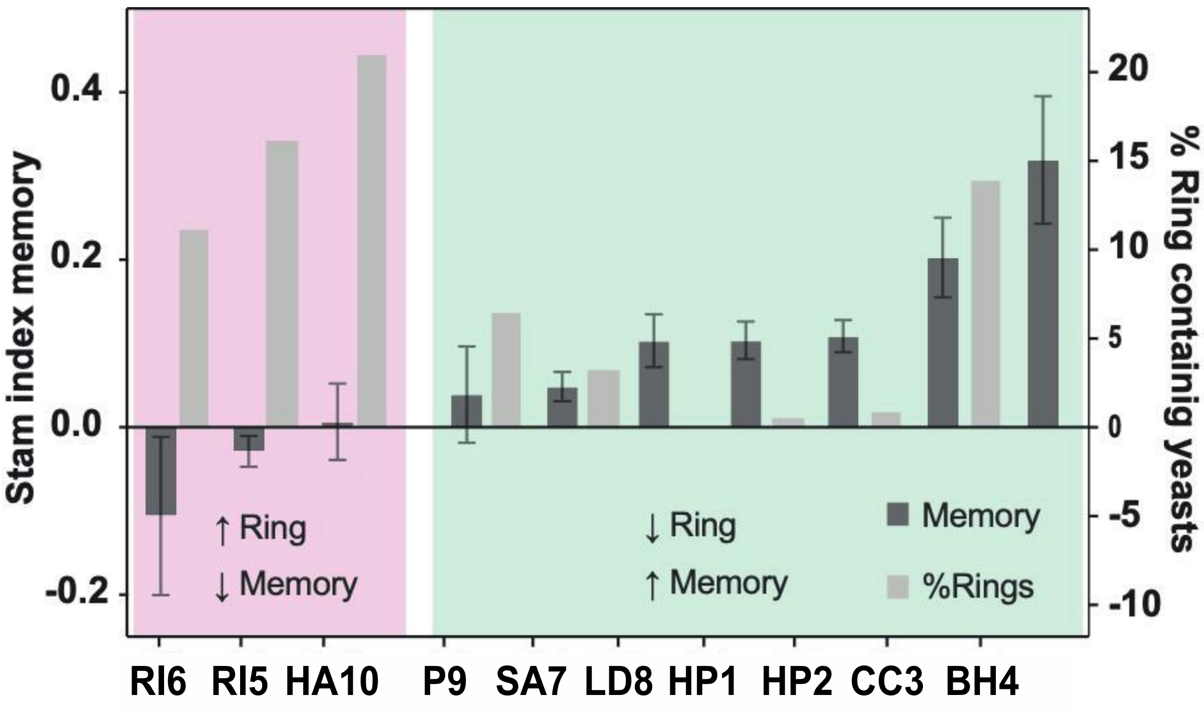

Appendix Figure S22. Memory index and presence of ring aggregates comparison. Plot comparing the memory index and the presence of ring structures in yeast (from Figure 4B) expressing different variants of Sup35NM.

Appendix Supplementary Data

Predictions performed on the Sup35p variants studied in this work.

Presented here are the sequences of the Sup35NM chimeras, with the newly detected sequences highlighted by pWALTZ (Material and Methods). Sequences in yellow indicate a precise match with the introduced amyloid-forming cores. Sequences in blue signify a partial match or mismatch with the introduced amyloid-forming cores, including the regions in Sup35NM and Sup35M highlighted in blue. The Sup35p nucleation region is denoted in bold and underlined. The introduced amyloid-forming cores are marked in bold.

List of pWALTZ detected sequences on the region detected by PAPA.

```
FULL  RGNYKNFNYNNNLQGYQAGFQ 73.9926 (residues from 98 to 118)
Delta RGNYKNFNYNNNLQGYQAGFQ 73.9926 (residues from 58 to 68)
HP1   GNNNNSVISFNQTNFNQGTYN 78.8660 (residues from 2 to 22)
HP2   NNNKSYYISAGGYYQNYQGYS 77.6716 (residues from 14 to 34)
CC3   MQAAFNFINNRRYQDAINVLN 78.1175 (residues from 2 to 22)
BH4   RNAQWYYLAGGYYQNYQGYSG 78.2290 (residues from 15 to 35)
RI5   YNNSYQNTNSYSAGGYYQNYQ 80.0127 (residues from 11 to 31)
RI6   MDNNYYNNNNNNNTNSNSNYN 77.1301 (residues from 2 to 22)
SA7   RSGNYVFLANSTNRYQGNYYN 75.7126 (residues from 2 to 22)
LD8   NQFQNSQYGSYQAGGYYQNYQ 77.5441 (residues from 11 to 31)
P9    GYGSNYNYSYSAAGGYYQNYQ 78.4679 (residues from 11 to 31)
HA10  TYNQSALINQIGAGGYYQNYQ 77.2256 (residues from 11 to 31)
```

In **bold** the 21 peptides studied.
In yellow when they matches 100% with the new pWALTZ prediction.
In blue when they doesn't match 100% with the new pWALTZ prediction
In **bold and underlined** the Sup35p nucleation region.

>Sup35NM
M**<u>SDSNQGNNQQNYQQYSQNGNQQQGNNRYQGYQAYNAQAQP</u>**AGGYYQNYQGYSGYQQGGYQQ
YNPDAGYQQQYNPQGGYQQYNPQGGYQQQFNPQGGRGNYKNFNYNNNLQGYQAGFQPQSQGM
SLNDFQKQQKQAAPKPKKTLKLVSSSGIKLANATKKVGTKPAESDKKEEEKSAETKEPTKEP
TKVEEPVKKEEKPVQTEEKTEEKSELPKVEDLKISESTHNTNNANVTSADALIKEQEEEVDD
EVVNDMFGGKDHVSLIFMGHVDAGKSTMGGNLLYLTGSVDKRTIEKYEREAKDAGRQGWYLS
WVMDTNKEERNDGKTIEVGKAYFETEKRRYTILDAPGHKMYVSEMIGGASQADVGVLVISAR
KGEYETGFERGGQTREHALLAKTQGVNKMVVVVNKMDDPTVNWSKERYDQCVSNVSNFLRAI
GYNIKTDVVFMPVSGYSGANLKDHVDPKECPWYTGPTLLEYLDTMNHVDRHINAPFMLPIAA
KMKDLGTIVEGKIESGHIKKGQSTLLMPNKTAVEIQNIYNETENEVDMAMCGEQVKLRIKGV
EEEDISPGFVLTSPKNPIKSVTKFVAQIAIVELKSIIAAGFSCVMHVHTAIEEVHIVKLLHK
LEKGTNRKSKKPPAFAKKGMKVIAVLETEAPVCVETYQDYPQLGRFTLRDQGTTIAIGKIVK
IAE

PAPA

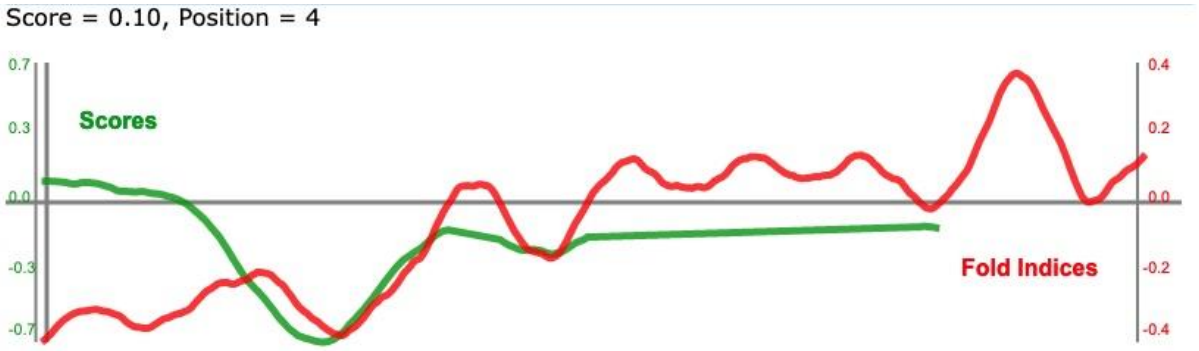

PLAAC

COREscore	LLR	PAPAprop	PAPAfi
23.306	23.306	0.100	-0.423

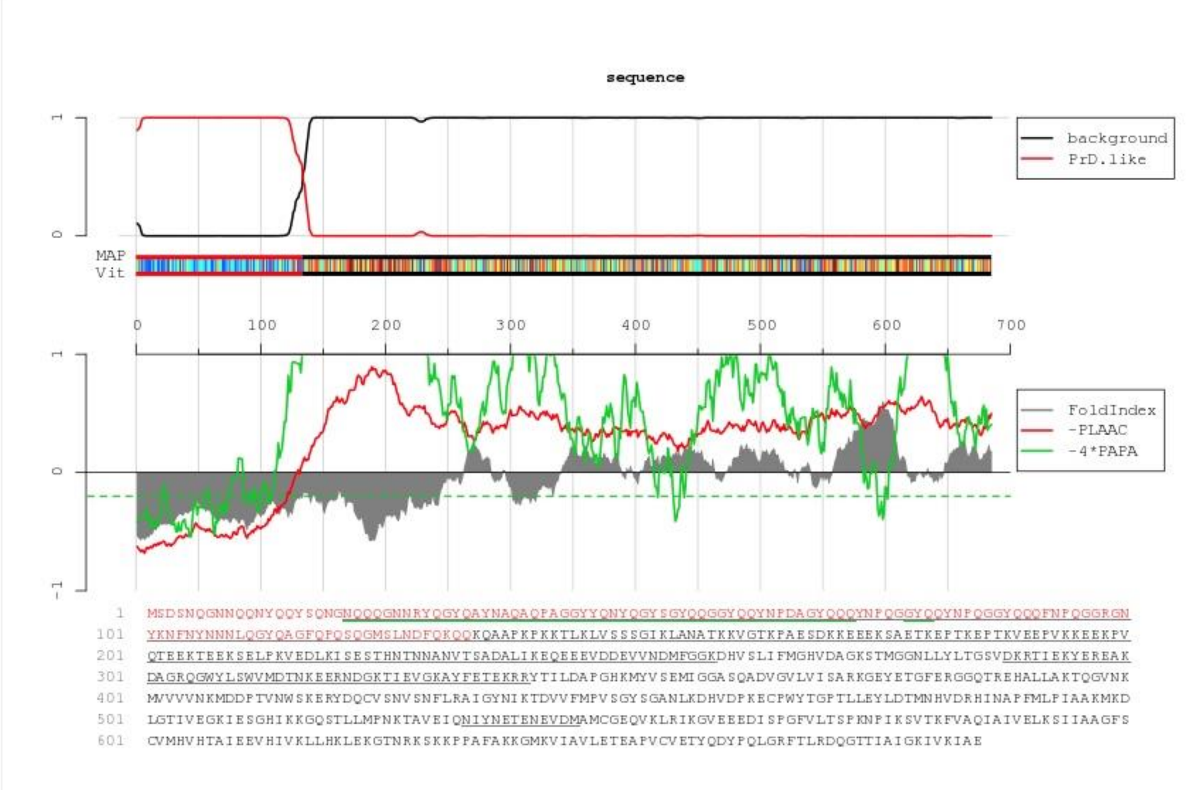

>Sup35M
MAGGYYQNYQGYSGYQQGGYQQYNPDAGYQQQYNPQGGYQQYNPQGGYQQQFNPQGGRGNYK
NFNYNNNLQGYQAGFQPQSQGMSLNDFQKQQKQAAPKPKKTLKLVSSSGIKLANATKKVGTK
PAESDKKEEEKSAETKEPTKEPTKVEEPVKKEEKPVQTEEKTEEKSELPKVEDLKISESTHN
TNNANVTSADALIKEQEEEVDDEVVNDMFGGKDHVSLIFMGHVDAGKSTMGGNLLYLTGSVD
KRTIEKYEREAKDAGRQGWYLSWVMDTNKEERNDGKTIEVGKAYFETEKRRYTILDAPGHKM
YVSEMIGGASQADVGVLVISARKGEYETGFERGGQTREHALLAKTQGVNKMVVVVNKMDDPT
VNWSKERYDQCVSNVSNFLRAIGYNIKTDVVFMPVSGYSGANLKDHVDPKECPWYTGPTLLE
YLDTMNHVDRHINAPFMLPIAAKMKDLGTIVEGKIESGHIKKGQSTLLMPNKTAVEIQNIYN
ETENEVDMAMCGEQVKLRIKGVEEEDISPGFVLTSPKNPIKSVTKFVAQIAIVELKSIIAAG
FSCVMHVHTAIEEVHIVKLLHKLEKGTNRKSKKPPAFAKKGMKVIAVLETEAPVCVETYQDY
PQLGRFTLRDQGTTIAIGKIVKIAE

PAPA

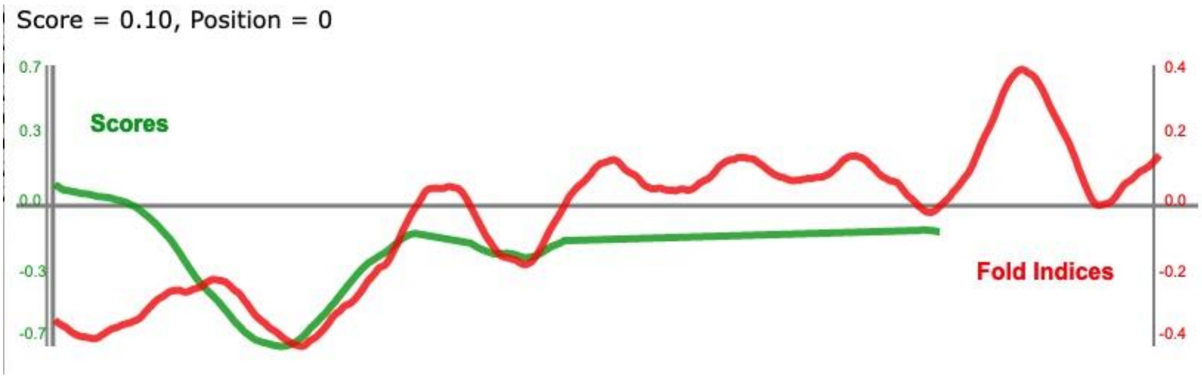

PLAAC

COREscore	LLR	PAPAprop	PAPAfi
19.025	19.025	0.099	-0.342

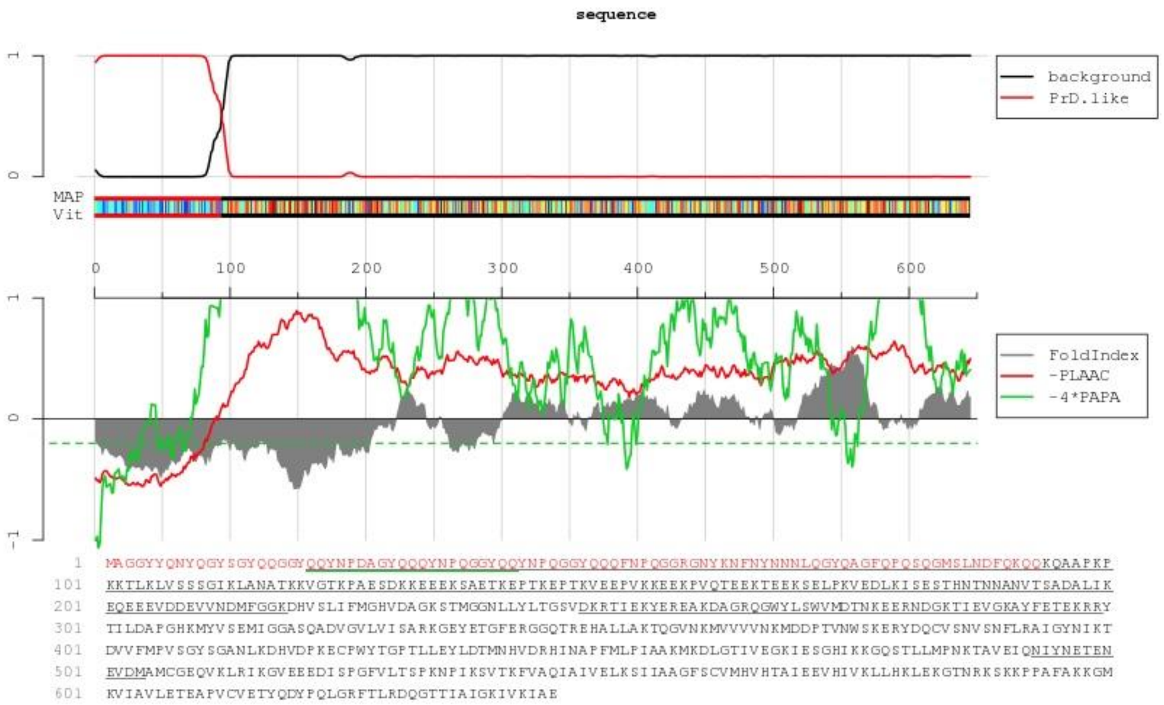

>HP1
MGNNNNSVISFNQTNFNQGTYNAGGYYQNYQGYSGYQQGGYQQYNPDAGYQQQYNPQGGYQQ
YNPQGGYQQQFNPQGGRGNYKNFNYNNNLQGYQAGFQPQSQGMSLNDFQKQQKQAAPKPKKT
LKLVSSSGIKLANATKKVGTKPAESDKKEEEKSAETKEPTKEPTKVEEPVKKEEKPVQTEEK
TEEKSELPKVEDLKISESTHNTNNANVTSADALIKEQEEEVDDEVVNDMFGGKDHVSLIFMG
HVDAGKSTMGGNLLYLTGSVDKRTIEKYEREAKDAGRQGWYLSWVMDTNKEERNDGKTIEVG
KAYFETEKRRYTILDAPGHKMYVSEMIGGASQADVGVLVISARKGEYETGFERGGQTREHAL
LAKTQGVNKMVVVVNKMDDPTVNWSKERYDQCVSNVSNFLRAIGYNIKTDVVFMPVSGYSGA
NLKDHVDPKECPWYTGPTLLEYLDTMNHVDRHINAPFMLPIAAKMKDLGTIVEGKIESGHIK
KGQSTLLMPNKTAVEIQNIYNETENEVDMAMCGEQVKLRIKGVEEEDISPGFVLTSPKNPIK
SVTKFVAQIAIVELKSIIAAGFSCVMHVHTAIEEVHIVKLLHKLEKGTNRKSKKPPAFAKKG
MKVIAVLETEAPVCVETYQDYPQLGRFTLRDQGTTIAIGKIVKIAE

PAPA

Score = 0.20, Position = 0

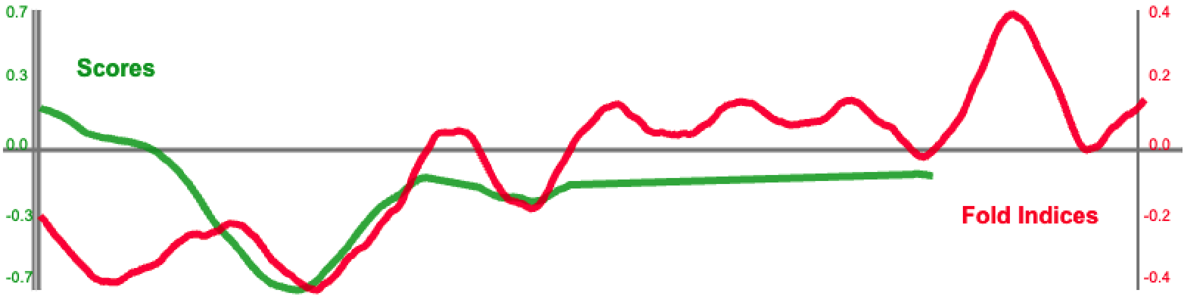

PLAAC

COREscore	LLR	PAPAprop	PAPAfi	(column descriptions)
19.025	19.025	0.195	-0.197	

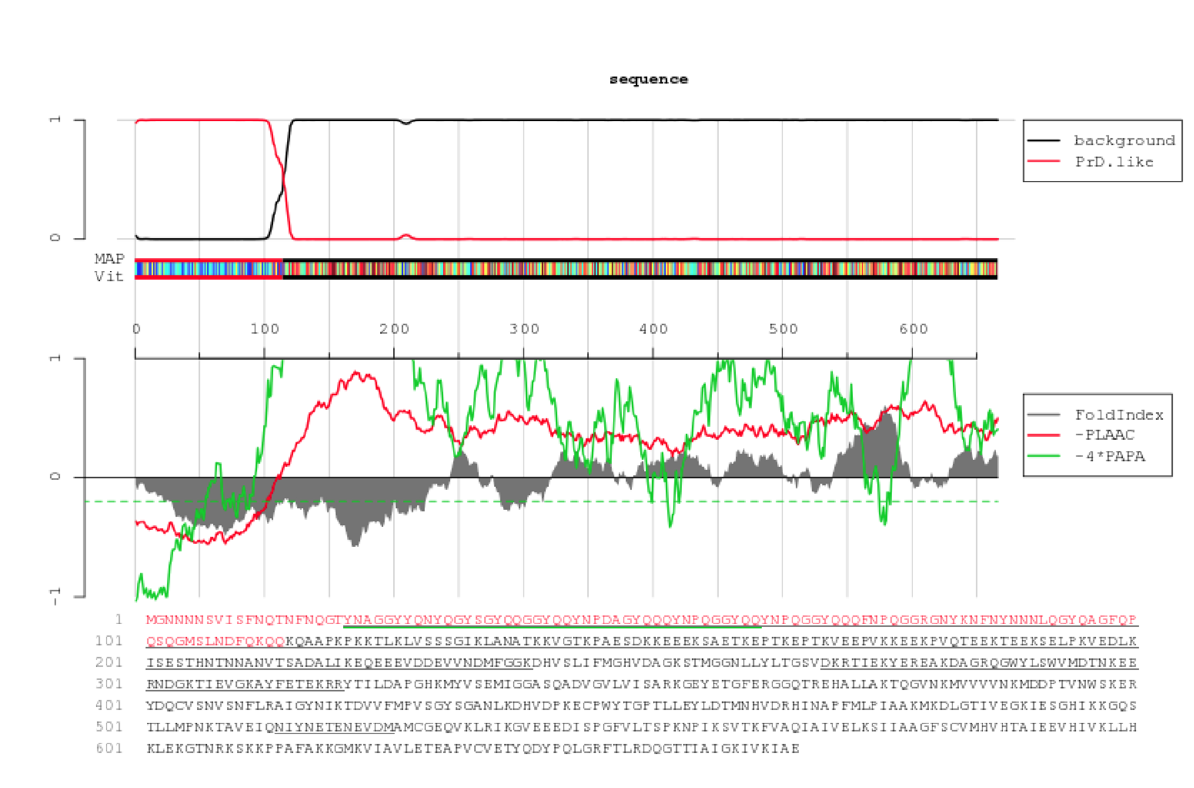

>HP2
MSSVYWLNSVNENNNNKSYYISAGGYYQNYQGYSGYQQGGYQQYNPDAGYQQQYNPQGGYQQ
YNPQGGYQQQFNPQGGRGNYKNFNYNNNLQGYQAGFQPQSQGMSLNDFQKQQKQAAPKPKKT
LKLVSSSGIKLANATKKVGTKPAESDKKEEEKSAETKEPTKEPTKVEEPVKKEEKPVQTEEK
TEEKSELPKVEDLKISESTHNTNNANVTSADALIKEQEEEVDDEVVNDMFGGKDHVSLIFMG
HVDAGKSTMGGNLLYLTGSVDKRTIEKYEREAKDAGRQGWYLSWVMDTNKEERNDGKTIEVG
KAYFETEKRRYTILDAPGHKMYVSEMIGGASQADVGVLVISARKGEYETGFERGGQTREHAL
LAKTQGVNKMVVVVNKMDDPTVNWSKERYDQCVSNVSNFLRAIGYNIKTDVVFMPVSGYSGA
NLKDHVDPKECPWYTGPTLLEYLDTMNHVDRHINAPFMLPIAAKMKDLGTIVEGKIESGHIK
KGQSTLLMPNKTAVEIQNIYNETENEVDMAMCGEQVKLRIKGVEEEDISPGFVLTSPKNPIK
SVTKFVAQIAIVELKSIIAAGFSCVMHVTAIEEVHIVKLLHKLEKGTNRKSKKPPAFAKKG
MKVIAVLETEAPVCVETYQDYPQLGRFTLRDQGTTIAIGKIVKIAE

PAPA

Score = 0.18, Position = 0

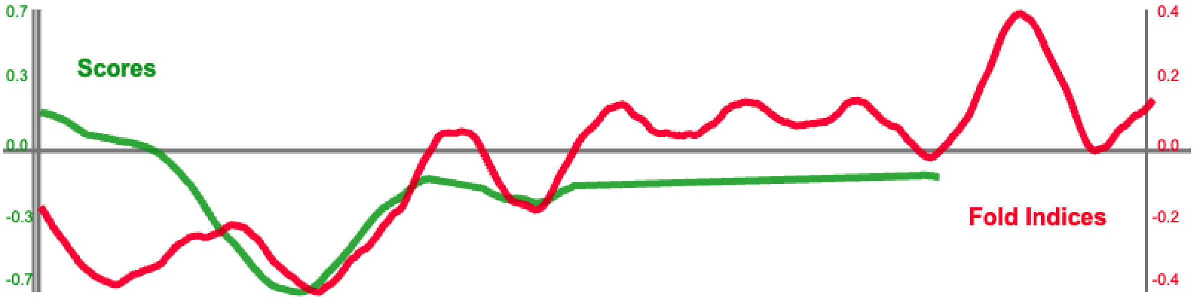

PLAAC

COREscore	LLR	PAPAprop	PAPAfi	(column descriptions)
19.025	19.025	0.180	-0.167	

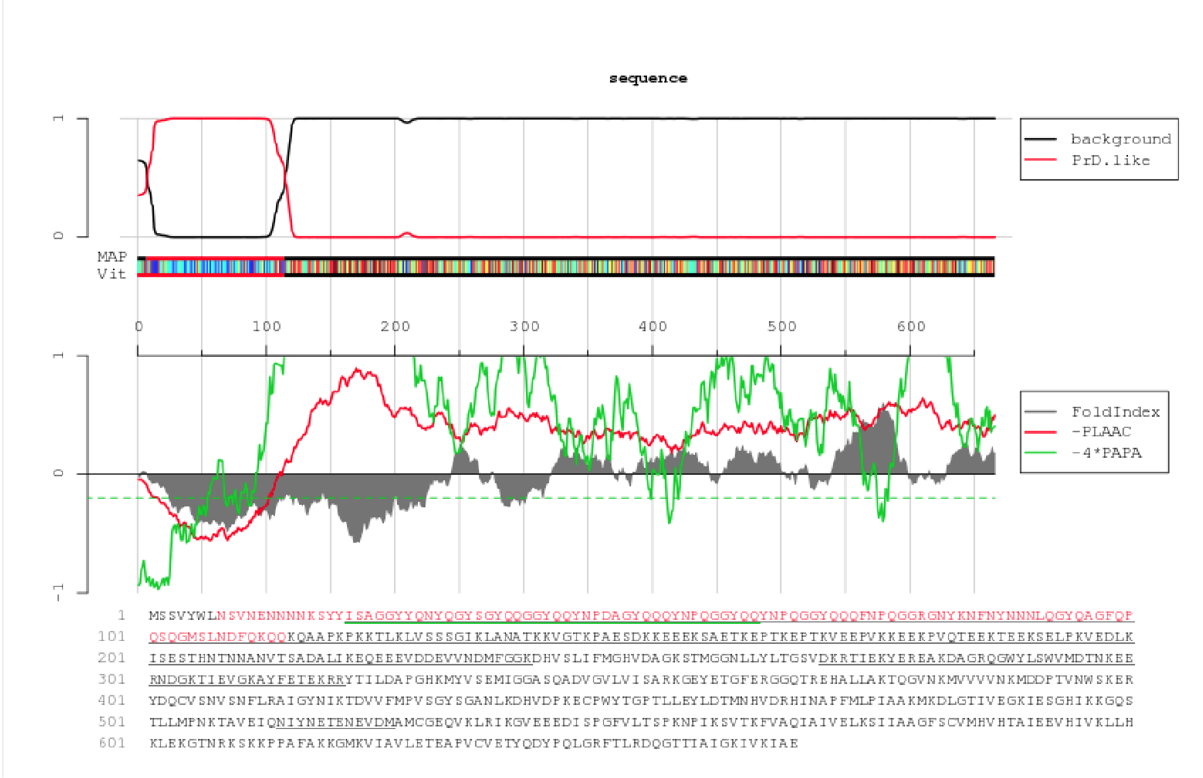

>CC3

M**MQAAFNFINNRRYQDAINVLN**AGGYYQNYQGYSGYQQGGYQQYNPDAGYQQQYNPQGGYQQ
YNPQGGYQQQFNPQGGRGNYKNFNYNNNLQGYQAGFQPQSQGMSLNDFQKQQKQAAPKPKKT
LKLVSSSGIKLANATKKVGTKPAESDKKEEEKSAETKEPTKEPTKVEEPVKKEEKPVQTEEK
TEEKSELPKVEDLKISESTHNTNNANVTSADALIKEQEEEVDDEVVNDMFGGKDHVSLIFMG
HVDAGKSTMGGNLLYLTGSVDKRTIEKYEREAKDAGRQGWYLSWVMDTNKEERNDGKTIEVG
KAYFETEKRRYTILDAPGHKMYVSEMIGGASQADVGVLVISARKGEYETGFERGGQTREHAL
LAKTQGVNKMVVVVNKMDDPTVNWSKERYDQCVSNVSNFLRAIGYNIKTDVVFMPVSGYSGA
NLKDHVDPKECPWYTGPTLLEYLDTMNHVDRHINAPFMLPIAAKMKDLGTIVEGKIESGHIK
KGQSTLLMPNKTAVEIQNIYNETENEVDMAMCGEQVKLRIKGVEEEDISPGFVLTSPKNPIK

SVTKFVAQIAIVELKSIIAAGFSCVMHVHTAIEEVHIVKLLHKLEKGTNRKSKKPPAFAKKG
MKVIAVLETEAPVCVETYQDYPQLGRFTLRDQGTTIAIGKIVKIAE

PAPA

Score = 0.15, Position = 0

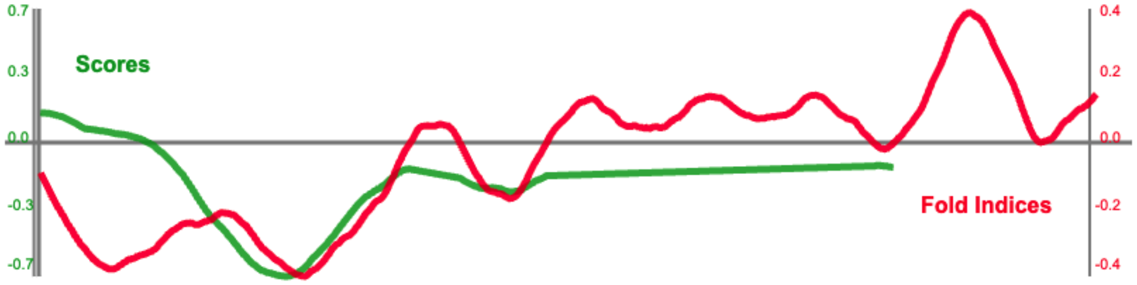

PLAAC

COREscore	LLR	PAPAprop	PAPAfi	(column descriptions)
19.025	19.025	0.146	-0.095	

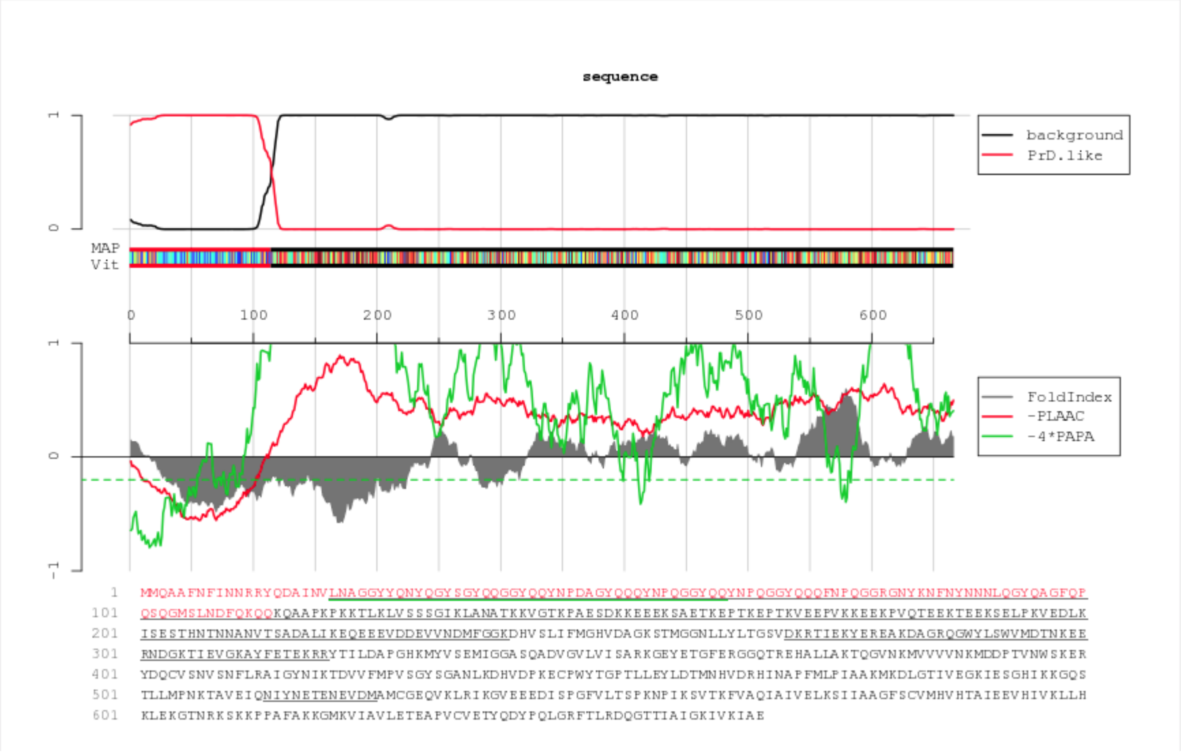

>BH4
MYQQALNVLSRIQNRNAQWYYLAGGYYQNYQGYSGYQQGGYQQYNPDAGYQQQYNPQGGYQQ
YNPQGGYQQQFNPQGGRGNYKNFNYNNNLQGYQAGFQPQSQGMSLNDFQKQQKQAAPKPKKT
LKLVSSSGIKLANATKKVGTKPAESDKKEEEKSAETKEPTKEPTKVEEPVKKEEKPVQTEEK
TEEKSELPKVEDLKISESTHNTNNANVTSADALIKEQEEEVDDEVVNDMFGGKDHVSLIFMG
HVDAGKSTMGGNLLYLTGSVDKRTIEKYEREAKDAGRQGWYLSWVMDTNKEERNDGKTIEVG
KAYFETEKRRYTILDAPGHKMYVSEMIGGASQADVGVLVISARKGEYETGFERGGQTREHAL

LAKTQGVNKMVVVVNKMDDPTVNWSKERYDQCVSNVSNFLRAIGYNIKTDVVFMPVSGYSGA
NLKDHVDPKECPWYTGPTLLEYLDTMNHVDRHINAPFMLPIAAKMKDLGTIVEGKIESGHIK
KGQSTLLMPNKTAVEIQNIYNETENEVDMAMCGEQVKLRIKGVEEEDISPGFVLTSPKNPIK
SVTKFVAQIAIVELKSIIAAGFSCVMHVHTAIEEVHIVKLLHKLEKGTNRKSKKPPAFAKKG
MKVIAVLETEAPVCVETYQDYPQLGRFTLRDQGTTIAIGKIVKIAE

PAPA

Score = 0.17, Position = 0

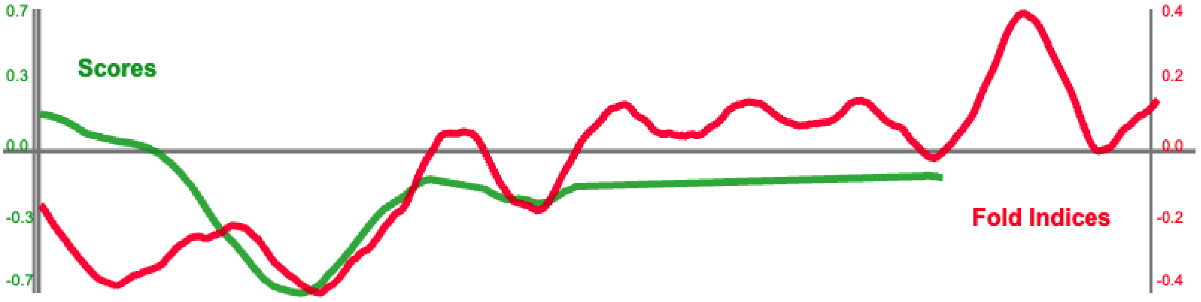

PLAAC

COREscore	LLR	PAPAprop	PAPAfi	(column descriptions)
19.025	19.025	0.171	-0.161	

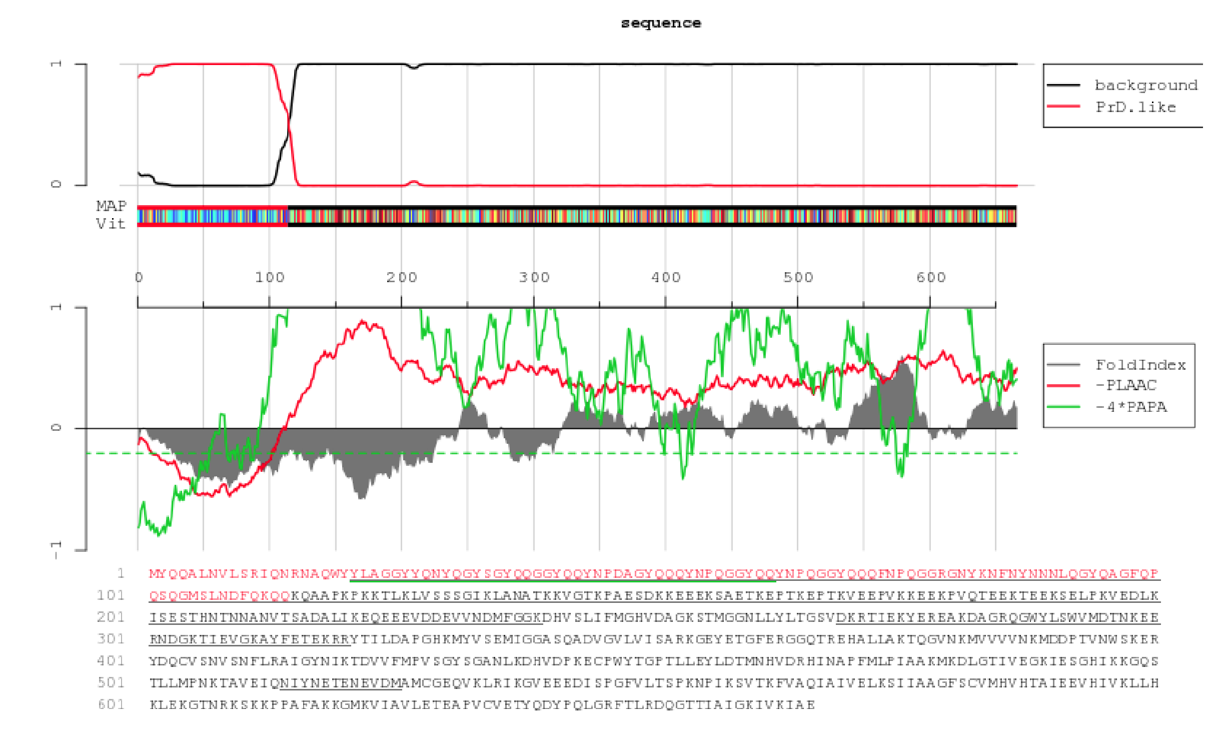

>RI5

MHANEGNVNQYNNSYQNTNSYSAGGYYQNYQGYSGYQQGGYQQYNPDAGYQQQYNPQGGYQQ
YNPQGGYQQQFNPQGGRGNYKNFNYNNNLQGYQAGFQPQSQGMSLNDFQKQQKQAAPKPKKT
LKLVSSSGIKLANATKKVGTKPAESDKKEEEKSAETKEPTKEPTKVEEPVKKEEKPVQTEEK

TEEKSELPKVEDLKISESTHNTNNANVTSADALIKEQEEEVDDEVVNDMFGGKDHVSLIFMG
HVDAGKSTMGGNLLYLTGSVDKRTIEKYEREAKDAGRQGWYLSWVMDTNKEERNDGKTIEVG
KAYFETEKRRYTILDAPGHKMYVSEMIGGASQADVGVLVISARKGEYETGFERGGQTREHAL
LAKTQGVNKMVVVVNKMDDPTVNWSKERYDQCVSNVSNFLRAIGYNIKTDVVFMPVSGYSGA
NLKDHVDPKECPWYTGPTLLEYLDTMNHVDRHINAPFMLPIAAKMKDLGTIVEGKIESGHIK
KGQSTLLMPNKTAVEIQNIYNETENEVDMAMCGEQVKLRIKGVEEEDISPGFVLTSPKNPIK
SVTKFVAQIAIVELKSIIAAGFSCVMHVHTAIEEVHIVKLLHKLEKGTNRKSKKPPAFAKKG
MKVIAVLETEAPVCVETYQDYPQLGRFTLRDQGTTIAIGKIVKIAE

PAPA

Score = 0.16, Position = 0

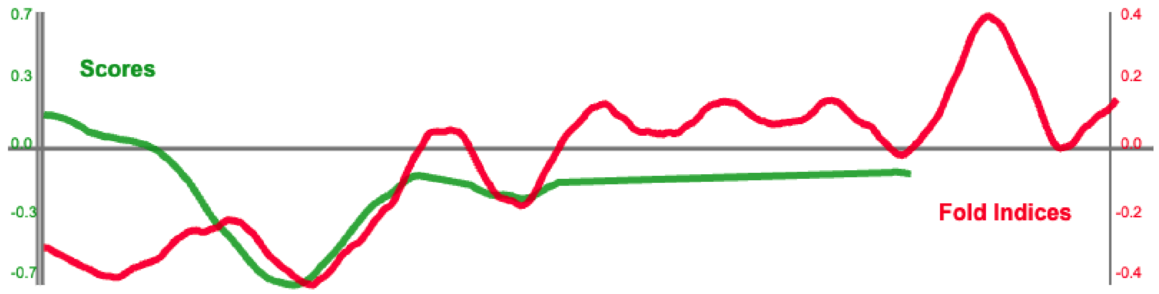

PLAAC

COREscore	LLR	PAPAprop	PAPAfi	(column descriptions)
19.025	19.025	0.163	-0.305	

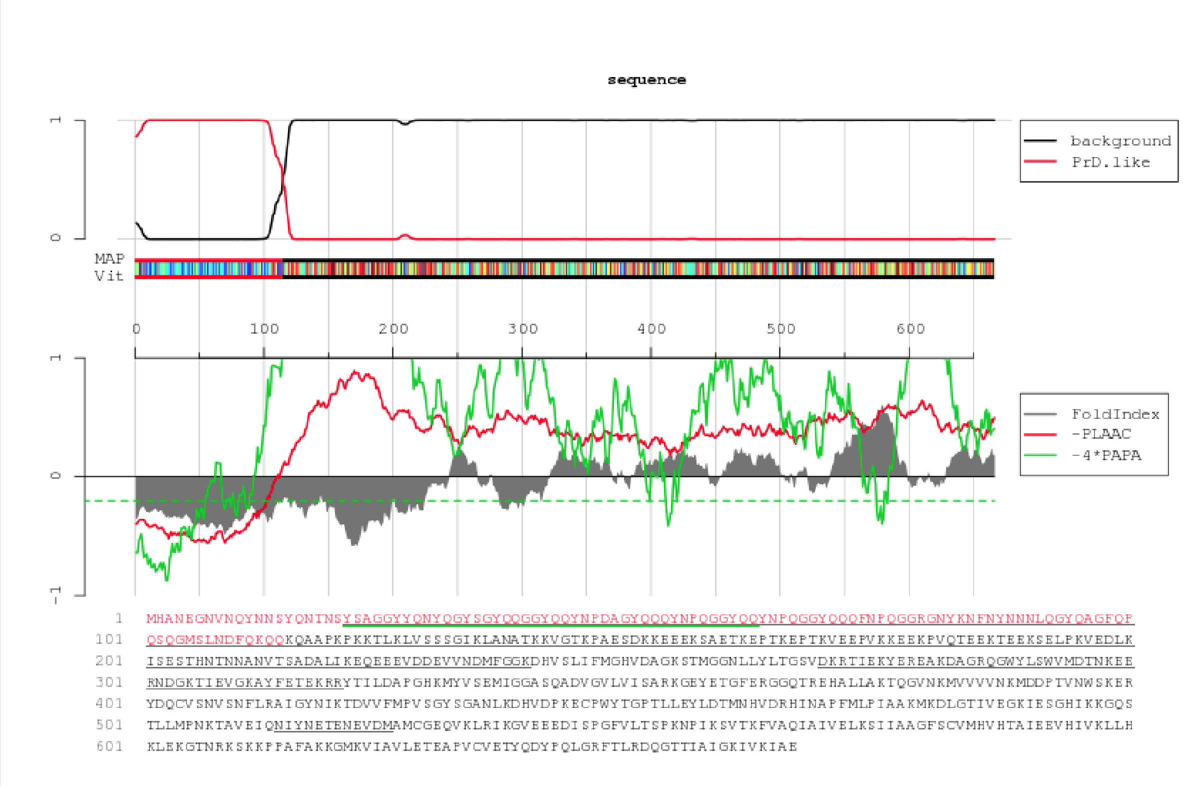

>RI6

M**MDNNYYNNNNNNNNTNSNSNYN**AGGYYQNYQGYSGYQQGGYQQYNPDAGYQQQYNPQGGYQQ
YNPQGGYQQQFNPQGGRGNYKNFNYNNNLQGYQAGFQPQSQGMSLNDFQKQQKQAAPKPKKT
LKLVSSSGIKLANATKKVGTKPAESDKKEEEKSAETKEPTKEPTKVEEPVKKEEKPVQTEEK
TEEKSELPKVEDLKISESTHNTNNANVTSADALIKEQEEEVDDEVVNDMFGGKDHVSLIFMG
HVDAGKSTMGGNLLYLTGSVDKRTIEKYEREAKDAGRQGWYLSWVMDTNKEERNDGKTIEVG
KAYFETEKRRYTILDAPGHKMYVSEMIGGASQADVGVLVISARKGEYETGFERGGQTREHAL
LAKTQGVNKMVVVVNKMDDPTVNWSKERYDQCVSNVSNFLRAIGYNIKTDVVFMPVSGYSGA
NLKDHVDPKECPWYTGPTLLEYLDTMNHVDRHINAPFMLPIAAKMKDLGTIVEGKIESGHIK
KGQSTLLMPNKTAVEIQNIYNETENEVDMAMCGEQVKLRIKGVEEEDISPGFVLTSPKNPIK
SVTKFVAQIAIVELKSIIAAGFSCVMHVHTAIEEVHIVKLLHKLEKGTNRKSKKPPAFAKKG
MKVIAVLETEAPVCVETYQDYPQLGRFTLRDQGTTIAIGKIVKIAE

PAPA

Score = 0.16, Position = 0

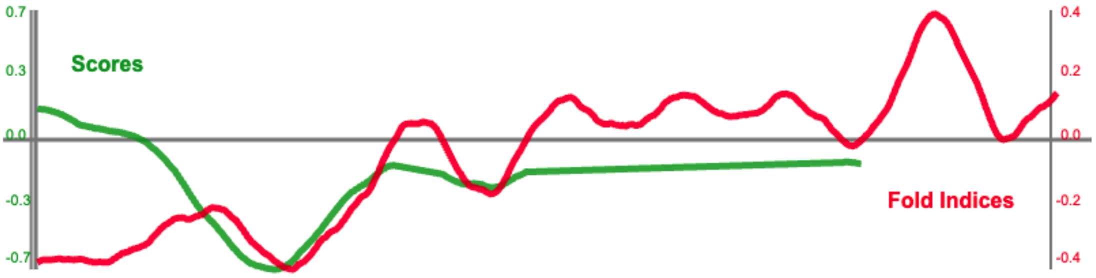

PLAAC

sequence

COREscore	LLR	PAPAprop	PAPAfi	(column descriptions)
20.492	20.492	0.157	-0.398	

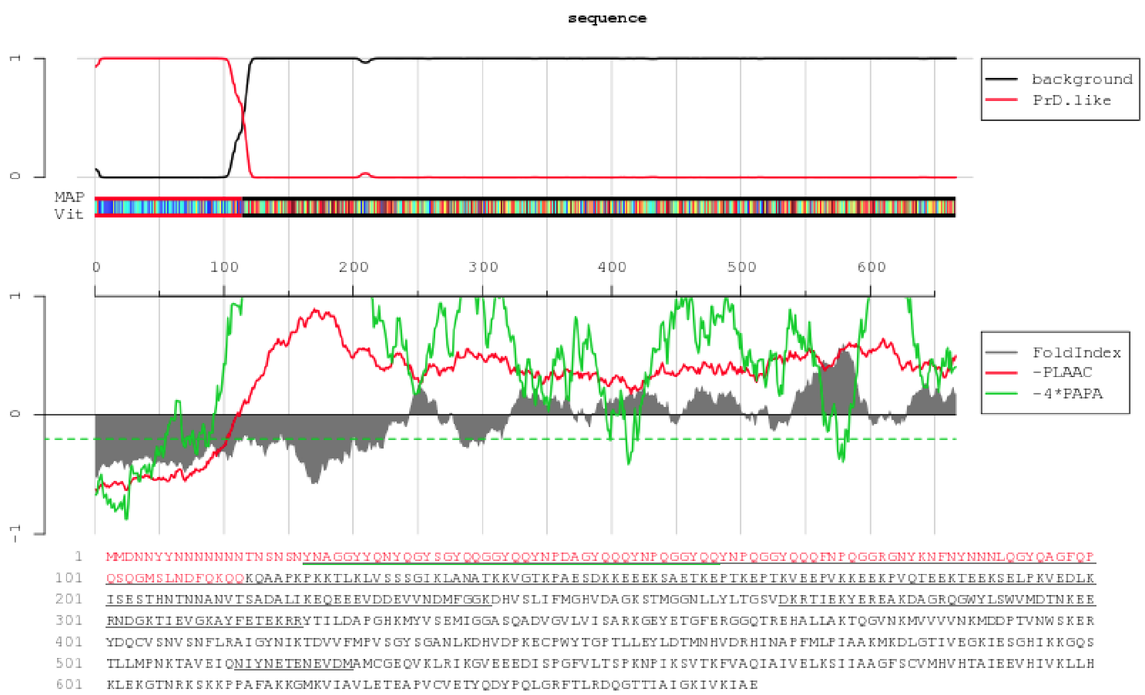

>SA7
MRSGNYVFLANSTNRYQGNYYNAGGYYQNYQGYSGYQQGGYQQYNPDAGYQQQYNPQGGYQQ
YNPQGGYQQQFNPQGGRGNYKNFNYNNNLQGYQAGFQPQSQGMSLNDFQKQQKQAAPKPKKT
LKLVSSSGIKLANATKKVGTKPAESDKKEEEKSAETKEPTKEPTKVEEPVKKEEKPVQTEEK
TEEKSELPKVEDLKISESTHNTNNANVTSADALIKEQEEEVDDEVVNDMFGGKDHVSLIFMG
HVDAGKSTMGGNLLYLTGSVDKRTIEKYEREAKDAGRQGWYLSWVMDTNKEERNDGKTIEVG
KAYFETEKRRYTILDAPGHKMYVSEMIGGASQADVGVLVISARKGEYETGFERGGQTREHAL
LAKTQGVNKMVVVVNKMDDPTVNWSKERYDQCVSNVSNFLRAIGYNIKTDVVFMPVSGYSGA
NLKDHVDPKECPWYTGPTLLEYLDTMNHVDRHINAPFMLPIAAKMKDLGTIVEGKIESGHIK
KGQSTLLMPNKTAVEIQNIYNETENEVDMAMCGEQVKLRIKGVEEEDISPGFVLTSPKNPIK
SVTKFVAQIAIVELKSIIAAGFSCVMHVHTAIEEVHIVKLLHKLEKGTNRKSKKPPAFAKKG
MKVIAVLETEAPVCVETYQDYPQLGRFTLRDQGTTIAIGKIVKIAE

PAPA

Score = 0.18, Position = 0

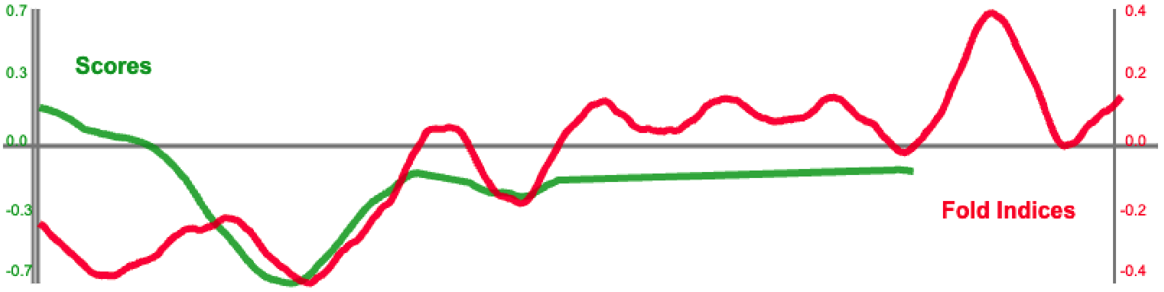

PLAAC

COREscore	LLR	PAPAprop	PAPAfi	(column descriptions)
19.025	19.025	0.183	-0.237	

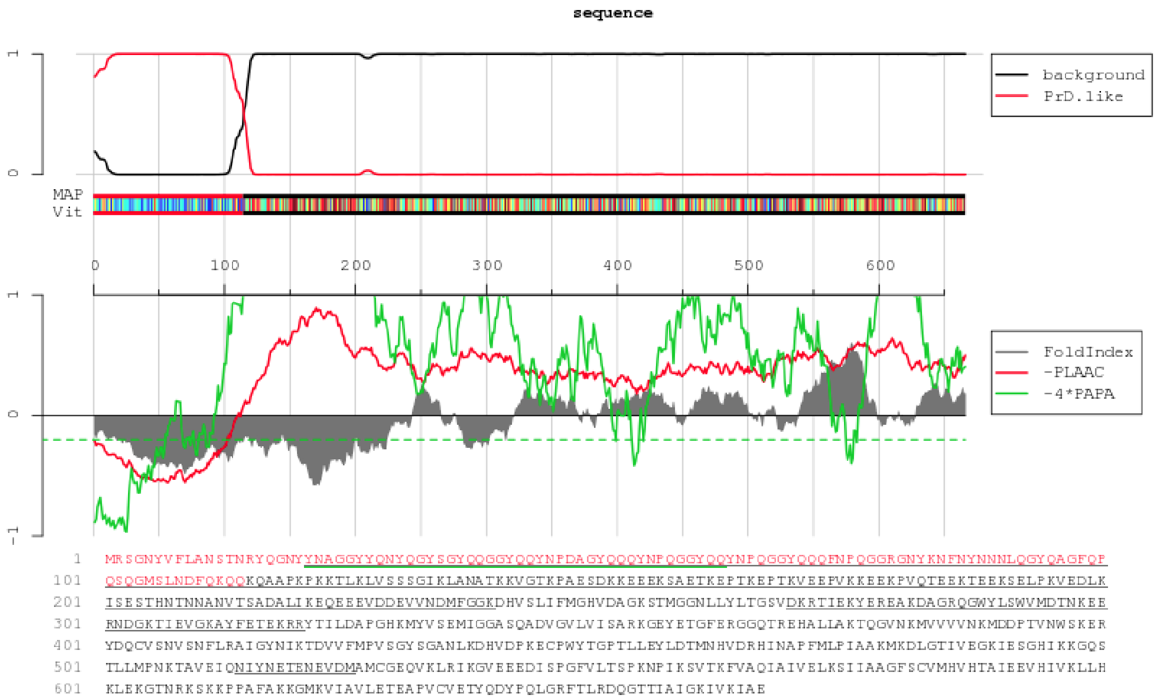

>LD8
MSYSSSANNYGYGSNYNYSYSAAGGYYQNYQGYSGYQQGGYQQYNPDAGYQQQYNPQGGYQQ
YNPQGGYQQQFNPQGGRGNYKNFNYNNNLQGYQAGFQPQSQGMSLNDFQKQQKQAAPKPKKT
LKLVSSSGIKLANATKKVGTKPAESDKKEEEKSAETKEPTKEPTKVEEPVKKEEKPVQTEEK
TEEKSELPKVEDLKISESTHNTNNANVTSADALIKEQEEEVDDEVVNDMFGGKDHVSLIFMG
HVDAGKSTMGGNLLYLTGSVDKRTIEKYEREAKDAGRQGWYLSWVMDTNKEERNDGKTIEVG
KAYFETEKRRYTILDAPGHKMYVSEMIGGASQADVGVLVISARKGEYETGFERGGQTREHAL
LAKTQGVNKMVVVVNKMDDPTVNWSKERYDQCVSNVSNFLRAIGYNIKTDVVFMPVSGYSGA
NLKDHVDPKECPWYTGPTLLEYLDTMNHVDRHINAPFMLPIAAKMKDLGTIVEGKIESGHIK
KGQSTLLMPNKTAVEIQNIYNETENEVDMAMCGEQVKLRIKGVEEEDISPGFVLTSPKNPIK
SVTKFVAQIAIVELKSIIAAGFSCVMHVHTAIEEVHIVKLLHKLEKGTNRKSKKPPAFAKKG
MKVIAVLETEAPVCVETYQDYPQLGRFTLRDQGTTIAIGKIVKIAE

PAPA

Score = 0.20, Position = 0

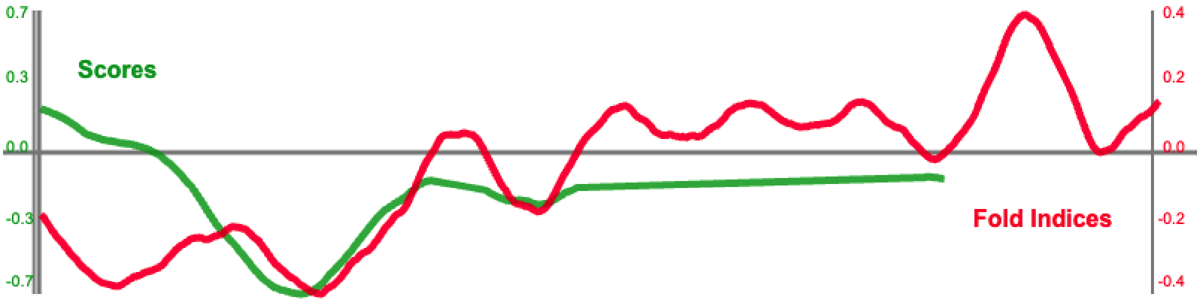

PLAAC

COREscore	LLR	PAPAprop	PAPAfi	(column descriptions)
19.025	19.025	0.201	-0.186	

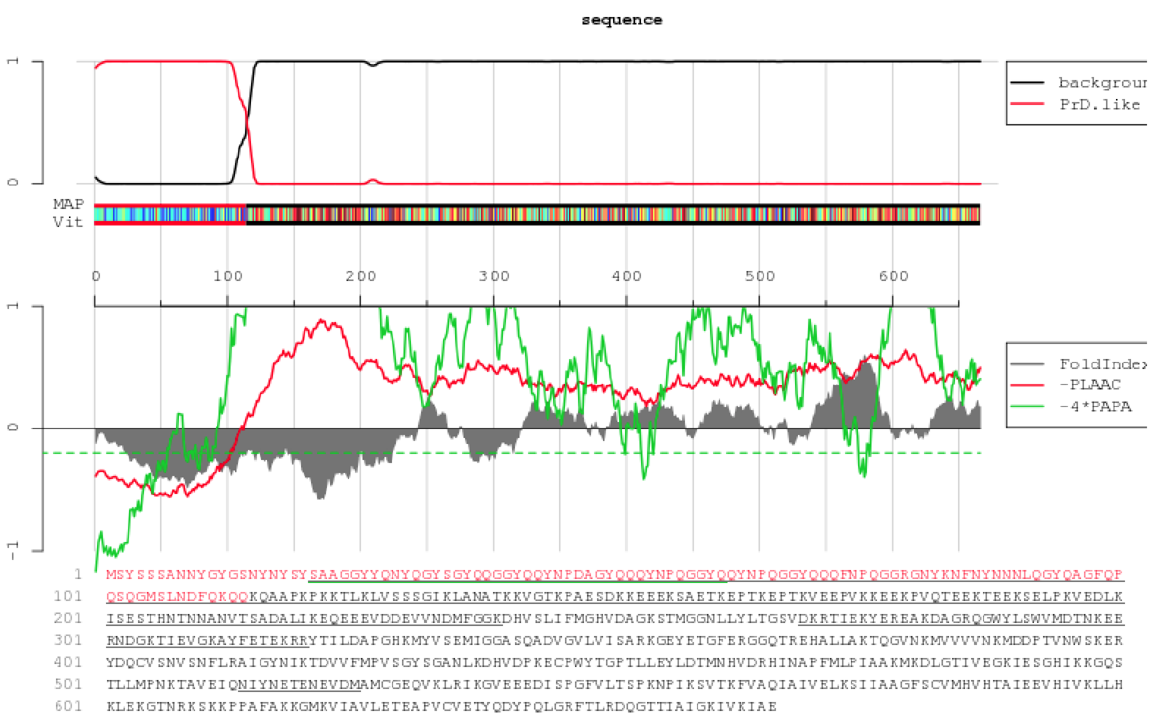

>P9

MGYVQSSYGQNQFQNSQYGSYQAGGYYQNYQGYSGYQQGGYQQYNPDAGYQQQYNPQGGYQQ
YNPQGGYQQQFNPQGGRGNYKNFNYNNNLQGYQAGFQPQSQGMSLNDFQKQQKQAAPKPKKT
LKLVSSSGIKLANATKKVGTKPAESDKKEEEKSAETKEPTKEPTKVEEPVKKEEKPVQTEEK
TEEKSELPKVEDLKISESTHNTNNANVTSADALIKEQEEEVDDEVVNDMFGGKDHVSLIFMG
HVDAGKSTMGGNLLYLTGSVDKRTIEKYEREAKDAGRQGWYLSWVMDTNKEERNDGKTIEVG
KAYFETEKRRYTILDAPGHKMYVSEMIGGASQADVGVLVISARKGEYETGFERGGQTREHAL
LAKTQGVNKMVVVVNKMDDPTVNWSKERYDQCVSNVSNFLRAIGYNIKTDVVFMPVSGYSGA
NLKDHVDPKECPWYTGPTLLEYLDTMNHVDRHINAPFMLPIAAKMKDLGTIVEGKIESGHIK
KGQSTLLMPNKTAVEIQNIYNETENEVDMAMCGEQVKLRIKGVEEEDISPGFVLTSPKNPIK

SVTKFVAQIAIVELKSIIAAGFSCVMHVHTAIEEVHIVKLLHKLEKGTNRKSKKPPAFAKKG
MKVIAVLETEAPVCVETYQDYPQLGRFTLRDQGTTIAIGKIVKIAE

PAPA

Score = 0.21, Position = 0

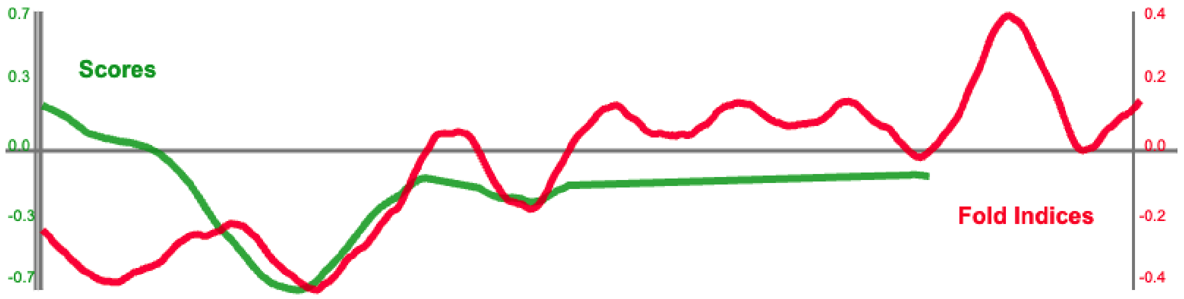

PLAAC

COREscore	LLR	PAPAprop	PAPAfi	(column descriptions)
19.025	19.025	0.213	-0.240	

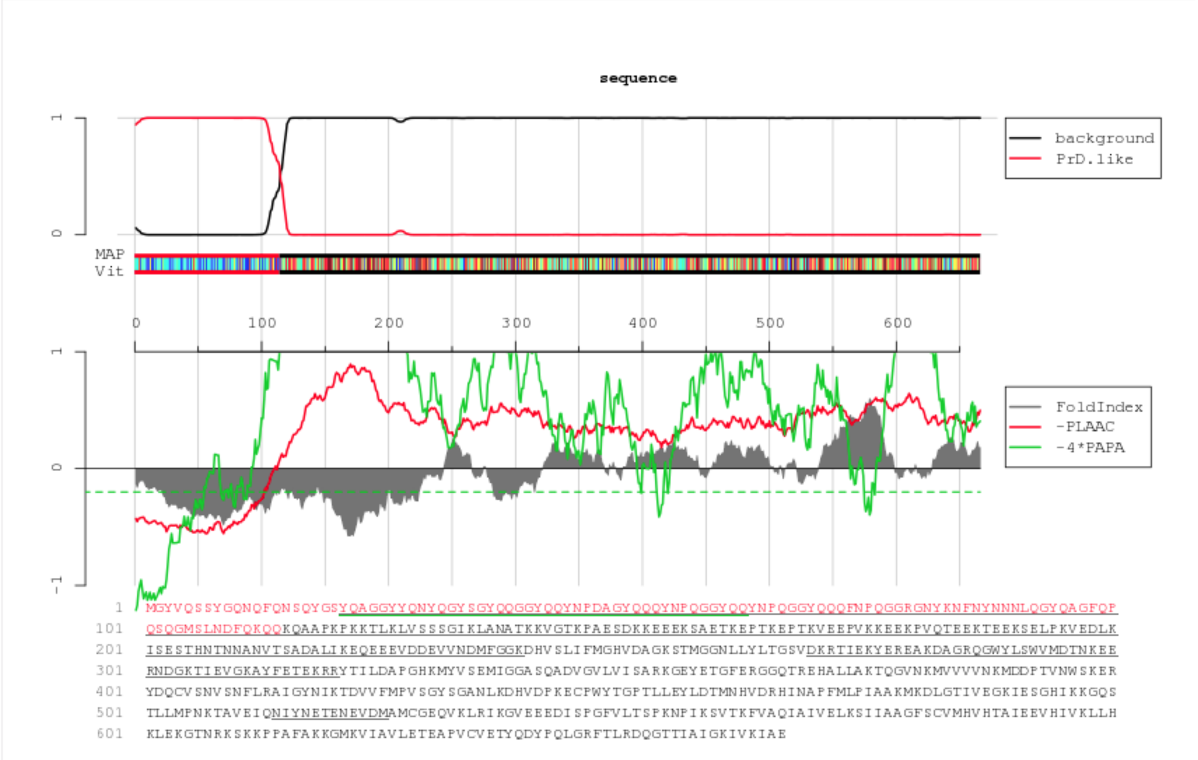

>HA10
MSTGYSDLNSTYNQSALINQIGAGGYYQNYQGYSGYQQGGYQQYNPDAGYQQQYNPQGGYQQ
YNPQGGYQQQFNPQGGRGNYKNFNYNNNLQGYQAGFQPQSQGMSLNDFQKQQKQAAPKPKKT
LKLVSSSGIKLANATKKVGTKPAESDKKEEEKSAETKEPTKEPTKVEEPVKKEEKPVQTEEK
TEEKSELPKVEDLKISESTHNTNNANVTSADALIKEQEEEVDDEVVNDMFGGKDHVSLIFMG
HVDAGKSTMGGNLLYLTGSVDKRTIEKYEREAKDAGRQGWYLSWVMDTNKEERNDGKTIEVG
KAYFETEKRRYTILDAPGHKMYVSEMIGGASQADVGVLVISARKGEYETGFERGGQTREHAL
LAKTQGVNKMVVVVNKMDDPTVNWSKERYDQCVSNVSNFLRAIGYNIKTDVVFMPVSGYSGA
NLKDHVDPKECPWYTGPTLLEYLDTMNHVDRHINAPFMLPIAAKMKDLGTIVEGKIESGHIK

KGQSTLLMPNKTAVEIQNIYNETENEVDMAMCGEQVKLRIKGVEEEDISPGFVLTSPKNPIK
SVTKFVAQIAIVELKSIIAAGFSCVMHVHTAIEEVHIVKLLHKLEKGTNRKSKKPPAFAKKG
MKVIAVLETEAPVCVETYQDYPQLGRFTLRDQGTTIAIGKIVKIAE

PAPA

Score = 0.14, Position = 0

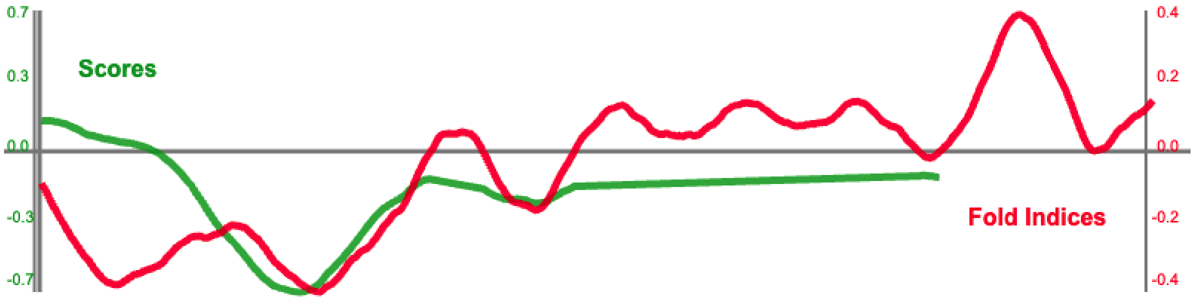

PLAAC

COREscore	LLR	PAPAprop	PAPAfi	(column descriptions)
19.025	19.025	0.142	-0.096	

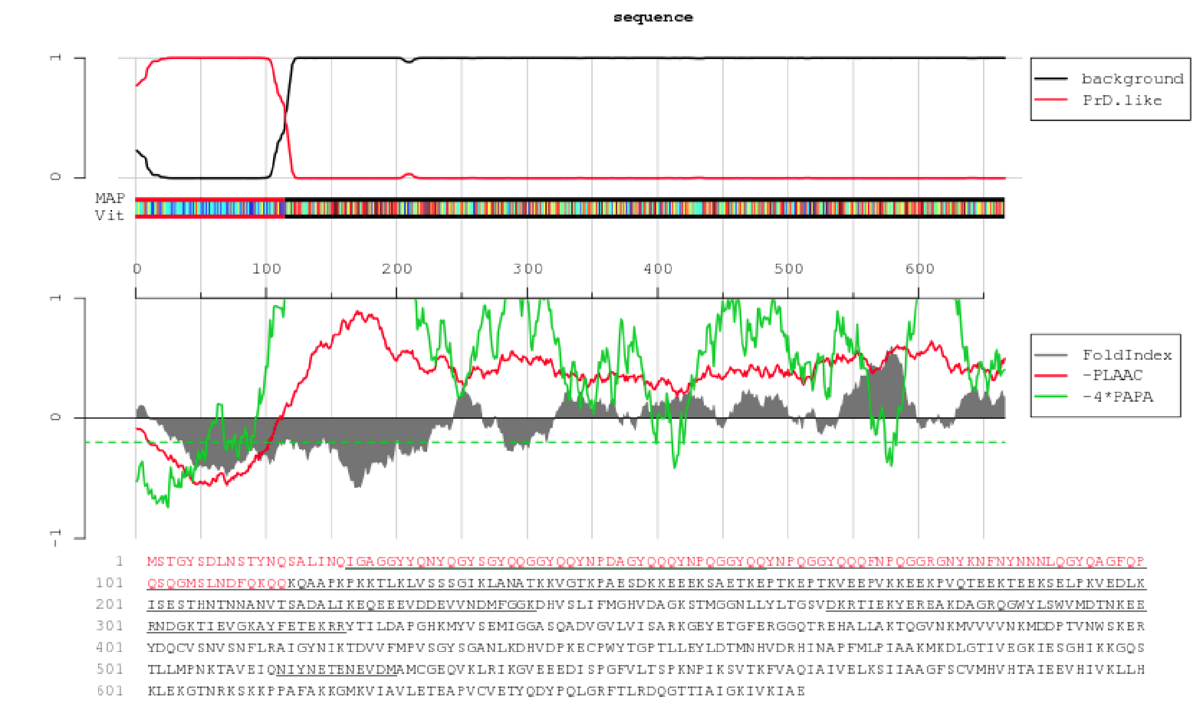

Protein sequences of the Sup35p and the Sup35NM fused to GFP expressed in *S. cerevisiae.*

Sup35p

MSDSNQGNNQQNYQQYSQNGNQQQGNNRYQGYQAYNAQAQPAGGYYQNYQGYSGYQQGGYQQ
YNPDAGYQQQYNPQGGYQQYNPQGGYQQQFNPQGGRGNYKNFNYNNNLQGYQAGFQPQSQGM
SLNDFQKQQKQAAPKPKKTLKLVSSSGIKLANATKKVGTKPAESDKKEEEKSAETKEPTKEP
TKVEEPVKKEEKPVQTEEKTEEKSELPKVEDLKISESTHNTNNANVTSADALIKEQEEEVDD
EVVNDMFGGKDHVSLIFMGHVDAGKSTMGGNLLYLTGSVDKRTIEKYEREAKDAGRQGWYLS
WVMDTNKEERNDGKTIEVGKAYFETEKRRYTILDAPGHKMYVSEMIGGASQADVGVLVISAR
KGEYETGFERGGQTREHALLAKTQGVNKMVVVVNKMDDPTVNWSKERYDQCVSNVSNFLRAI
GYNIKTDVVFMPVSGYSGANLKDHVDPKECPWYTGPTLLEYLDTMNHVDRHINAPFMLPIAA
KMKDLGTIVEGKIESGHIKKGQSTLLMPNKTAVEIQNIYNETENEVDMAMCGEQVKLRIKGV
EEEDISPGFVLTSPKNPIKSVTKFVAQIAIVELKSIIAAGFSCVMHVHTAIEEVHIVKLLHK
LEKGTNRKSKKPPAFAKKGMKVIAVLETEAPVCVETYQDYPQLGRFTLRDQGTTIAIGKIVK
IAE

Red: amyloid core substituted by the bacterial peptides; purple: N-domain; brown: M-domain; blue: C-terminal domain

NMGFP fusion

MSDSNQGNNQQNYQQYSQNGNQQQGNNRYQGYQAYNAQAQPAGGYYQNYQGYSGYQQGGYQQ
YNPDAGYQQQYNPQGGYQQYNPQGGYQQQFNPQGGRGNYKNFNYNNNLQGYQAGFQPQSQGM
SLNDFQKQQKQAAPKPKKTLKLVSSSGIKLANATKKVGTKPAESDKKEEEKSAETKEPTKEP
TKVEEPVKKEEKPVQTEEKTEEKSELPKVEDLKISESTHNTNNANVTSADALIKEQEEEVDD
EVVNDMFGSAGSAAGSGMSRSKGEELFTGVVPILVELDGDVNGHKFSVSGEGEGDATYGKLT
LKFICTTGKLPVPWPTLVTTLTYGVQCFSRYPDHMKRHDFFKSAMPEGYVQERTISFKDDGN
YKTRAEVKFEGDTLVNRIELKGIDFKEDGNILGHKLEYNYNSHNVYITADKQKNGIKANFKI
RHNIEDGSVQLADHYQQNTPIGDGPVLLPDNHYLSTQSALSKDPNEKRDHMVLLEFVTAAGI
THGMDELYKDSHHVSMDYKDDDDKI

Red: amyloid core substituted by the bacterial peptides; purple: N-domain; brown: M-domain; cyan: linker; green: GFP

Protein sequences of the constructs encoding the Sup35p variants (Sup35NM, DNM and chimeras) expressed in *E. coli.*

Green: First methionine
Pink: CsgAss signal sequence
Black: linker (Not1+serine)
Blue bold: The nucleation region
Blue: Sup35NM
Red: The amyloid-cores
Black bold: 6xHistidines

Sup35NM

MKLLKVAAIAAIVFSGSALAGVVPQYGGGGNHGGGGNNSGPNAAAS**DSNQGNNQQNYQQYSQ**
NGNQQQGNNRYQGYQAYNAQAQPAGGYYQNYQGYSGYQQGGYQQYNPDAGYQQQYNPQGGYQ
QYNPQGGYQQQFNPQGGRGNYKNFNYNNNLQGYQAGFQPQSQGMSLNDFQKQQKQAAPKPKK
TLKLVSSSGIKLANATKKVGTKPAESDKKEEEKSAETKEPTKEPTKVEEPVKKEEKPVQTEE
KTEEKSELPKVEDLKISESTHNTNNANVTSADALIKEQEEEVDDEVVND**HHHHHH**

ΔSup35NM

MKLLKVAAIAAIVFSGSALAGVVPQYGGGGNHGGGGNNSGPNAAASAGGYYQNYQGYSGYQQ
GGYQQYNPDAGYQQQYNPQGGYQQYNPQGGYQQQFNPQGGRGNYKNFNYNNNLQGYQAGFQP
QSQGMSLNDFQKQQKQAAPKPKKTLKLVSSSGIKLANATKKVGTKPAESDKKEEEKSAETKE
PTKEPTKVEEPVKKEEKPVQTEEKTEEKSELPKVEDLKISESTHNTNNANVTSADALIKEQE
EEVDDEVVND**HHHHHH**

Sup35NM chimeras

MKLLKVAAIAAIVFSGSALAGVVPQYGGGGNHGGGGNNSGPNAAAS**XXXXXXXXXXXXXXXX**
XXXXXAGGYYQNYQGYSGYQQGGYQQYNPDAGYQQQYNPQGGYQQYNPQGGYQQQFNPQGGR
GNYKNFNYNNNLQGYQAGFQPQSQGMSLNDFQKQQKQAAPKPKKTLKLVSSSGIKLANATKK
VGTKPAESDKKEEEKSAETKEPTKEPTKVEEPVKKEEKPVQTEEKTEEKSELPKVEDLKISE
STHNTNNANVTSADALIKEQEEEVDDEVVND**HHHHHH**
