# Supplementary figures and images for: Exogenous prion-like proteins and their potential to trigger cognitive dysfunction
Source: Mol Syst Biol. 2025 May 27;21(8):1004–29. doi: 10.1038/s44320-025-00114-4 (PMC12322145; doi:10.1038/s44320-025-00114-4)

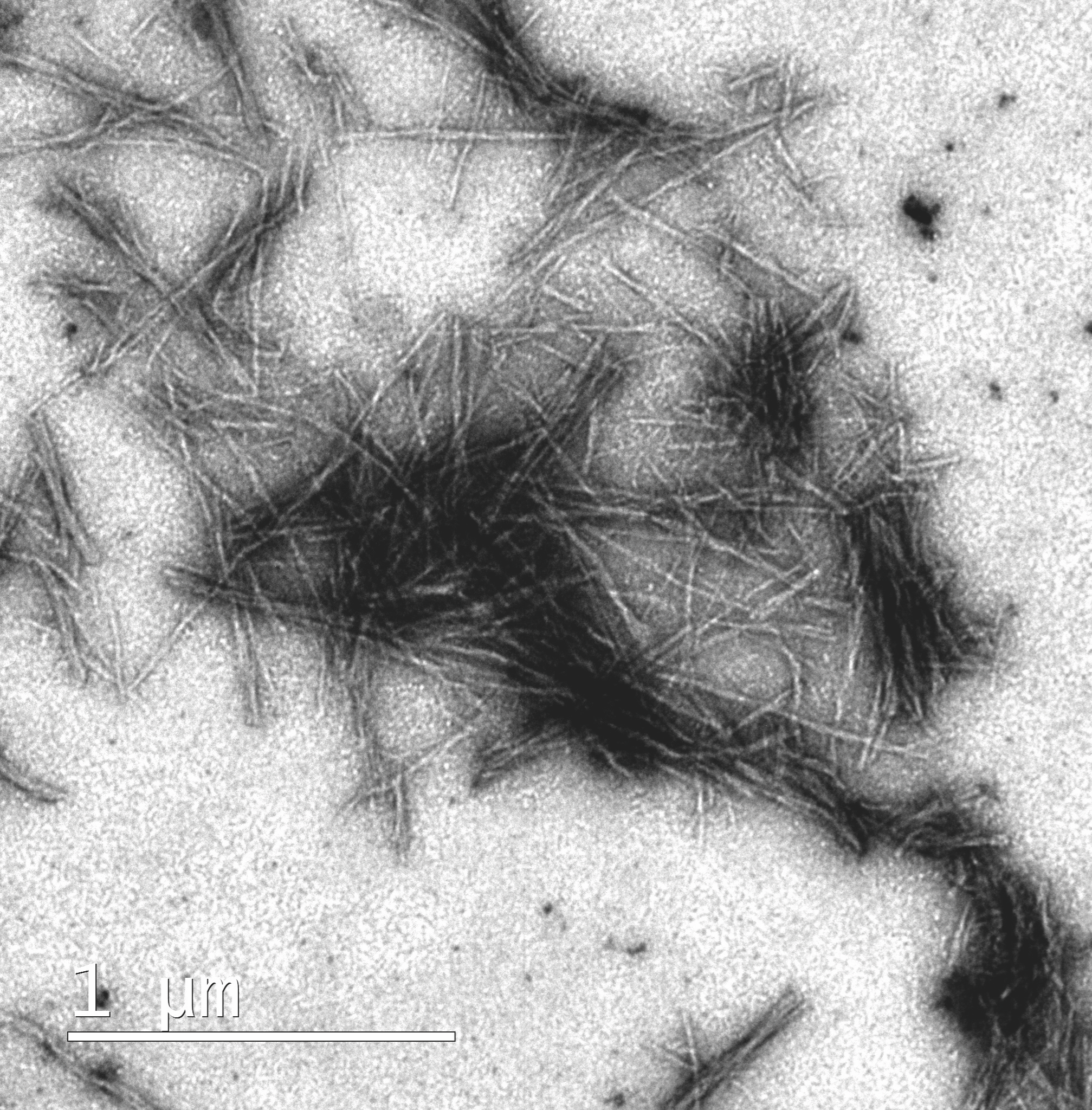

Supplement: Supplementary file 10 — Source data Fig. 2 [file 44320_2025_114_MOESM10_ESM.zip › Figure 2/2A/TEMS_peptides/RI5_4000X.tif]

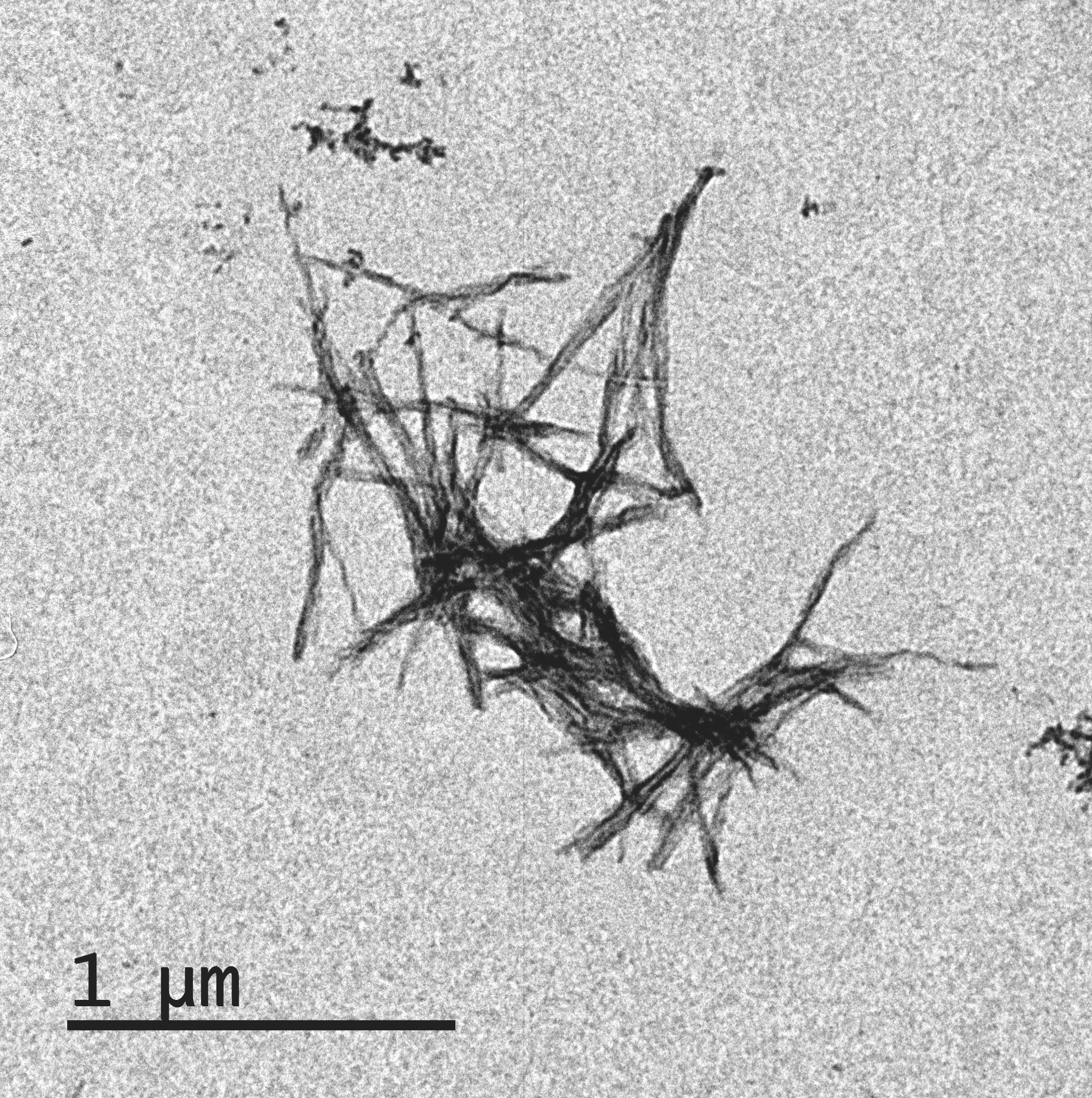

Supplement: Supplementary file 10 — Source data Fig. 2 [file 44320_2025_114_MOESM10_ESM.zip › Figure 2/2A/TEMS_peptides/HP2_4000x.tif]

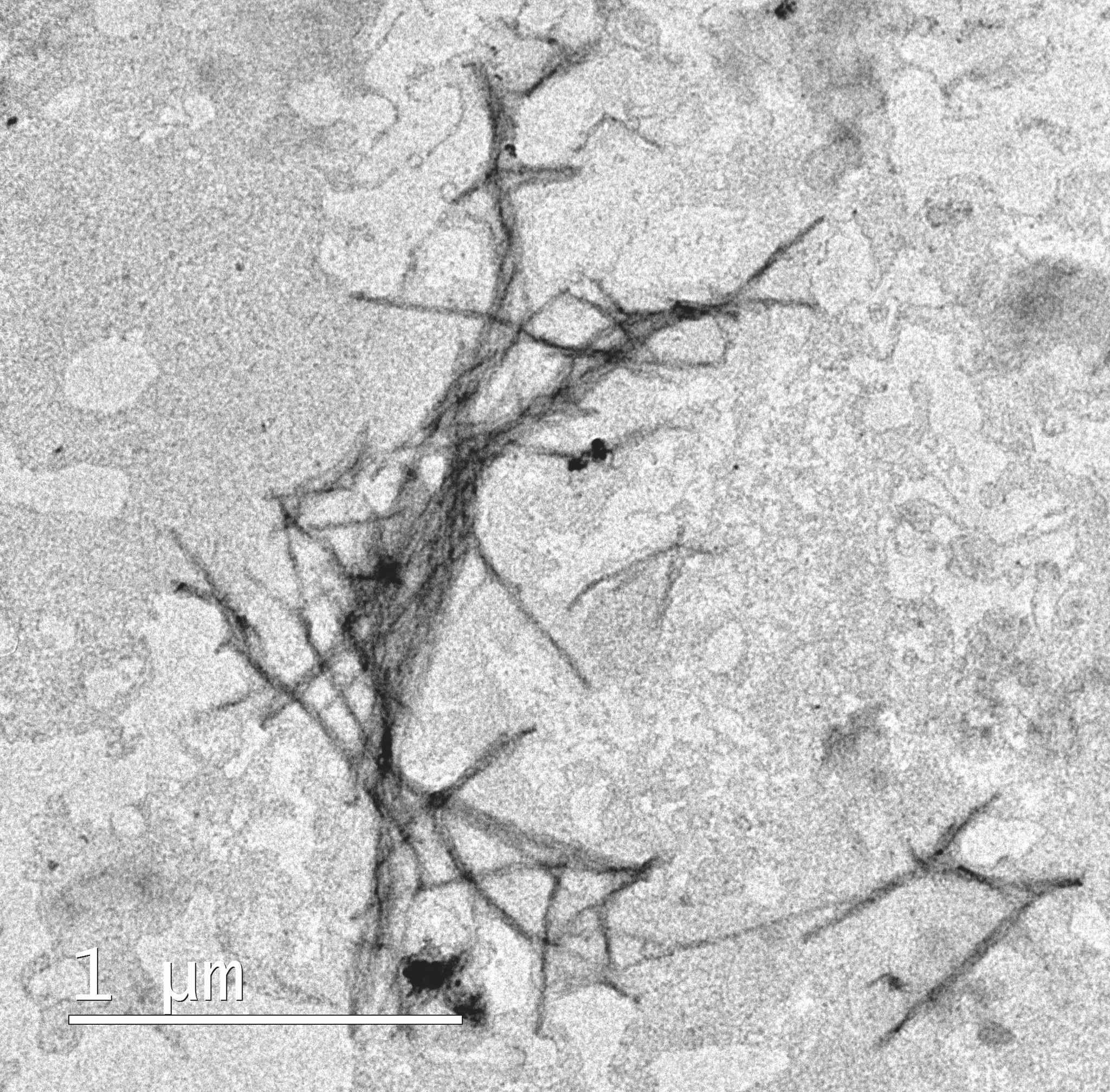

Supplement: Supplementary file 10 — Source data Fig. 2 [file 44320_2025_114_MOESM10_ESM.zip › Figure 2/2A/TEMS_peptides/CC3_4000X.tif]

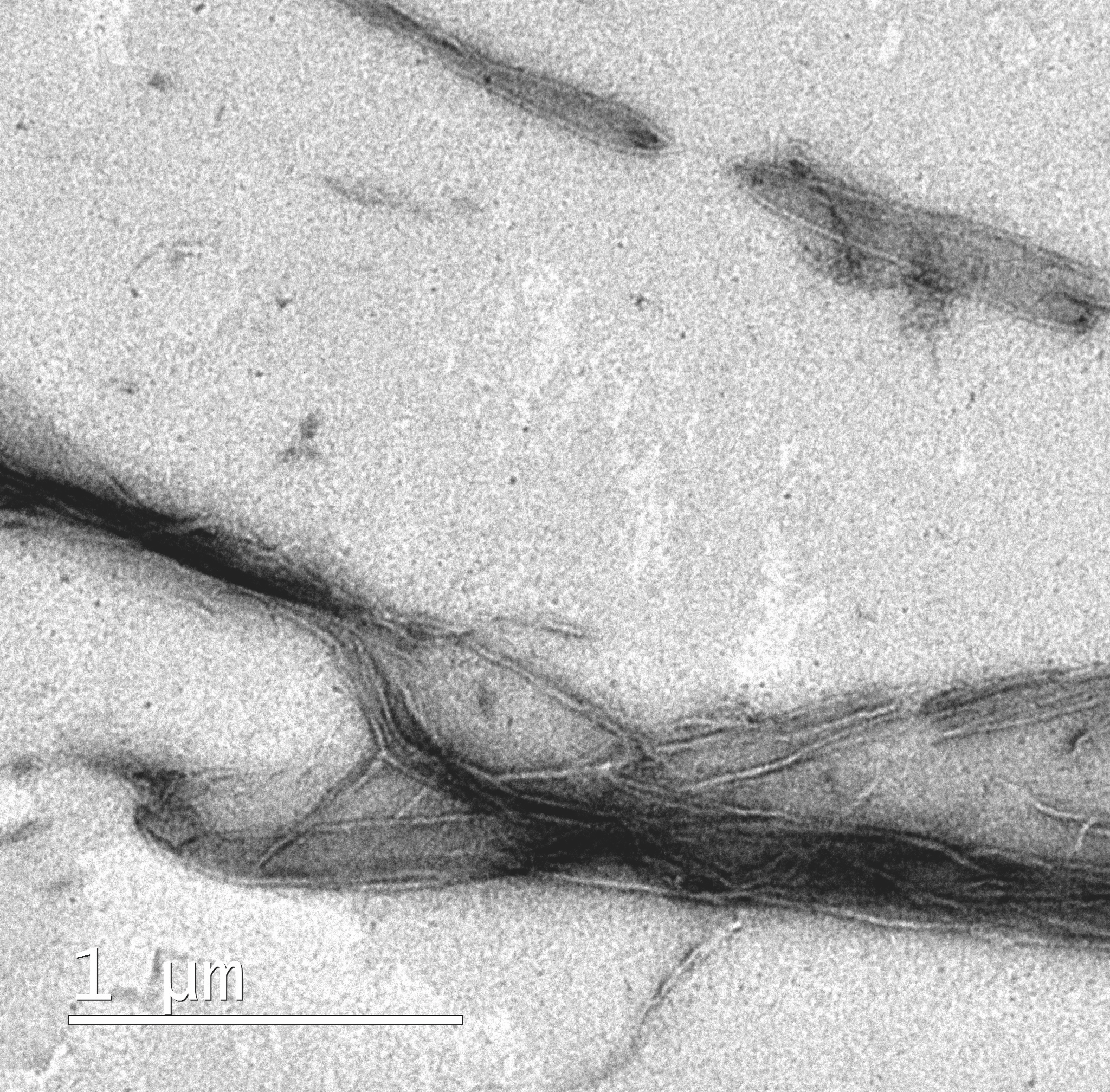

Supplement: Supplementary file 10 — Source data Fig. 2 [file 44320_2025_114_MOESM10_ESM.zip › Figure 2/2A/TEMS_peptides/RI6_4000X.tif]

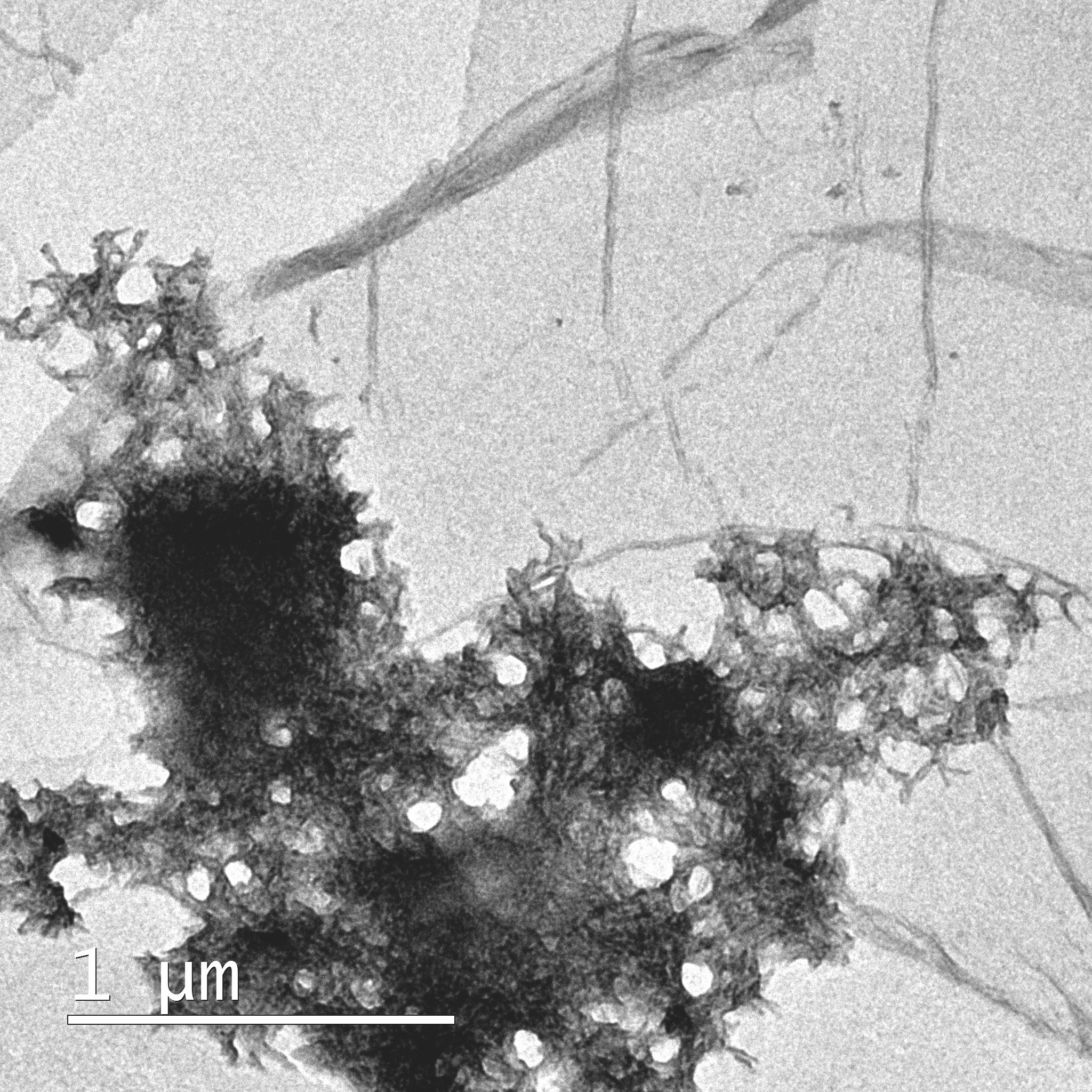

Supplement: Supplementary file 10 — Source data Fig. 2 [file 44320_2025_114_MOESM10_ESM.zip › Figure 2/2A/TEMS_peptides/HP1_4000X.tif]

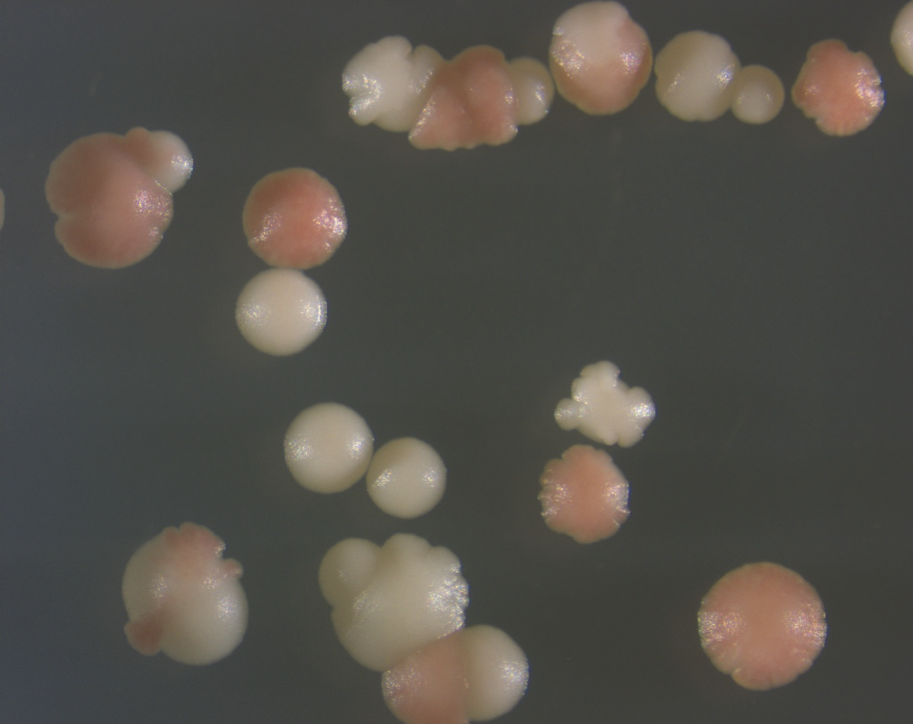

Supplement: Supplementary file 12 — Source data Fig. 4 [file 44320_2025_114_MOESM12_ESM.zip › Figure 4/4C/RI6.tiff]

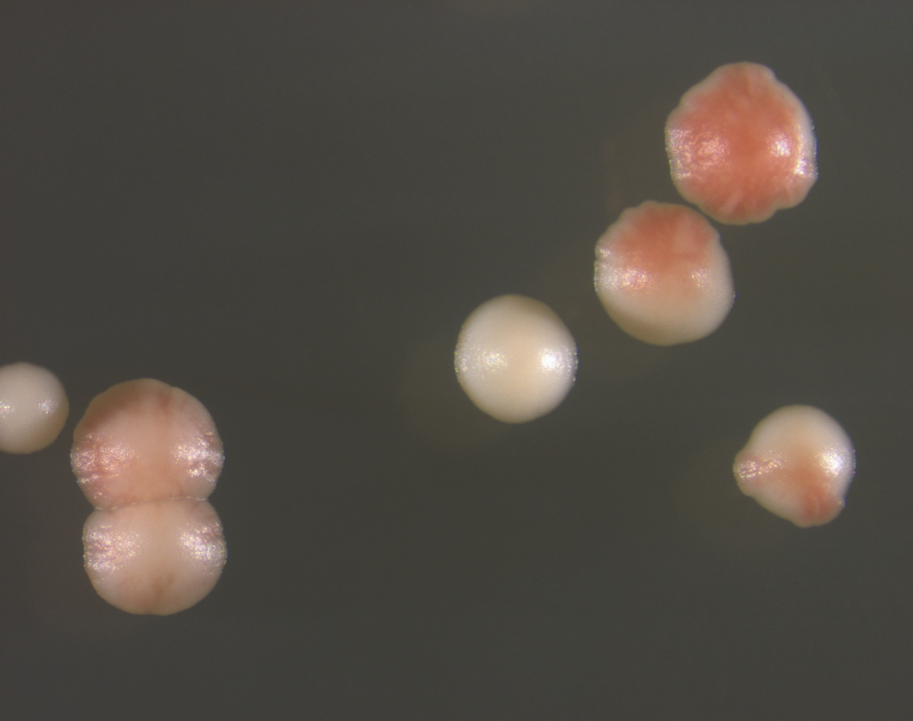

Supplement: Supplementary file 12 — Source data Fig. 4 [file 44320_2025_114_MOESM12_ESM.zip › Figure 4/4C/HA10.tiff]

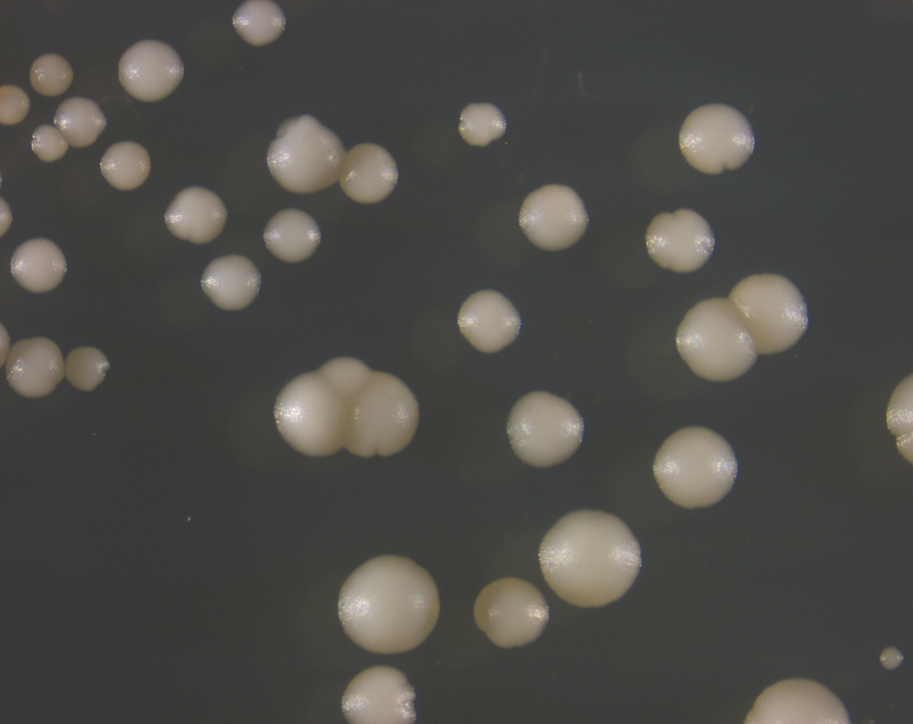

Supplement: Supplementary file 12 — Source data Fig. 4 [file 44320_2025_114_MOESM12_ESM.zip › Figure 4/4C/HP1.tiff]

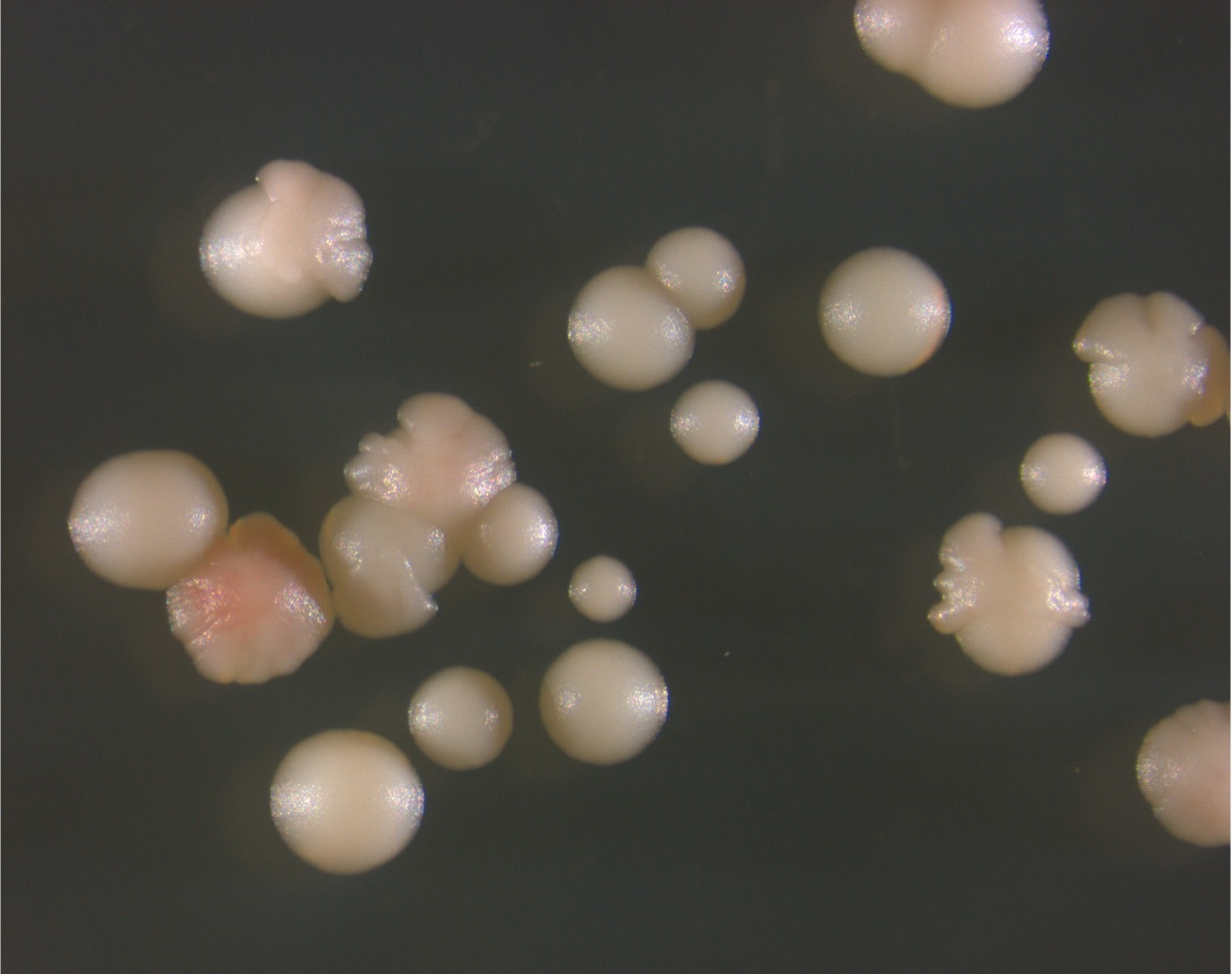

Supplement: Supplementary file 12 — Source data Fig. 4 [file 44320_2025_114_MOESM12_ESM.zip › Figure 4/4C/P9.jpg]

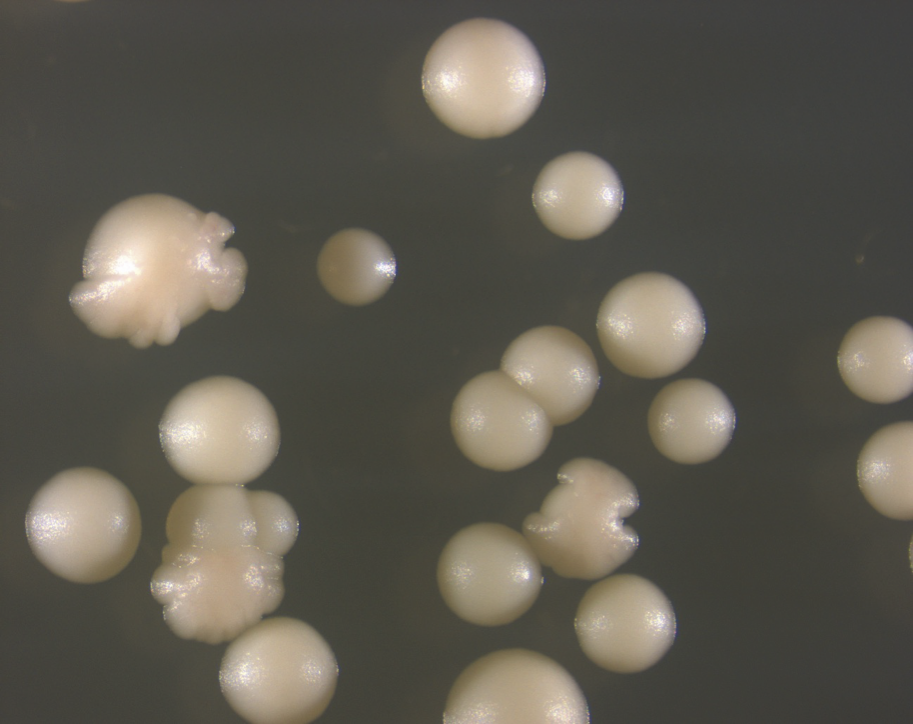

Supplement: Supplementary file 12 — Source data Fig. 4 [file 44320_2025_114_MOESM12_ESM.zip › Figure 4/4C/SA7.tiff]

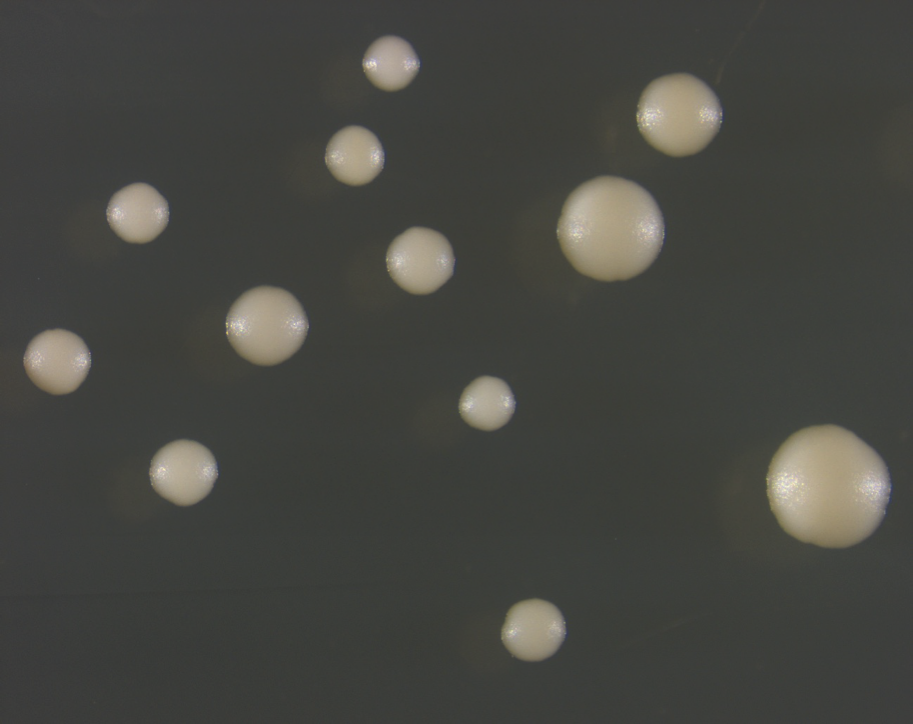

Supplement: Supplementary file 12 — Source data Fig. 4 [file 44320_2025_114_MOESM12_ESM.zip › Figure 4/4C/NM.tiff]

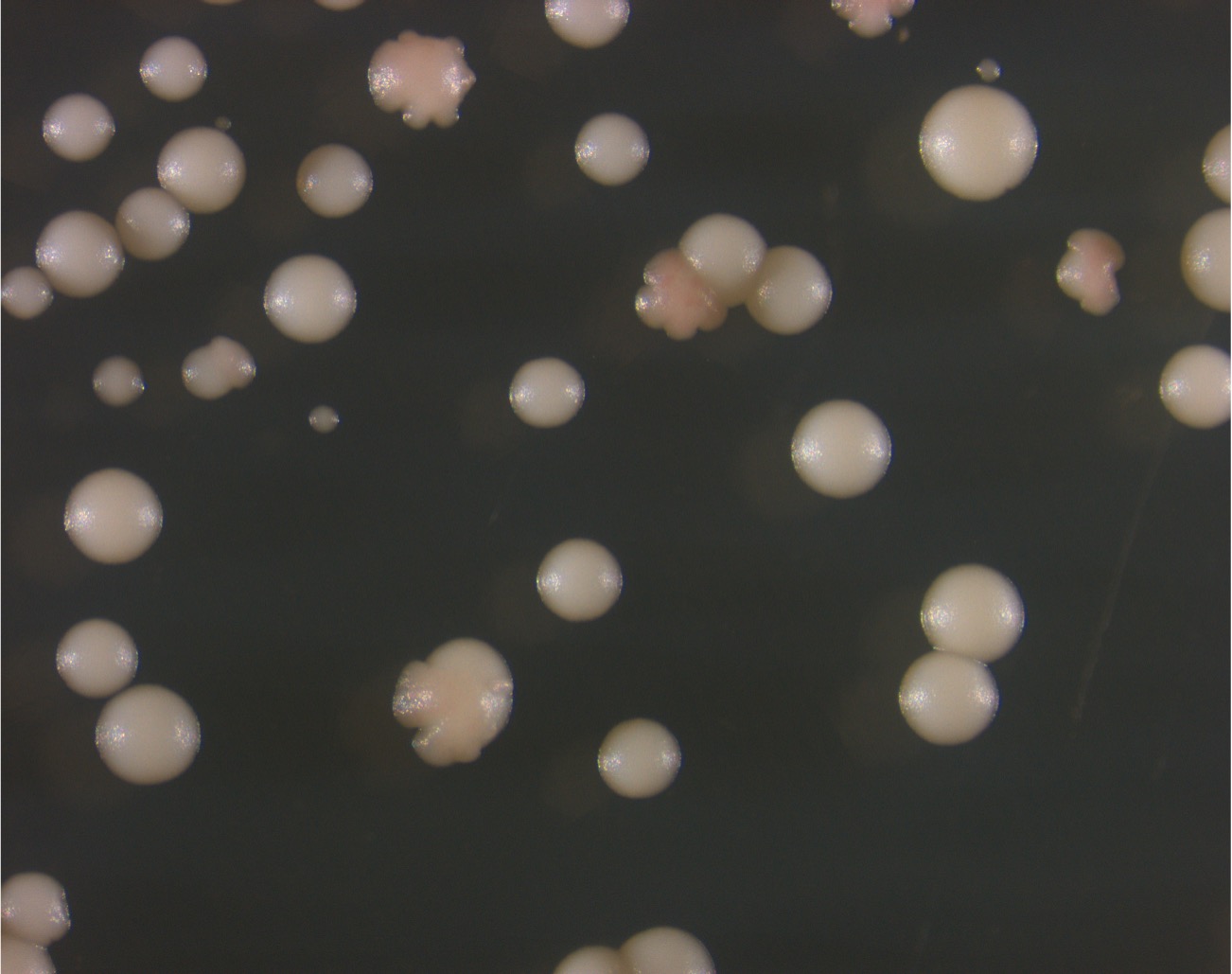

Supplement: Supplementary file 12 — Source data Fig. 4 [file 44320_2025_114_MOESM12_ESM.zip › Figure 4/4C/LD8.jpg]

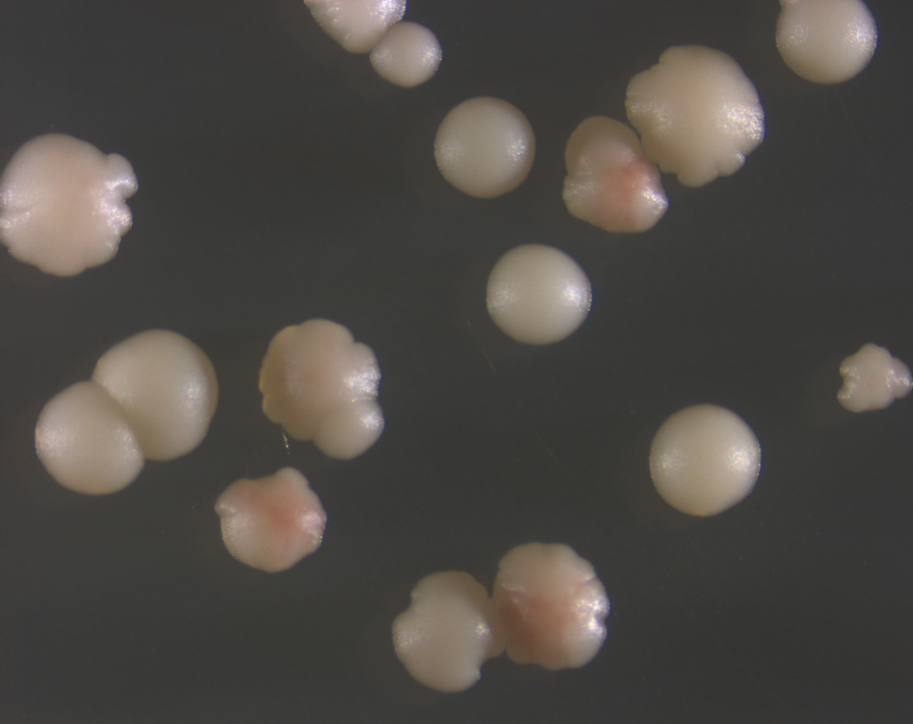

Supplement: Supplementary file 12 — Source data Fig. 4 [file 44320_2025_114_MOESM12_ESM.zip › Figure 4/4C/CC3.tiff]

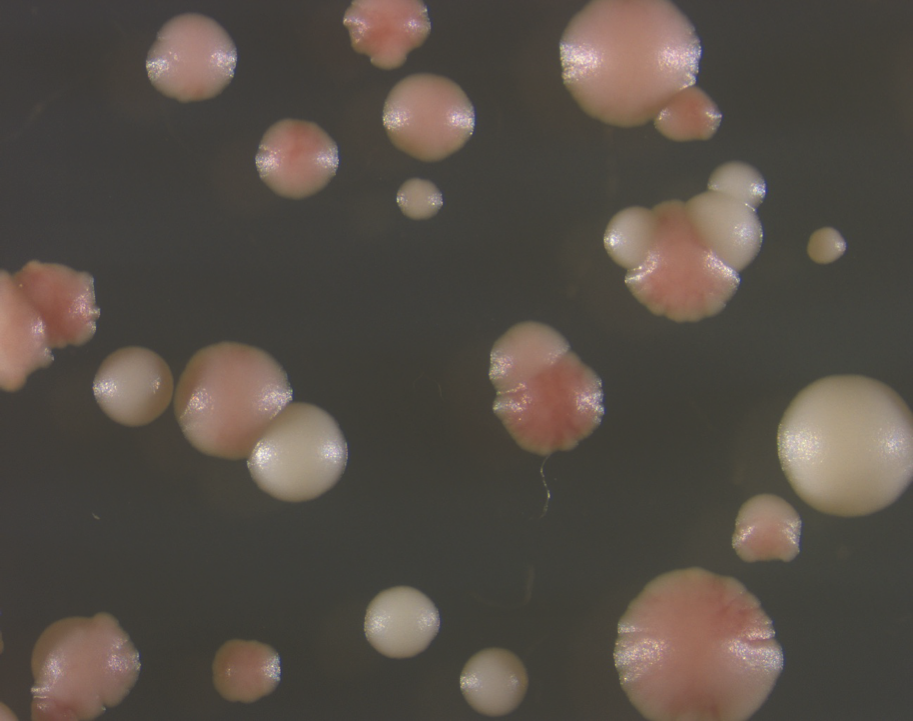

Supplement: Supplementary file 12 — Source data Fig. 4 [file 44320_2025_114_MOESM12_ESM.zip › Figure 4/4C/DeltaNM.tiff]

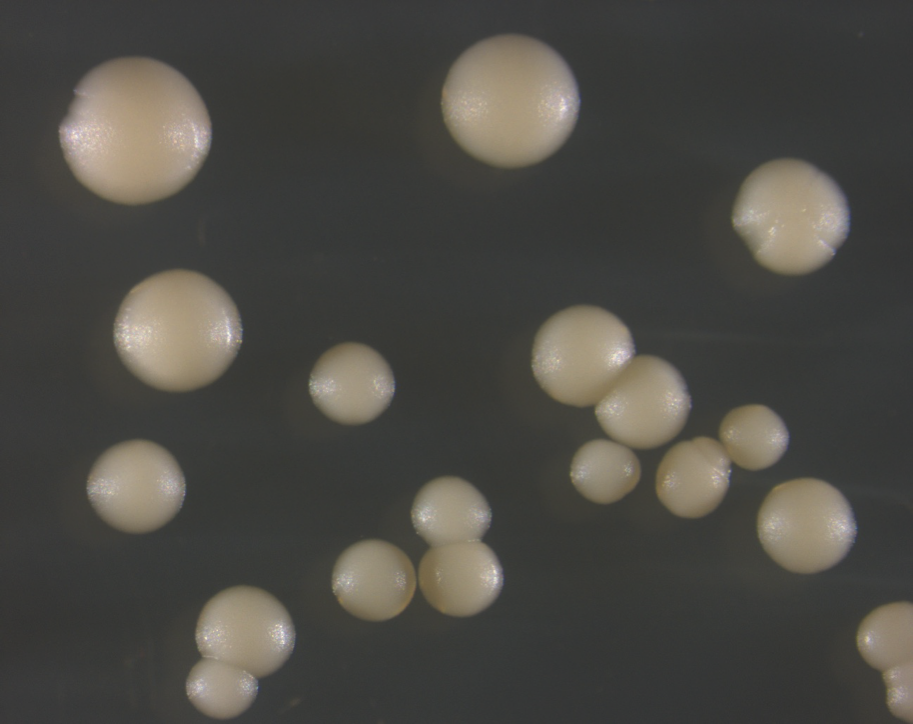

Supplement: Supplementary file 12 — Source data Fig. 4 [file 44320_2025_114_MOESM12_ESM.zip › Figure 4/4C/HP2.tiff]

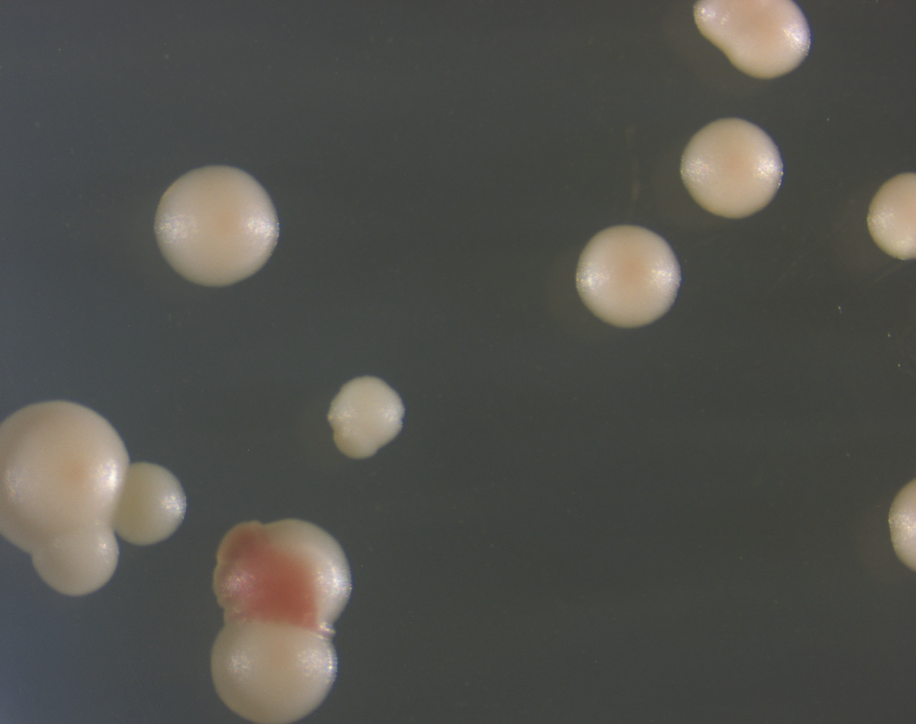

Supplement: Supplementary file 12 — Source data Fig. 4 [file 44320_2025_114_MOESM12_ESM.zip › Figure 4/4C/RI5.tiff]

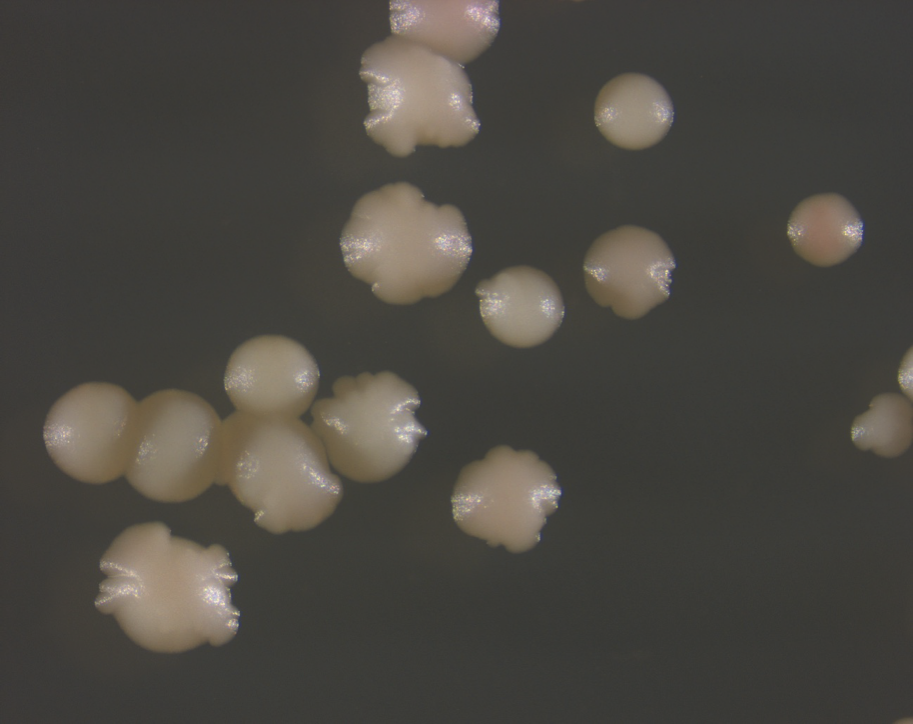

Supplement: Supplementary file 12 — Source data Fig. 4 [file 44320_2025_114_MOESM12_ESM.zip › Figure 4/4C/BH4.tiff]

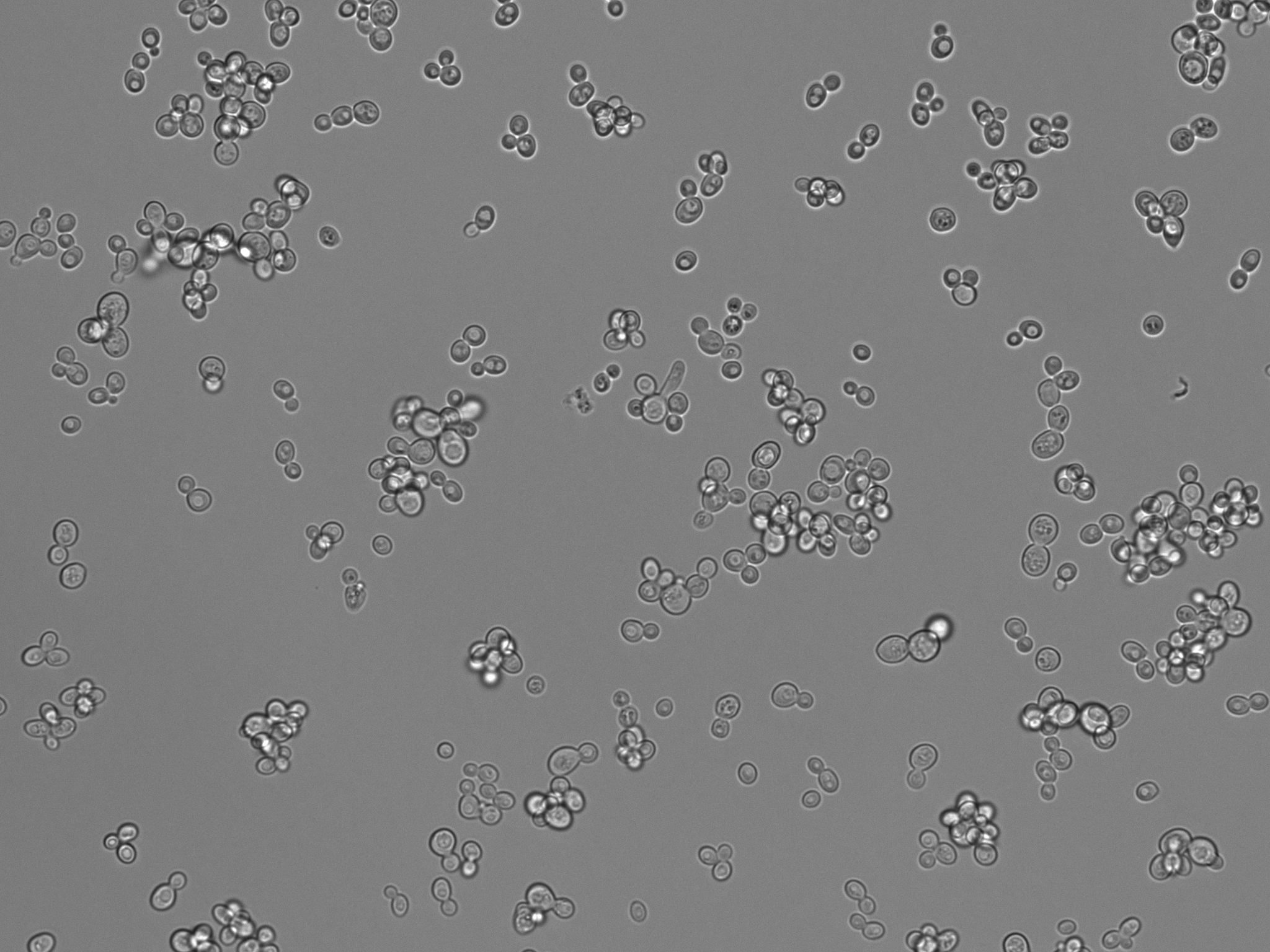

Supplement: Supplementary file 12 — Source data Fig. 4 [file 44320_2025_114_MOESM12_ESM.zip › Figure 4/4B/GFP yeast/Fig-LD8_0001_Trans.tif]

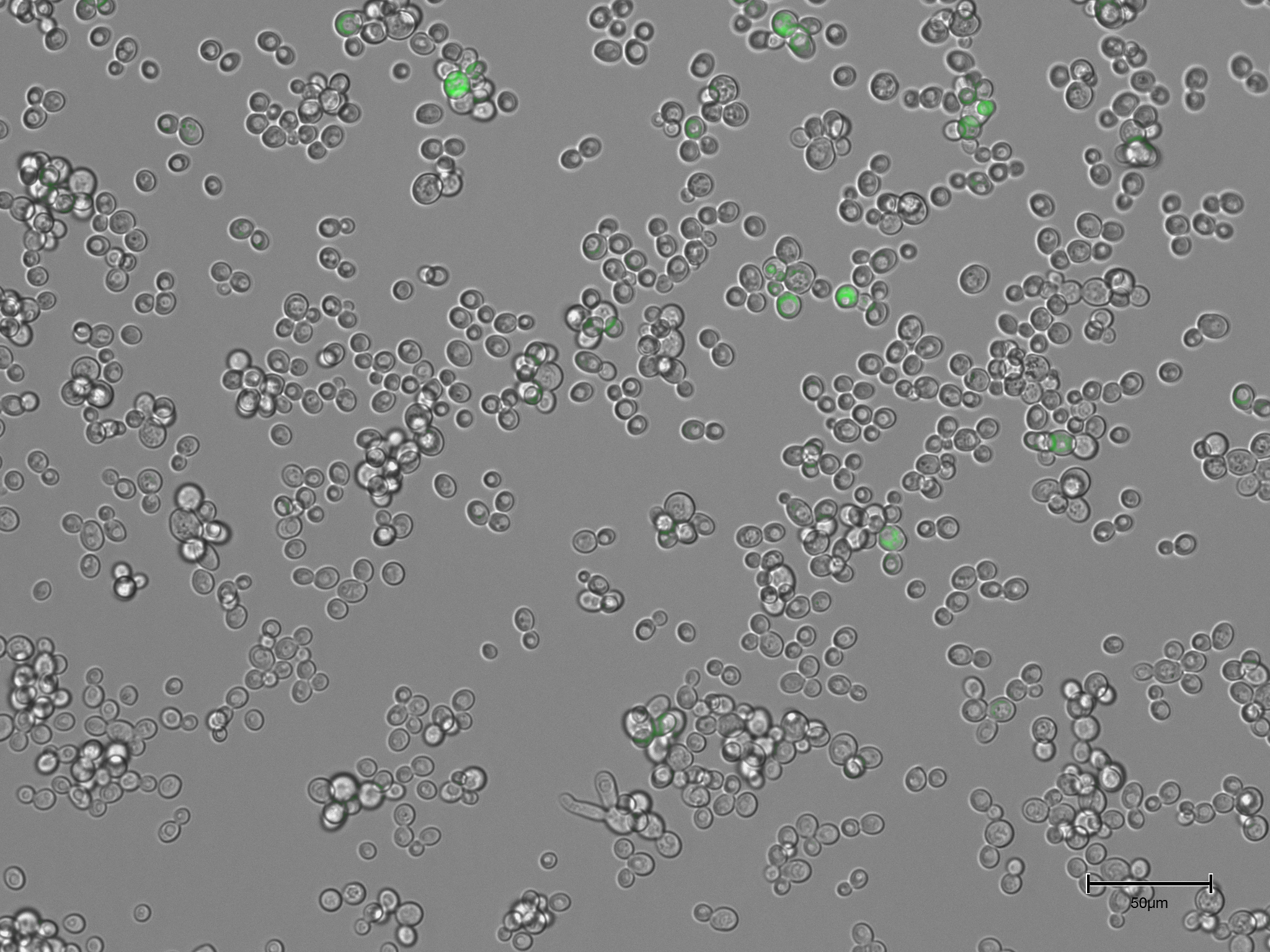

Supplement: Supplementary file 12 — Source data Fig. 4 [file 44320_2025_114_MOESM12_ESM.zip › Figure 4/4B/GFP yeast/Fig-deltaNM_0003.tif]

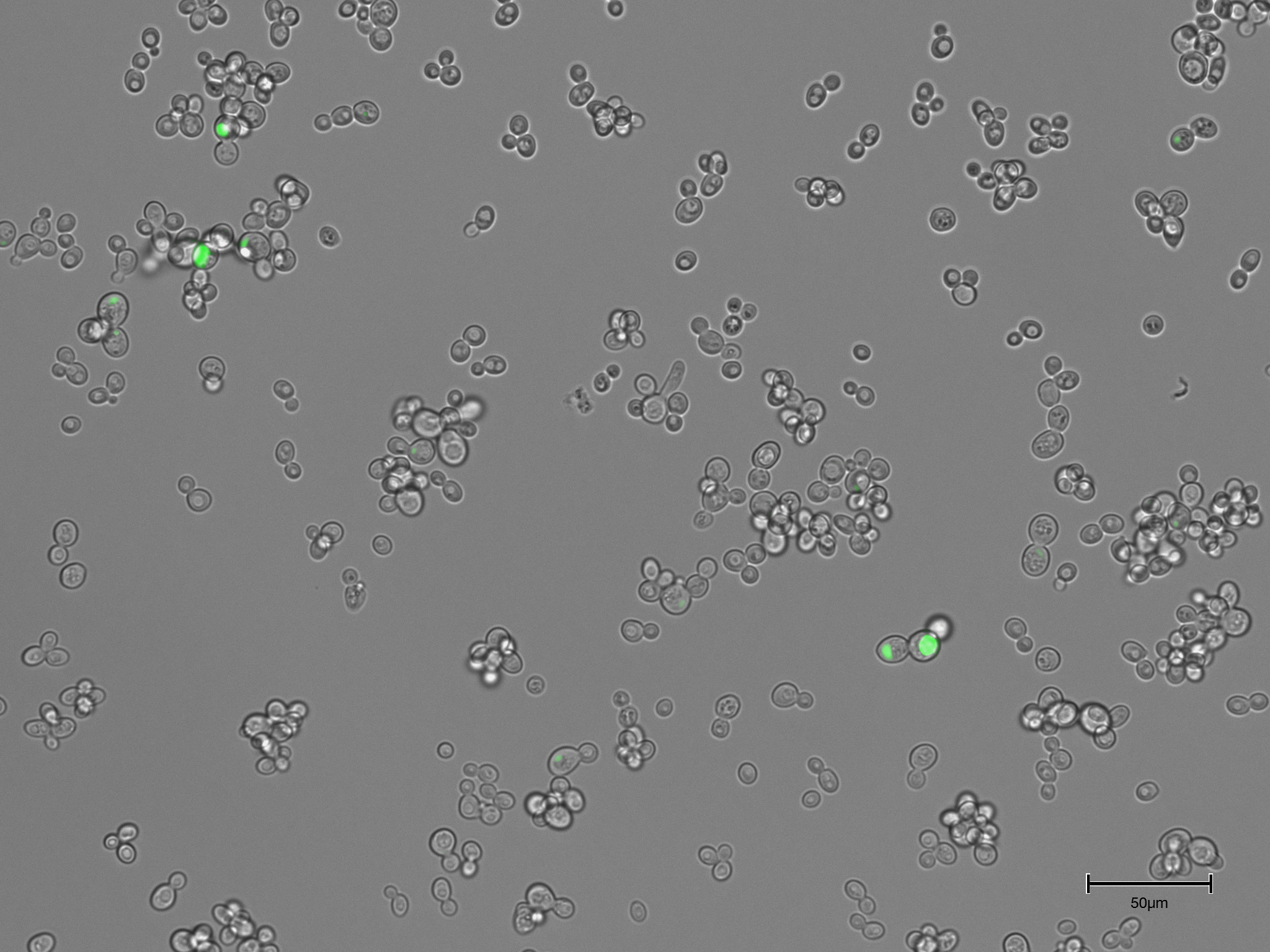

Supplement: Supplementary file 12 — Source data Fig. 4 [file 44320_2025_114_MOESM12_ESM.zip › Figure 4/4B/GFP yeast/Fig-LD8_0001.tif]

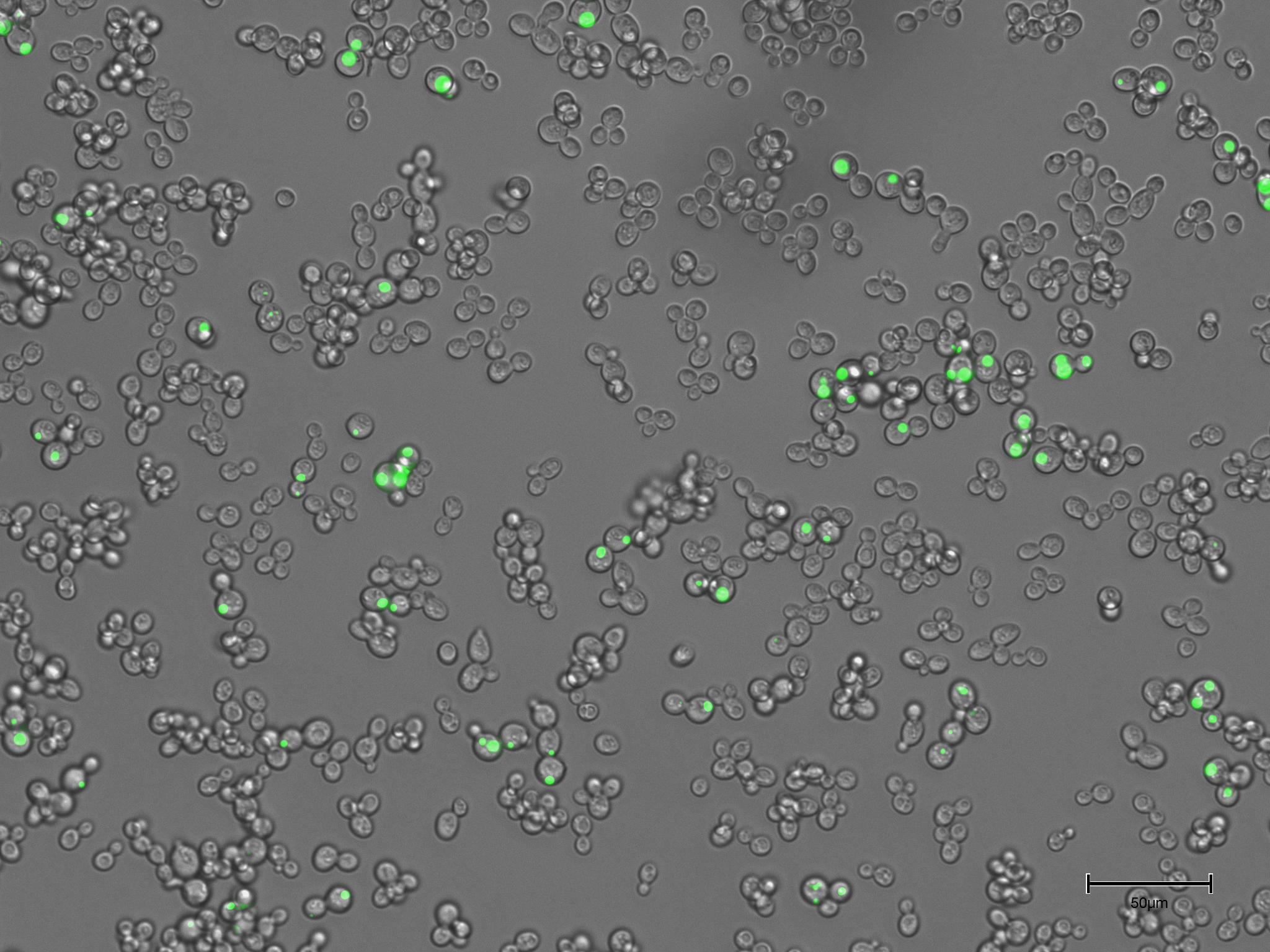

Supplement: Supplementary file 12 — Source data Fig. 4 [file 44320_2025_114_MOESM12_ESM.zip › Figure 4/4B/GFP yeast/Fig-NM_0004.tif]

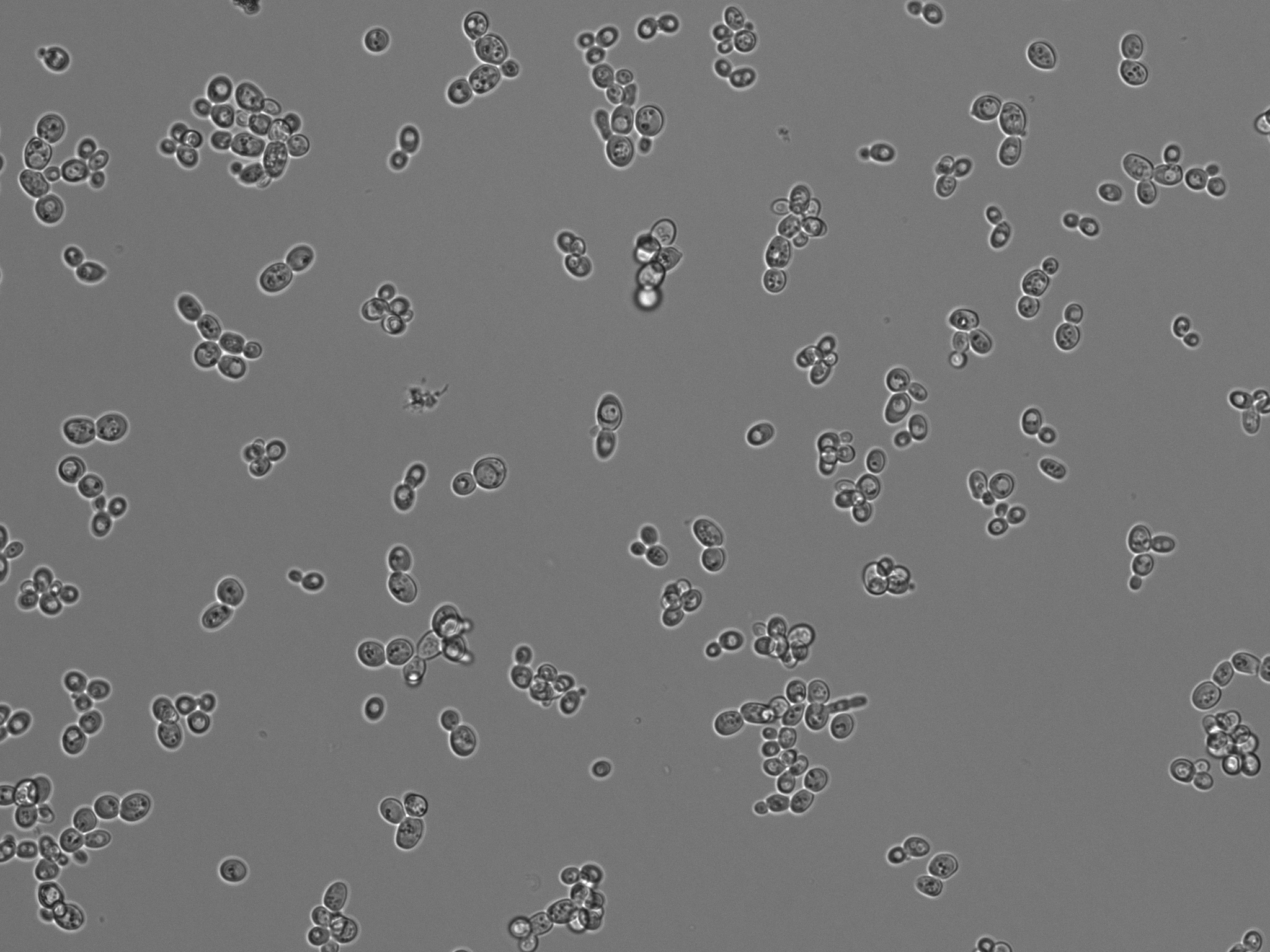

Supplement: Supplementary file 12 — Source data Fig. 4 [file 44320_2025_114_MOESM12_ESM.zip › Figure 4/4B/GFP yeast/Fig-HP2_0001_Trans.tif]

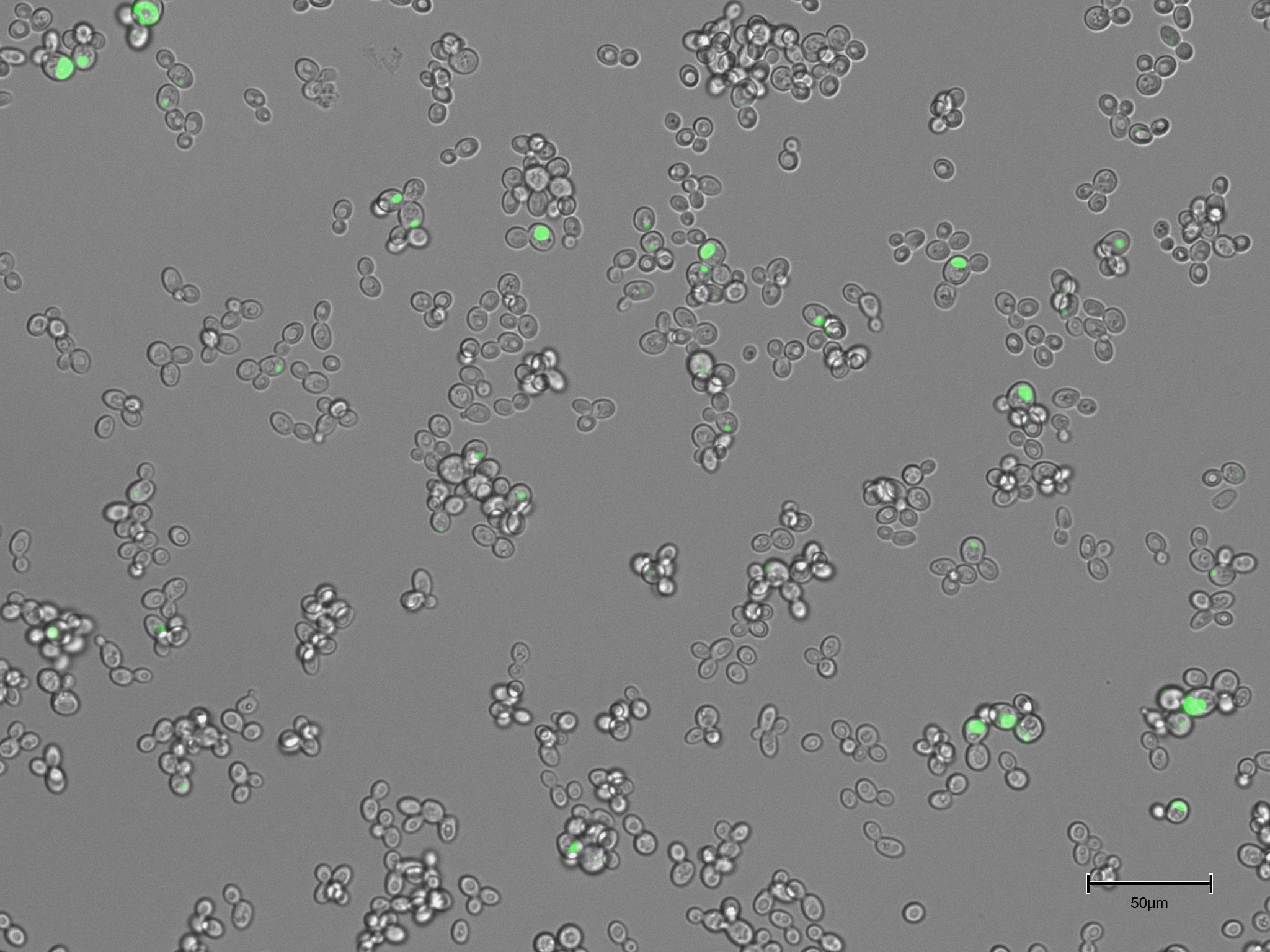

Supplement: Supplementary file 12 — Source data Fig. 4 [file 44320_2025_114_MOESM12_ESM.zip › Figure 4/4B/GFP yeast/Fig-P9_0003.tif]

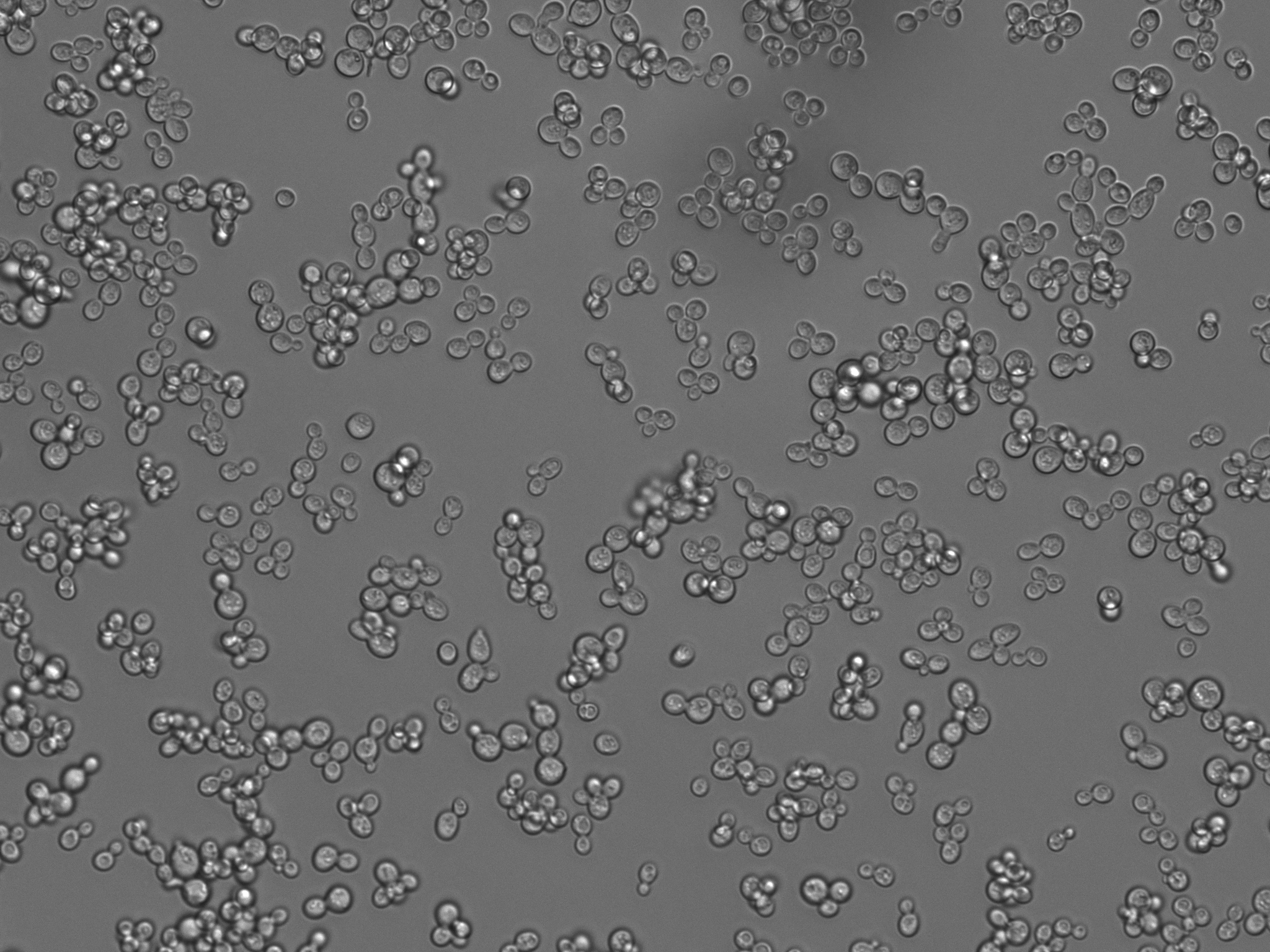

Supplement: Supplementary file 12 — Source data Fig. 4 [file 44320_2025_114_MOESM12_ESM.zip › Figure 4/4B/GFP yeast/Fig-NM_0004_Trans.tif]

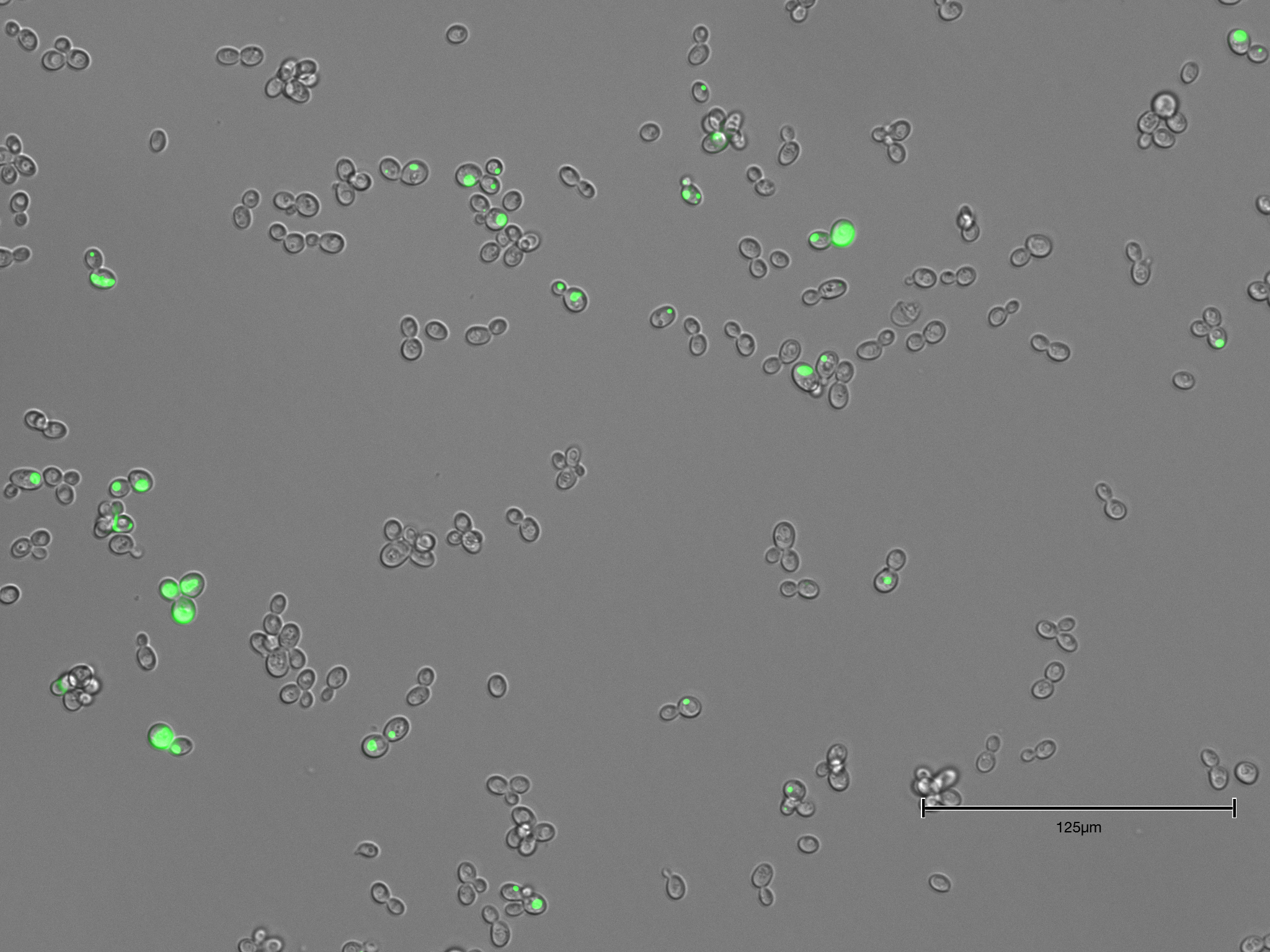

Supplement: Supplementary file 12 — Source data Fig. 4 [file 44320_2025_114_MOESM12_ESM.zip › Figure 4/4B/GFP yeast/Fig-CC3_0002.tif]

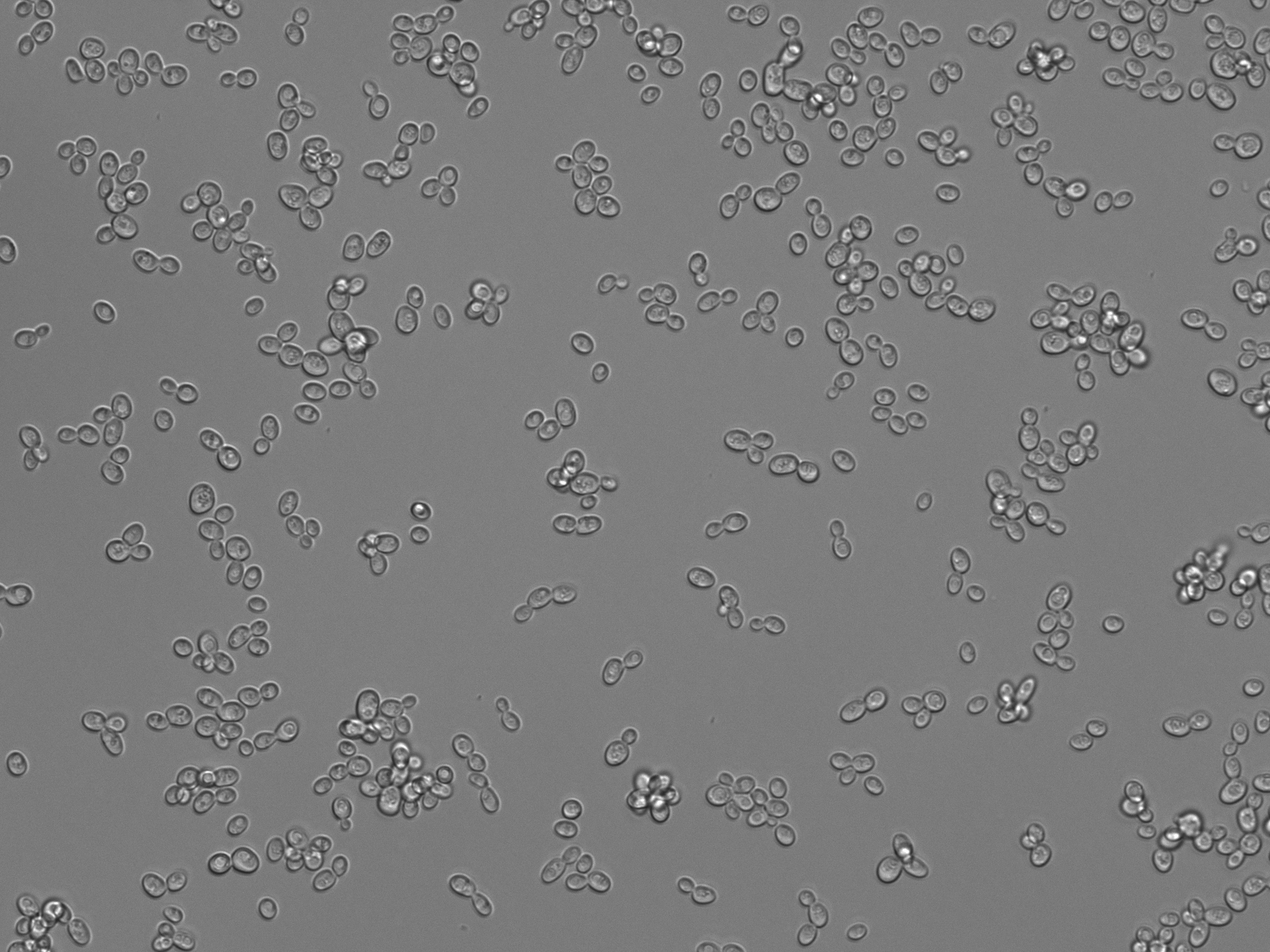

Supplement: Supplementary file 12 — Source data Fig. 4 [file 44320_2025_114_MOESM12_ESM.zip › Figure 4/4B/GFP yeast/Fig-RI6_0002_Trans.tif]

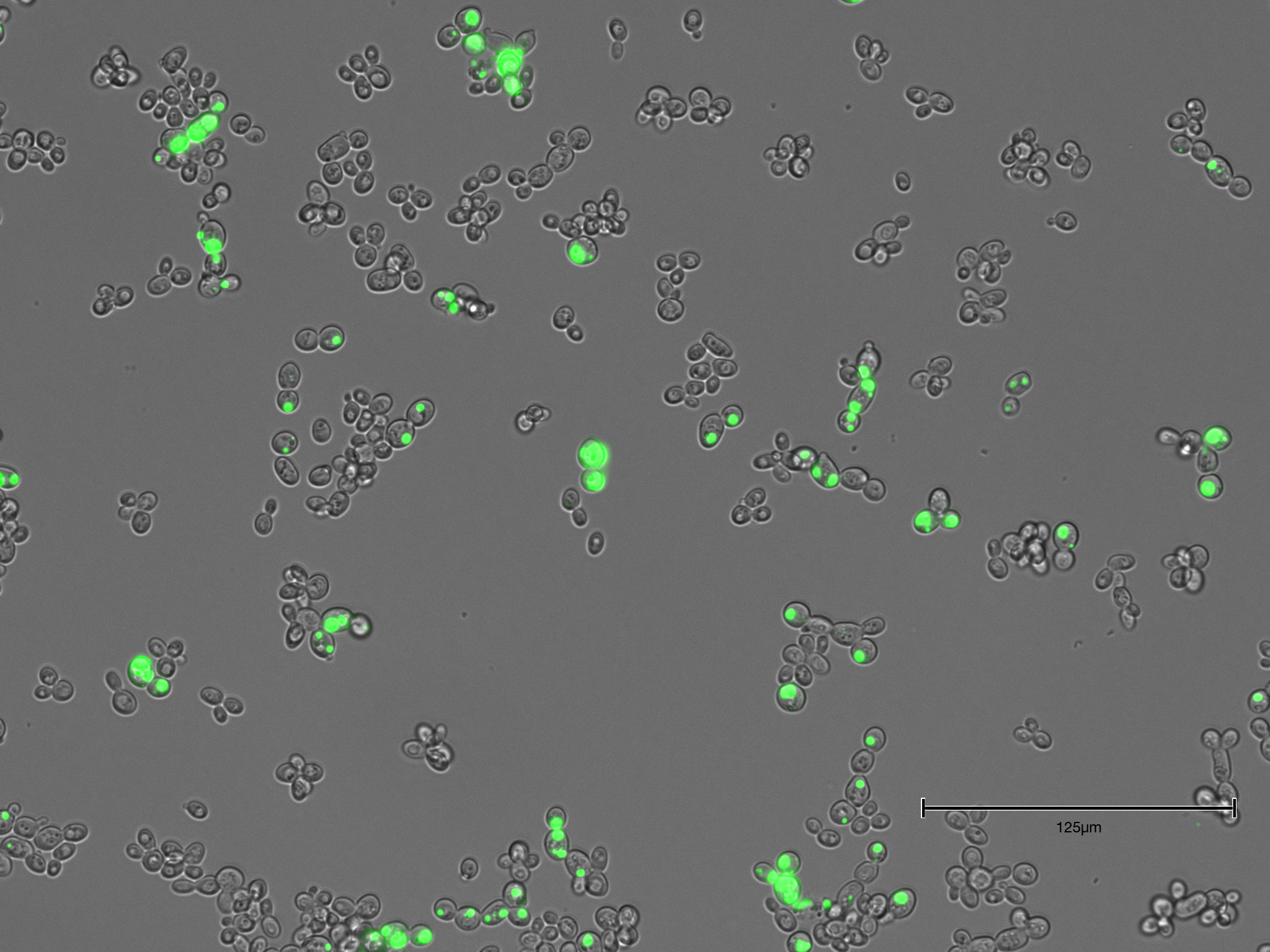

Supplement: Supplementary file 12 — Source data Fig. 4 [file 44320_2025_114_MOESM12_ESM.zip › Figure 4/4B/GFP yeast/Fig-HP1_0003.tif]

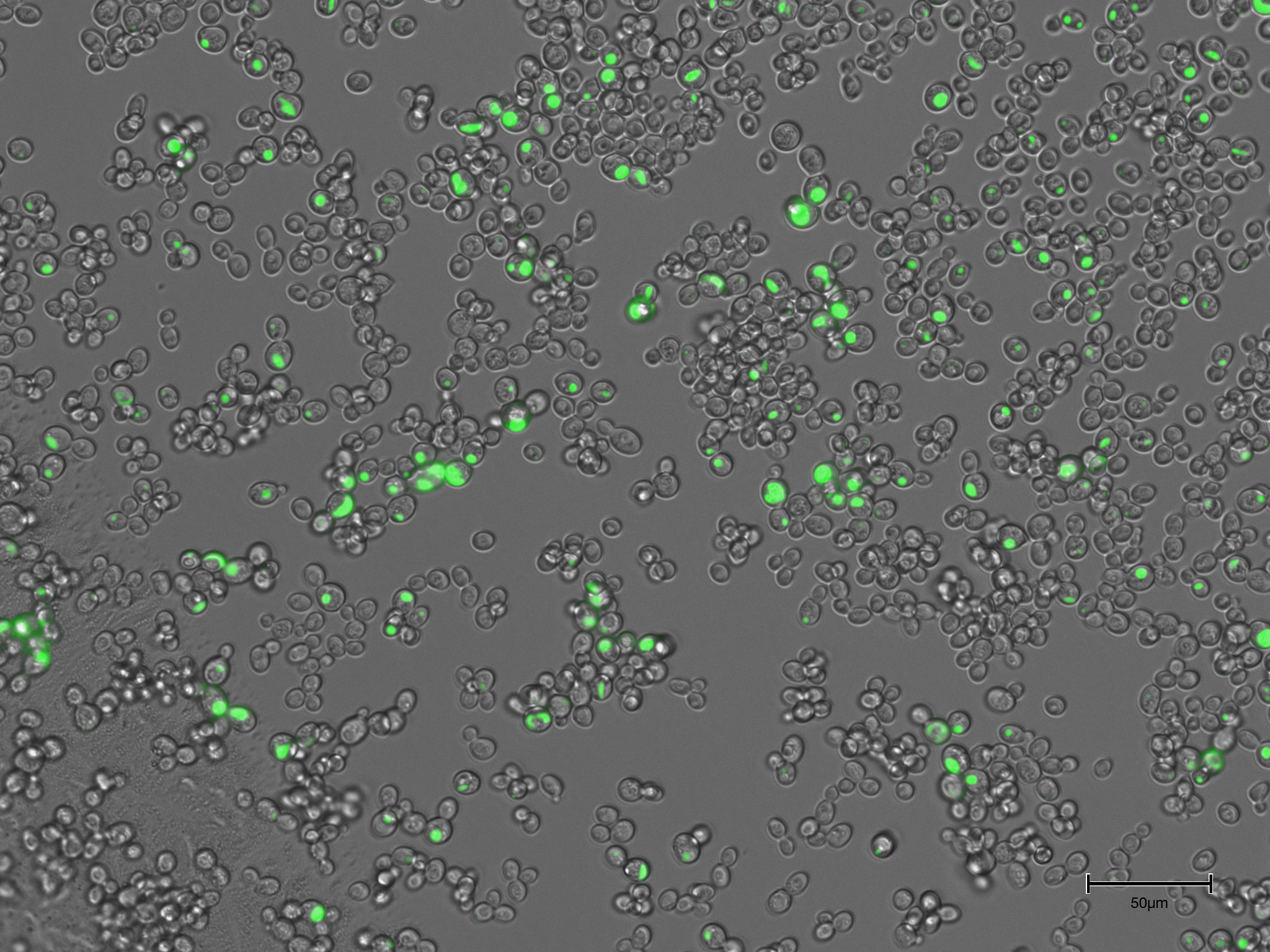

Supplement: Supplementary file 12 — Source data Fig. 4 [file 44320_2025_114_MOESM12_ESM.zip › Figure 4/4B/GFP yeast/Fig-RI5_0002.tif]

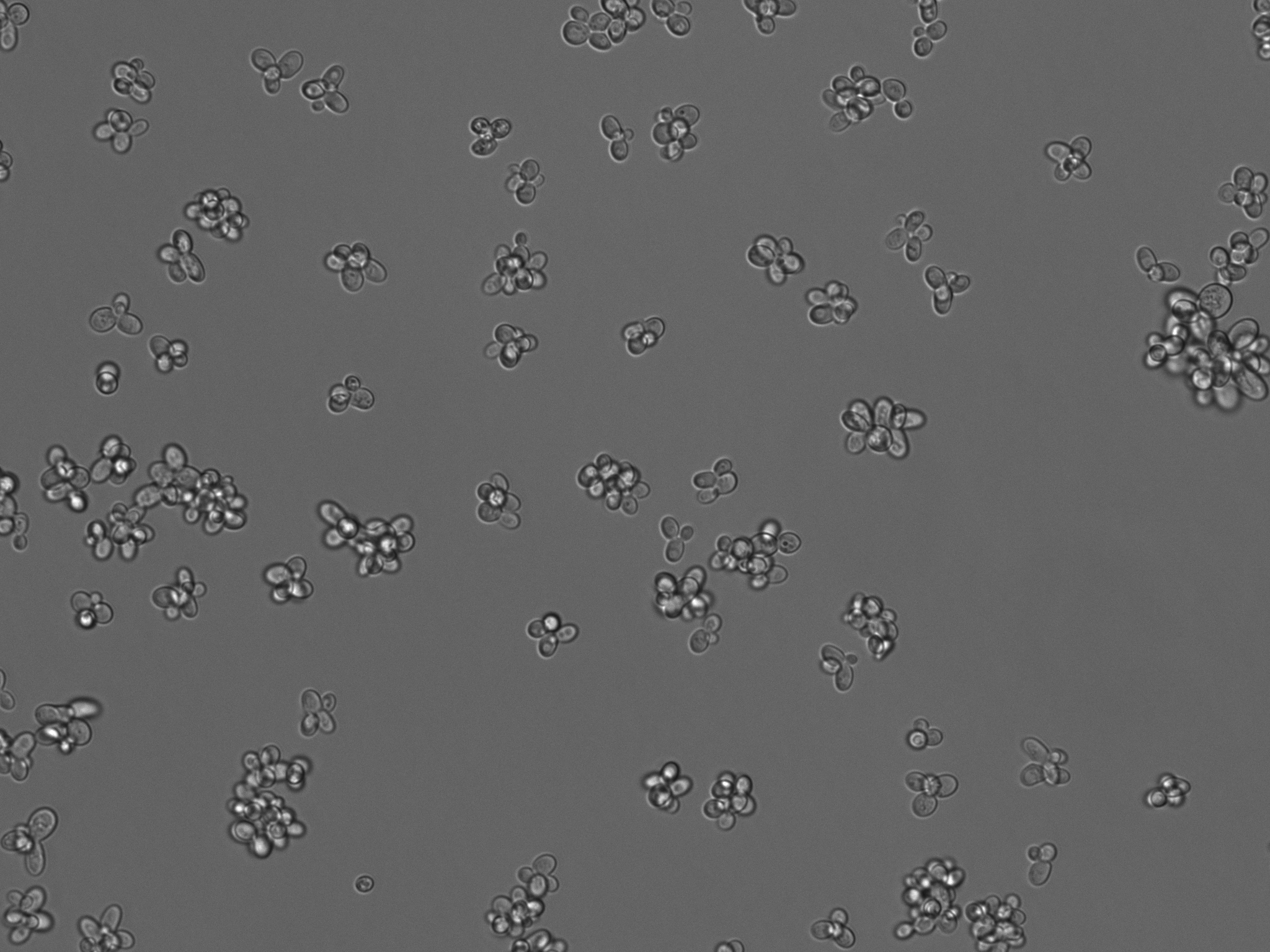

Supplement: Supplementary file 12 — Source data Fig. 4 [file 44320_2025_114_MOESM12_ESM.zip › Figure 4/4B/GFP yeast/Fig-HA10_0024_Trans.tif]

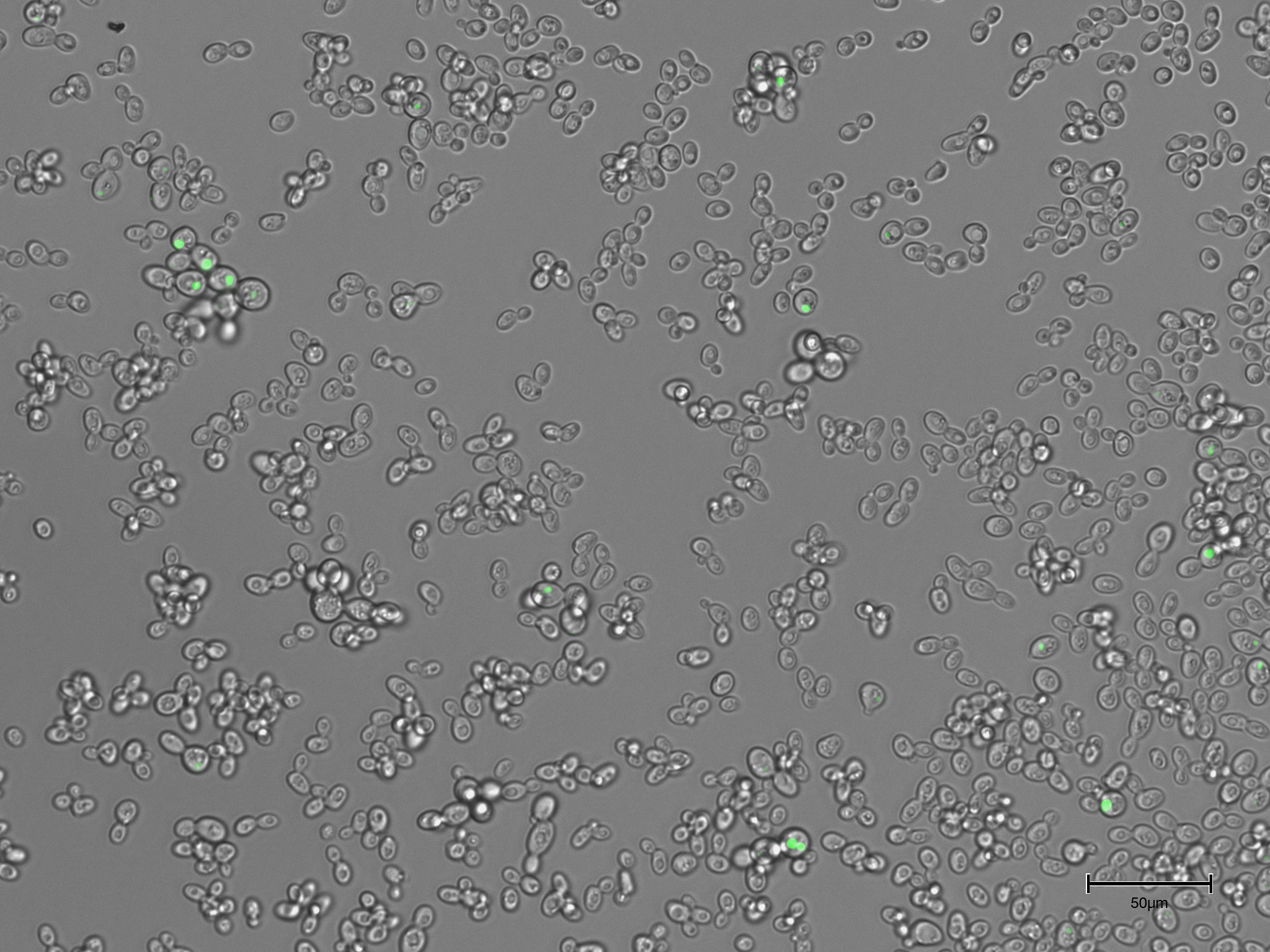

Supplement: Supplementary file 12 — Source data Fig. 4 [file 44320_2025_114_MOESM12_ESM.zip › Figure 4/4B/GFP yeast/Fig-SA7_0003.tif]

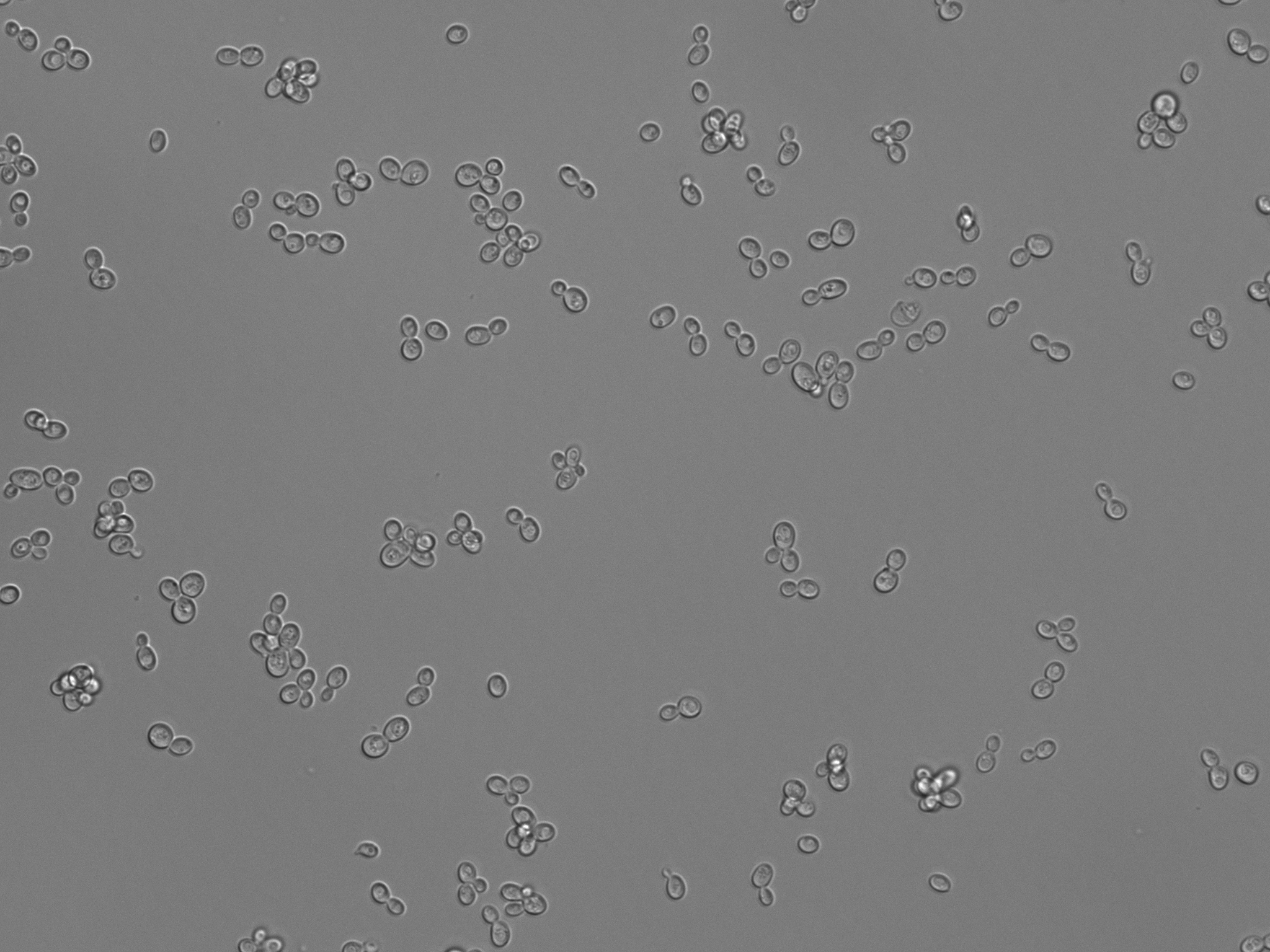

Supplement: Supplementary file 12 — Source data Fig. 4 [file 44320_2025_114_MOESM12_ESM.zip › Figure 4/4B/GFP yeast/Fig-CC3_0002_Trans.tif]

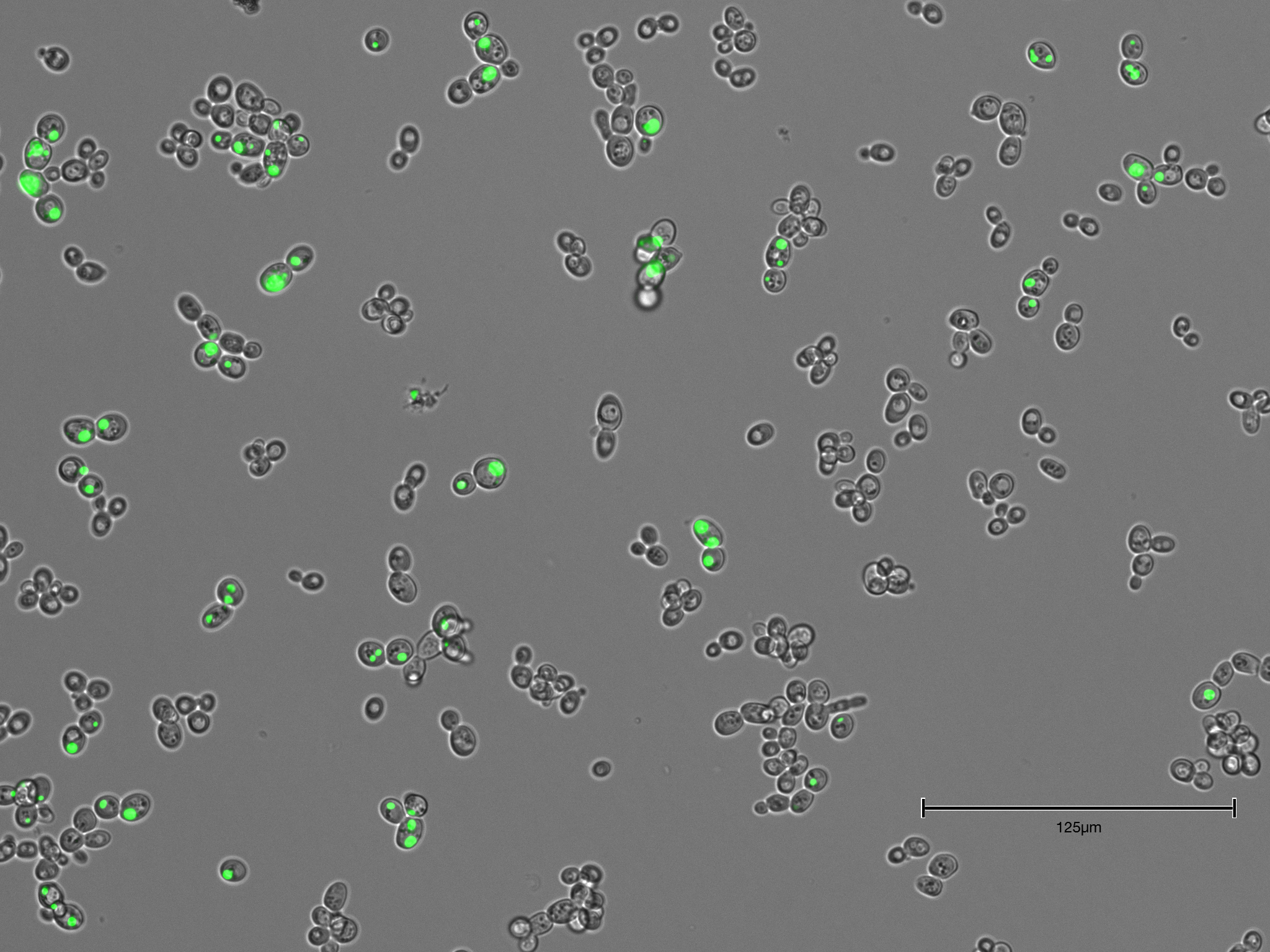

Supplement: Supplementary file 12 — Source data Fig. 4 [file 44320_2025_114_MOESM12_ESM.zip › Figure 4/4B/GFP yeast/Fig-HP2_0001.tif]

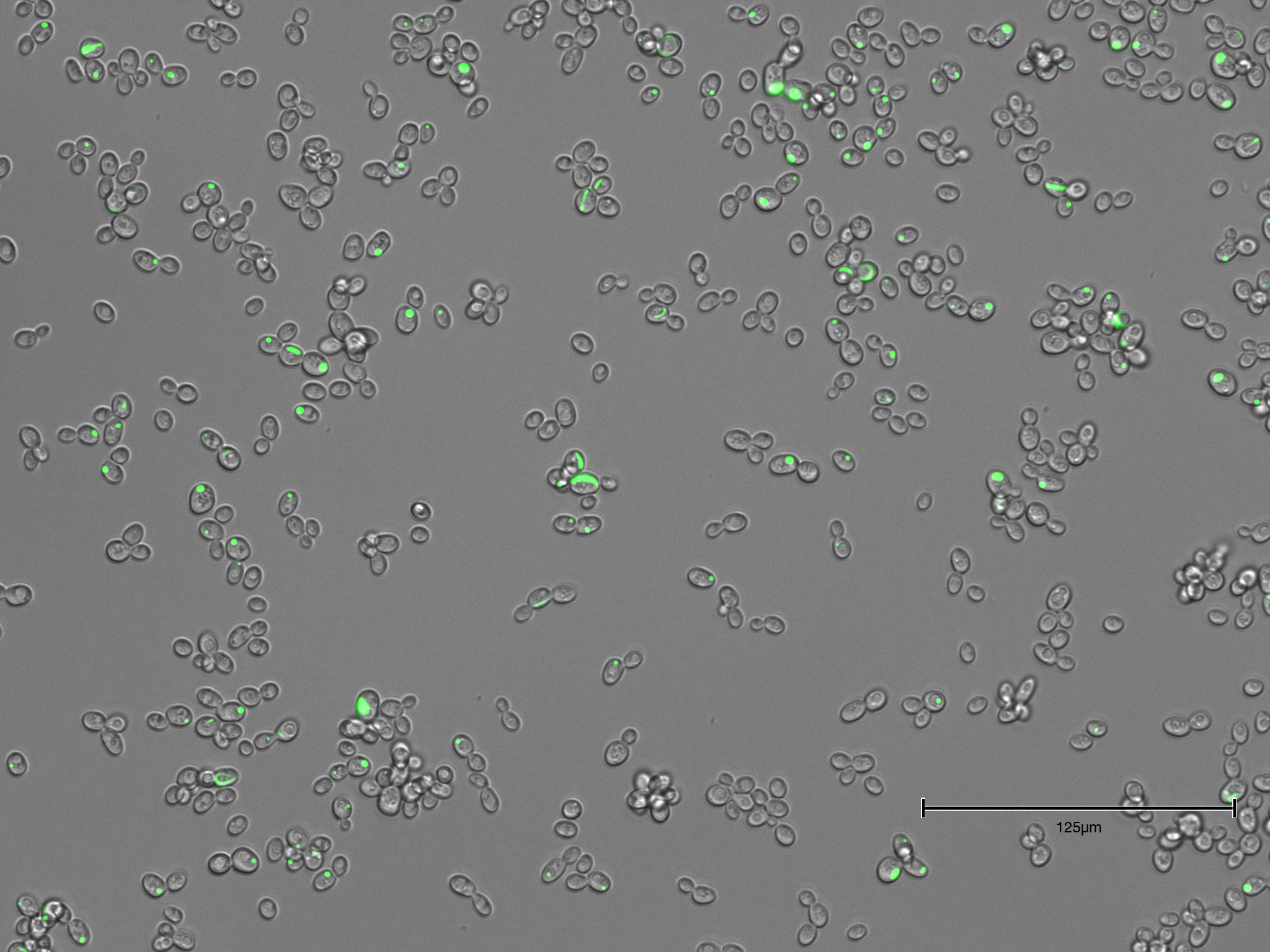

Supplement: Supplementary file 12 — Source data Fig. 4 [file 44320_2025_114_MOESM12_ESM.zip › Figure 4/4B/GFP yeast/Fig-RI6_0002.tif]

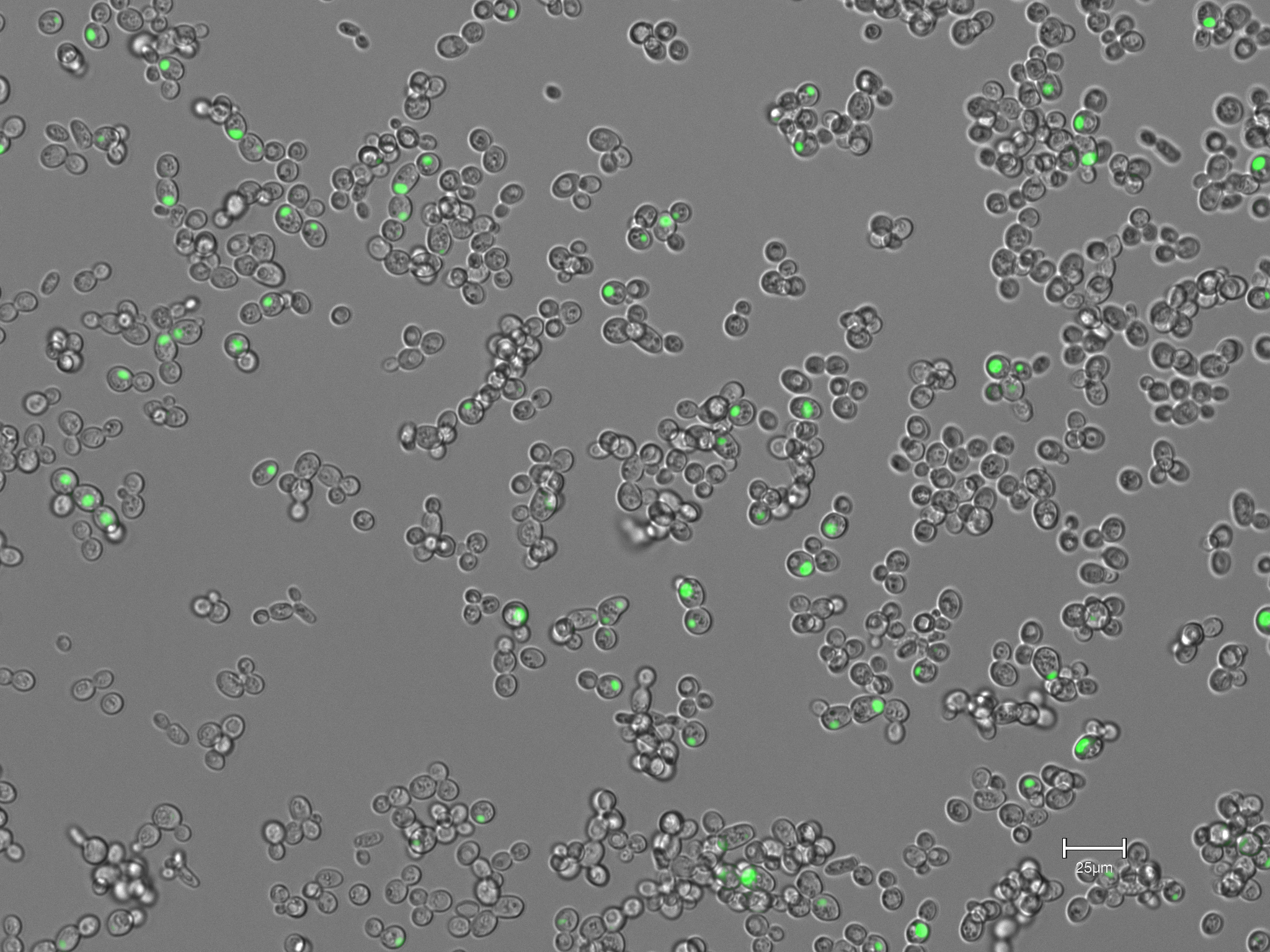

Supplement: Supplementary file 12 — Source data Fig. 4 [file 44320_2025_114_MOESM12_ESM.zip › Figure 4/4B/GFP yeast/Fig-BH4_0003.tif]

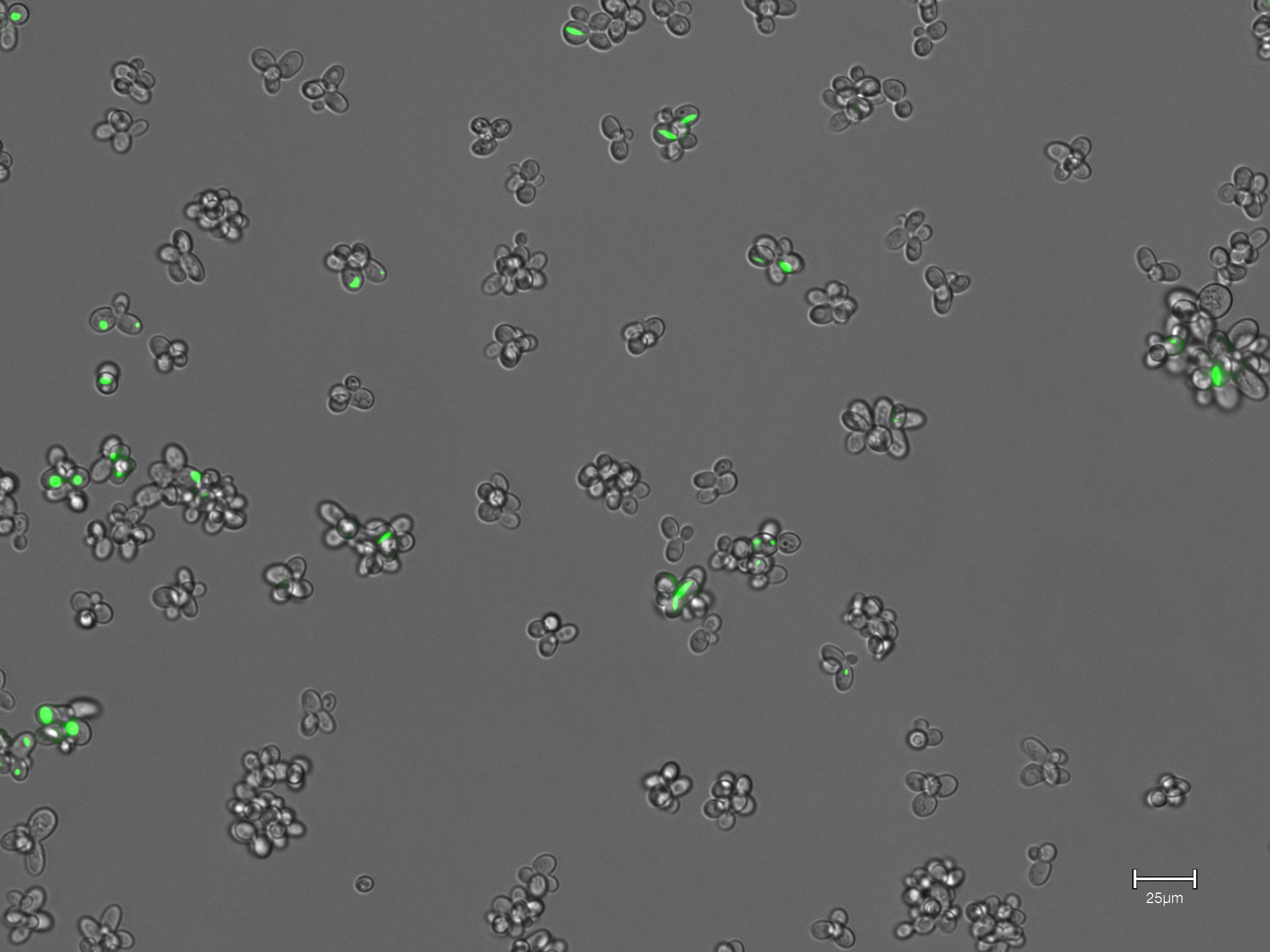

Supplement: Supplementary file 12 — Source data Fig. 4 [file 44320_2025_114_MOESM12_ESM.zip › Figure 4/4B/GFP yeast/Fig-HA10_0024.tif]

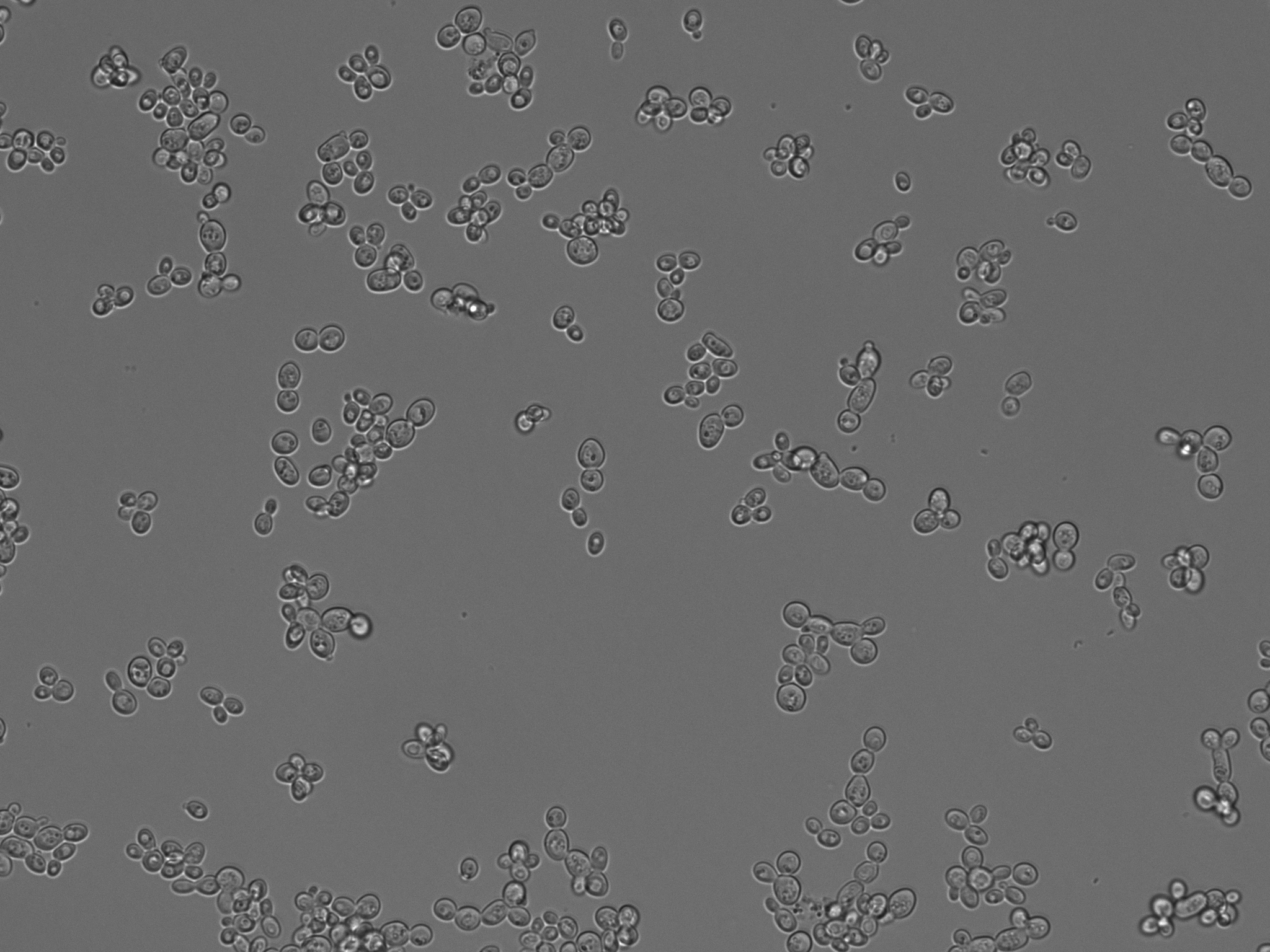

Supplement: Supplementary file 12 — Source data Fig. 4 [file 44320_2025_114_MOESM12_ESM.zip › Figure 4/4B/GFP yeast/Fig-HP1_0003_Trans.tif]

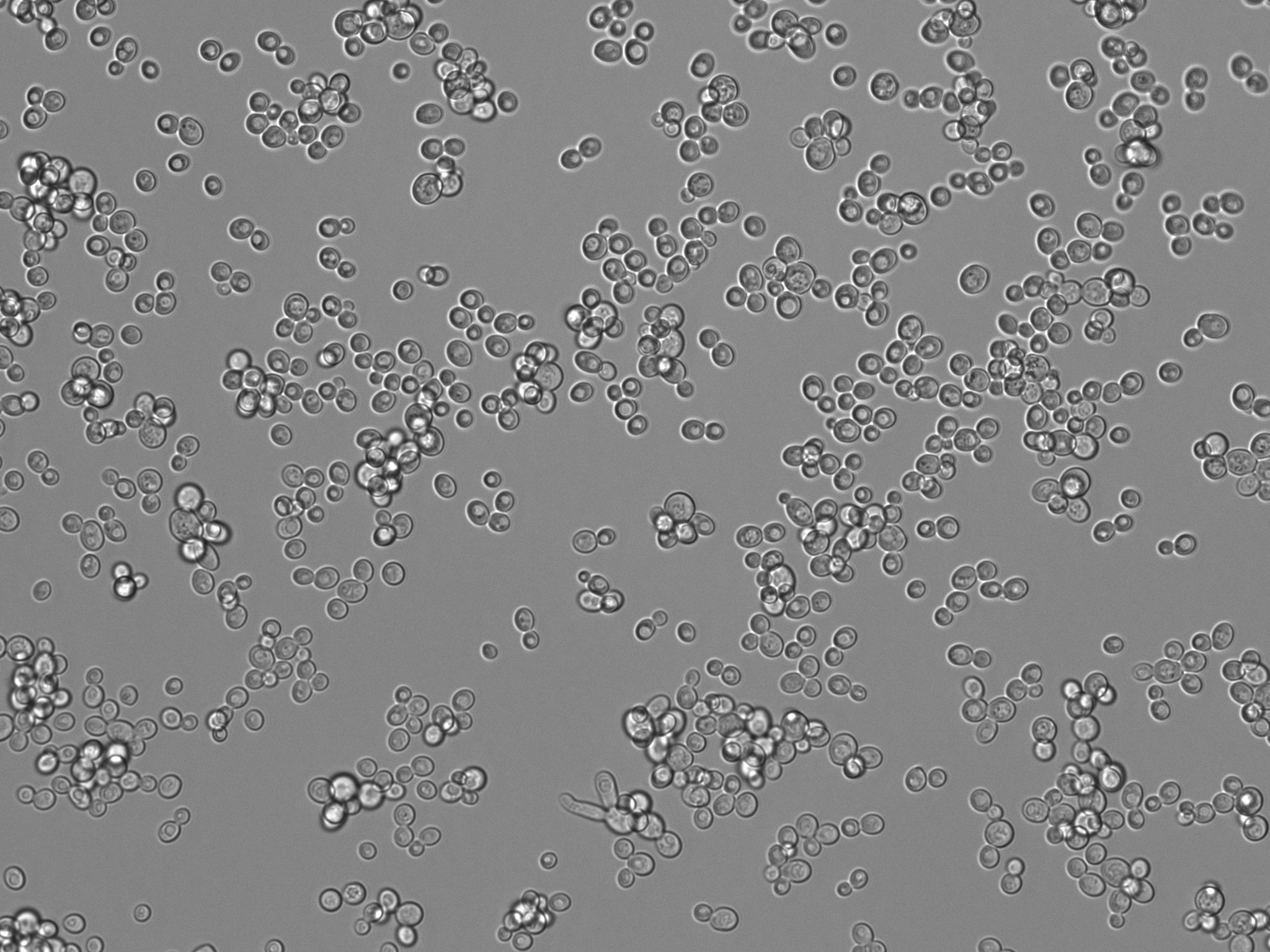

Supplement: Supplementary file 12 — Source data Fig. 4 [file 44320_2025_114_MOESM12_ESM.zip › Figure 4/4B/GFP yeast/Fig-deltaNM_0003_Trans.tif]

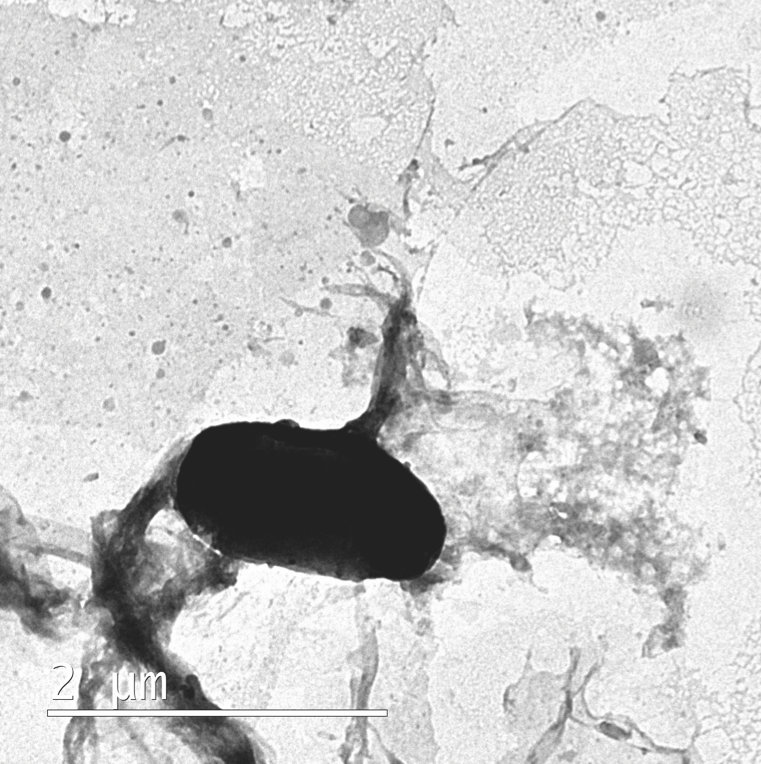

Supplement: Supplementary file 13 — Source data Fig. 5 [file 44320_2025_114_MOESM13_ESM.zip › Figure 5/5C/P9_2500x.tif]

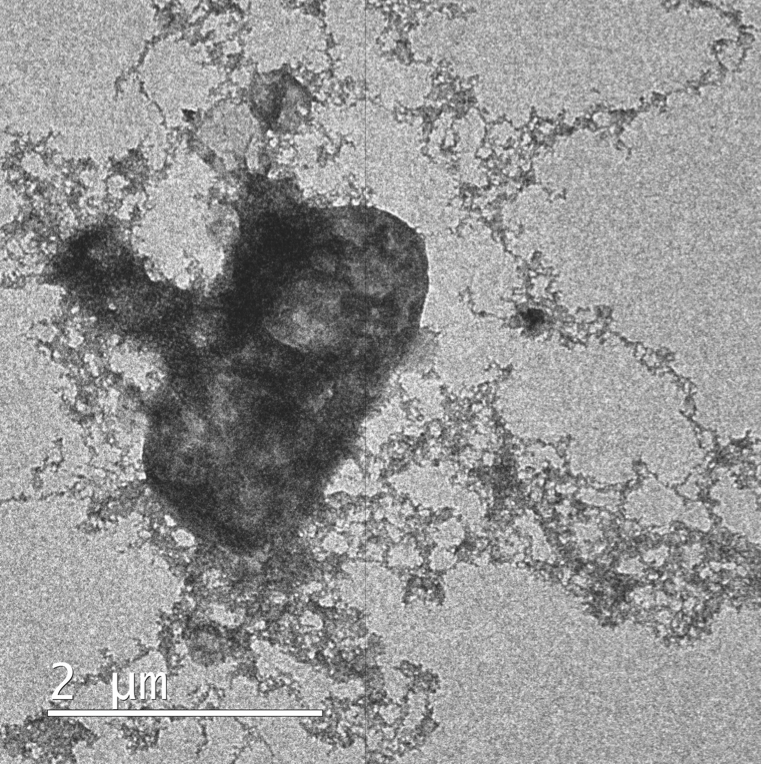

Supplement: Supplementary file 13 — Source data Fig. 5 [file 44320_2025_114_MOESM13_ESM.zip › Figure 5/5C/BH4_2000x.tif]

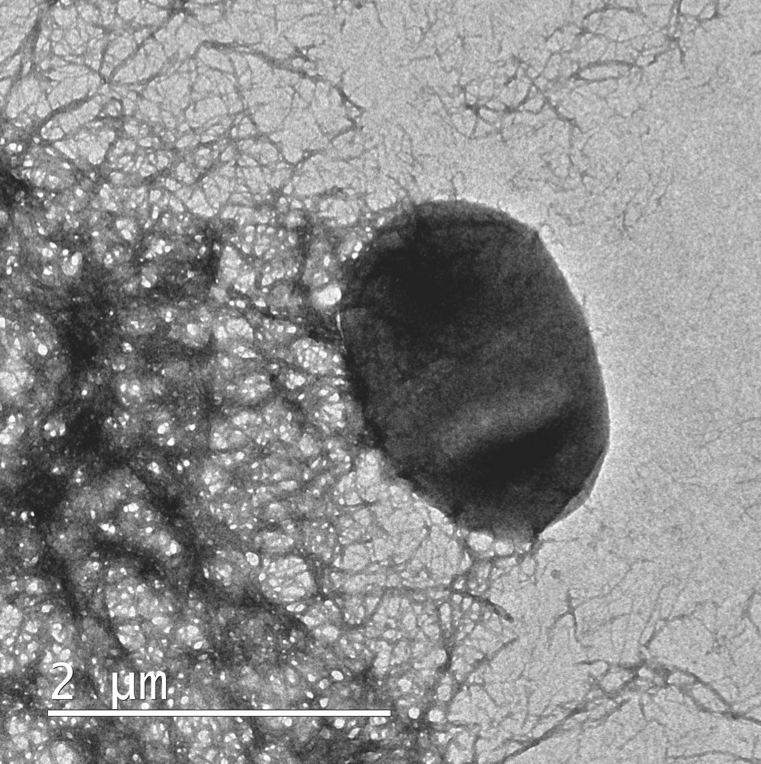

Supplement: Supplementary file 13 — Source data Fig. 5 [file 44320_2025_114_MOESM13_ESM.zip › Figure 5/5C/SA7_2500x.tif]

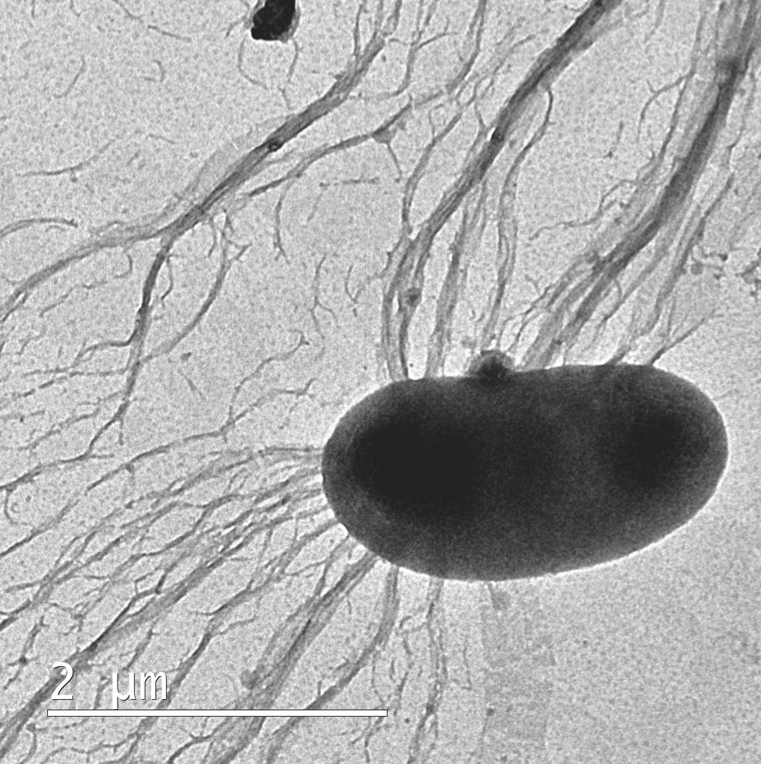

Supplement: Supplementary file 13 — Source data Fig. 5 [file 44320_2025_114_MOESM13_ESM.zip › Figure 5/5C/RI5_2500x.tif]

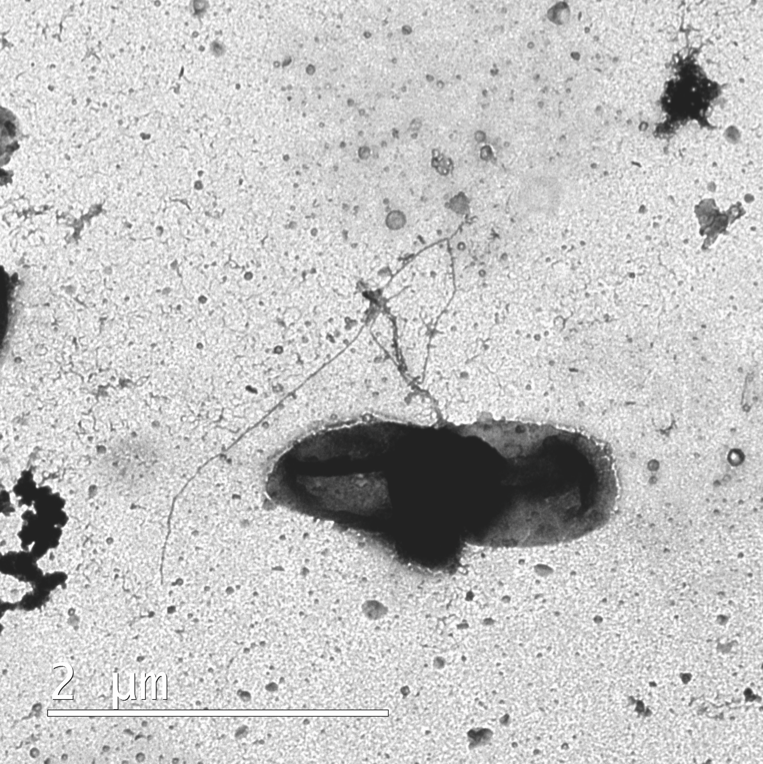

Supplement: Supplementary file 13 — Source data Fig. 5 [file 44320_2025_114_MOESM13_ESM.zip › Figure 5/5C/HP2_2500x.tif]

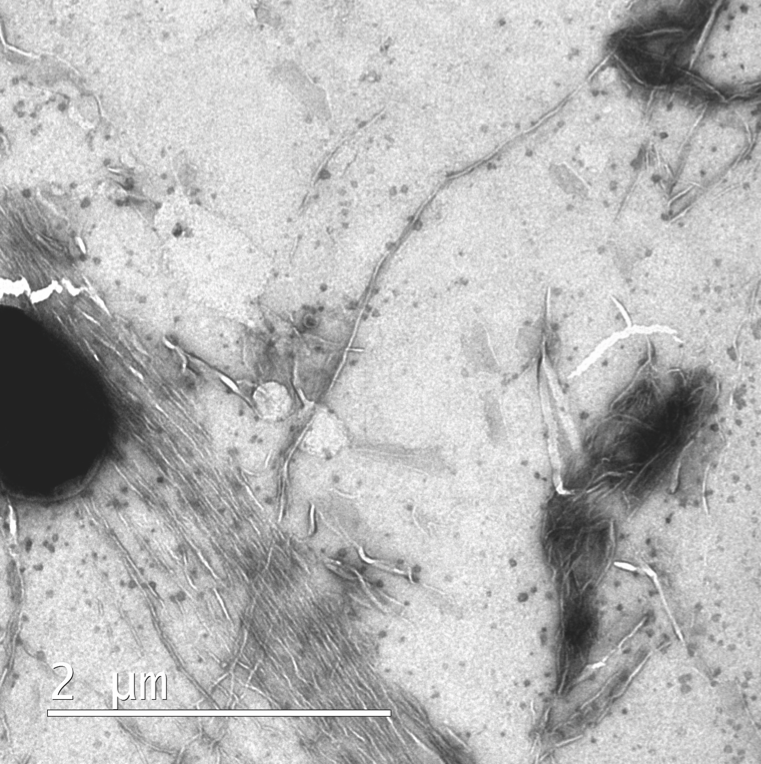

Supplement: Supplementary file 13 — Source data Fig. 5 [file 44320_2025_114_MOESM13_ESM.zip › Figure 5/5C/RI6_2500x.tif]

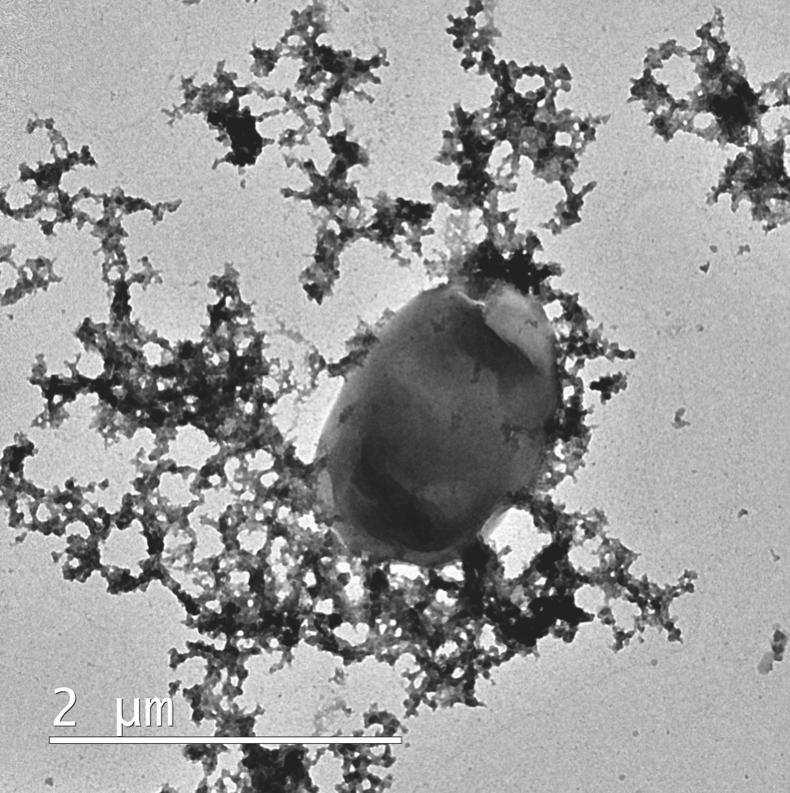

Supplement: Supplementary file 13 — Source data Fig. 5 [file 44320_2025_114_MOESM13_ESM.zip › Figure 5/5C/HP1_2500x.tif]

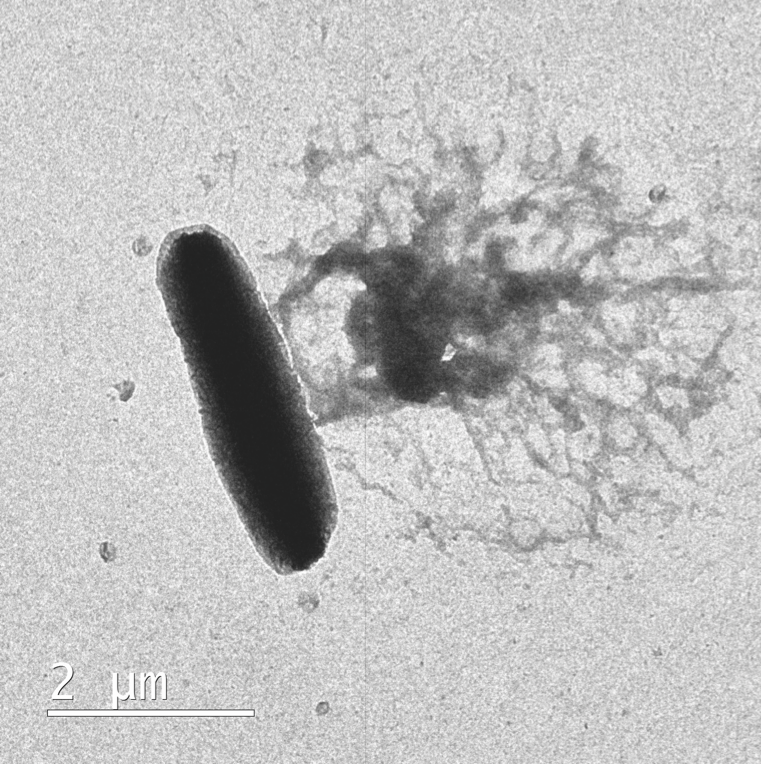

Supplement: Supplementary file 13 — Source data Fig. 5 [file 44320_2025_114_MOESM13_ESM.zip › Figure 5/5C/NM_1500x.tif]

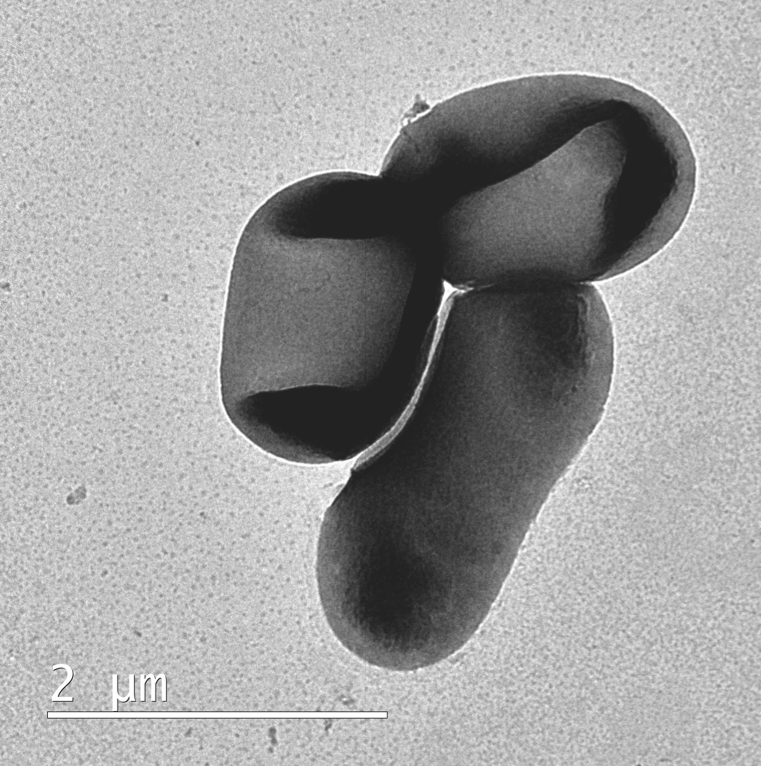

Supplement: Supplementary file 13 — Source data Fig. 5 [file 44320_2025_114_MOESM13_ESM.zip › Figure 5/5C/M_2500x.tif]

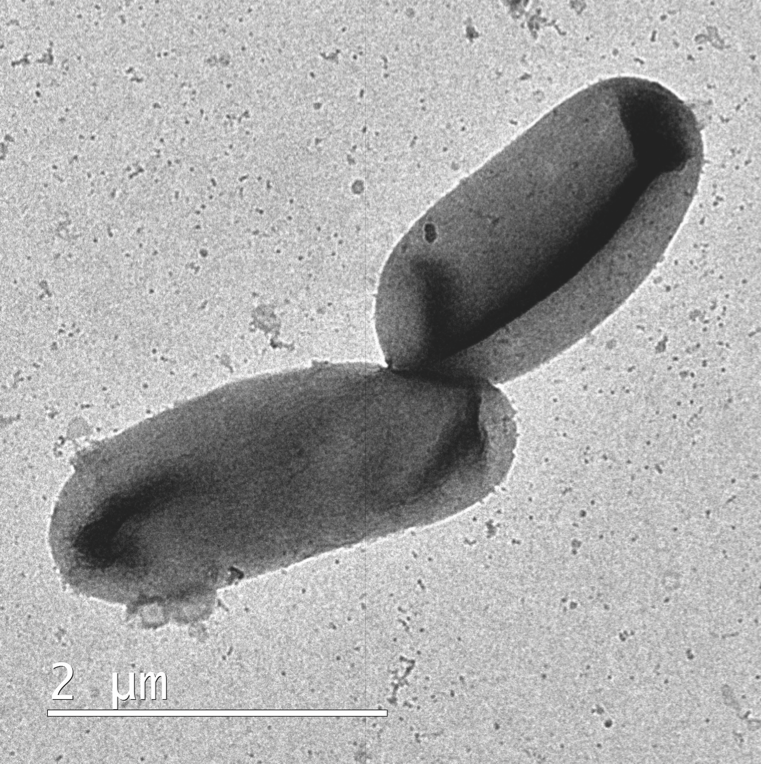

Supplement: Supplementary file 13 — Source data Fig. 5 [file 44320_2025_114_MOESM13_ESM.zip › Figure 5/5C/DeltaNM_2500x.tif]

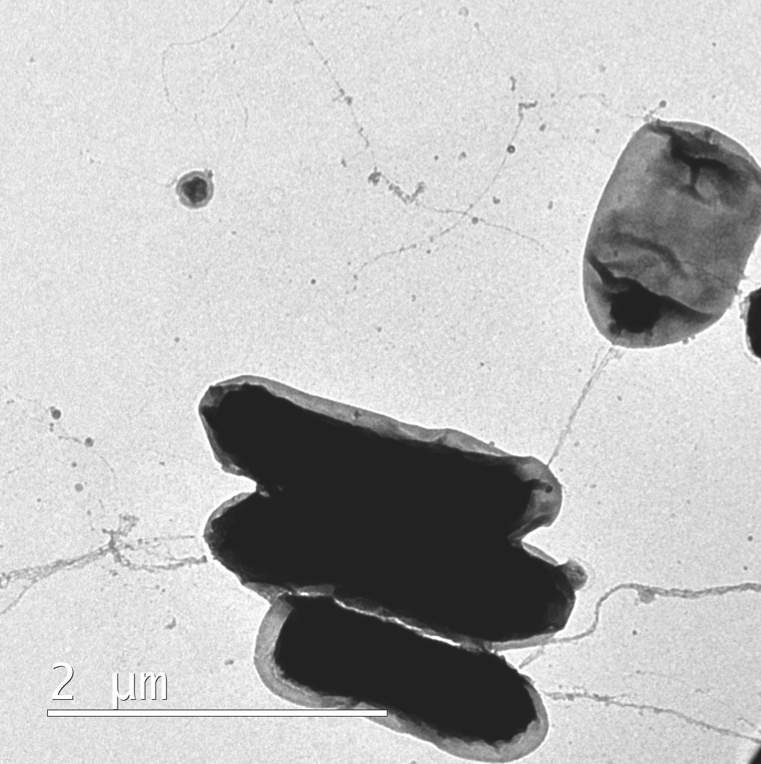

Supplement: Supplementary file 13 — Source data Fig. 5 [file 44320_2025_114_MOESM13_ESM.zip › Figure 5/5C/LD8_2500X.tif]

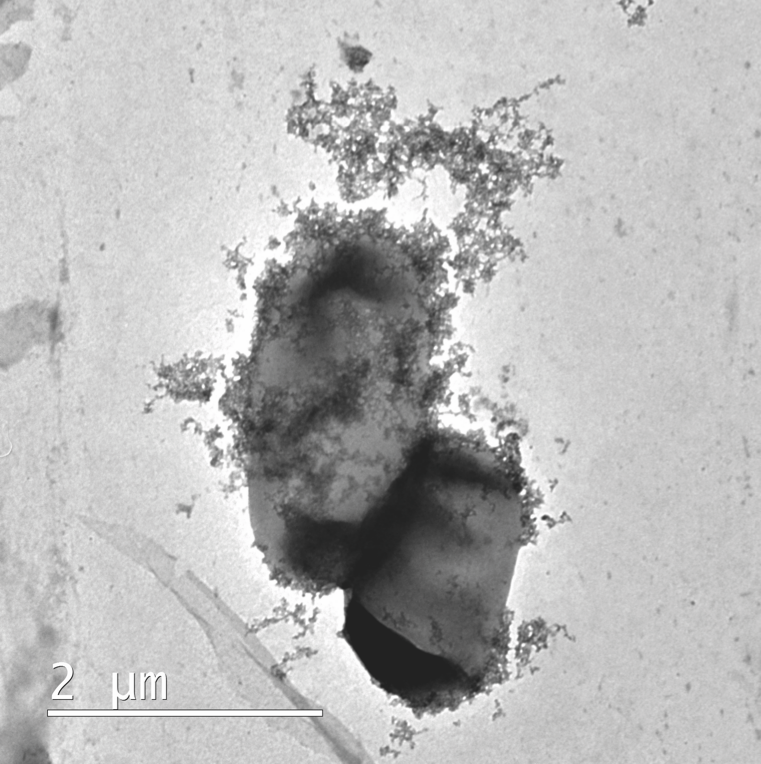

Supplement: Supplementary file 13 — Source data Fig. 5 [file 44320_2025_114_MOESM13_ESM.zip › Figure 5/5C/HA10_2000x.tif]

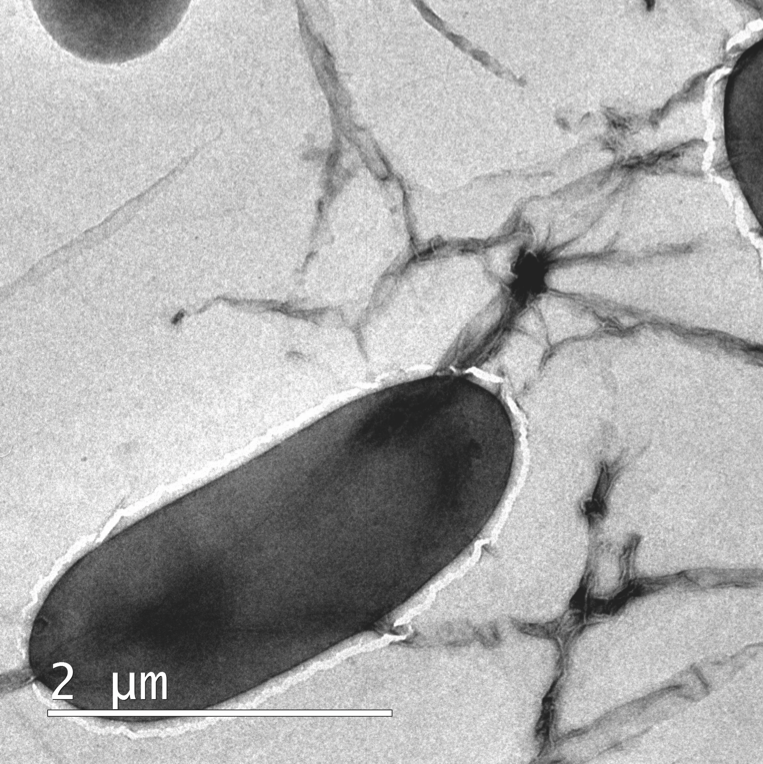

Supplement: Supplementary file 13 — Source data Fig. 5 [file 44320_2025_114_MOESM13_ESM.zip › Figure 5/5C/CC3_2500x.tif]

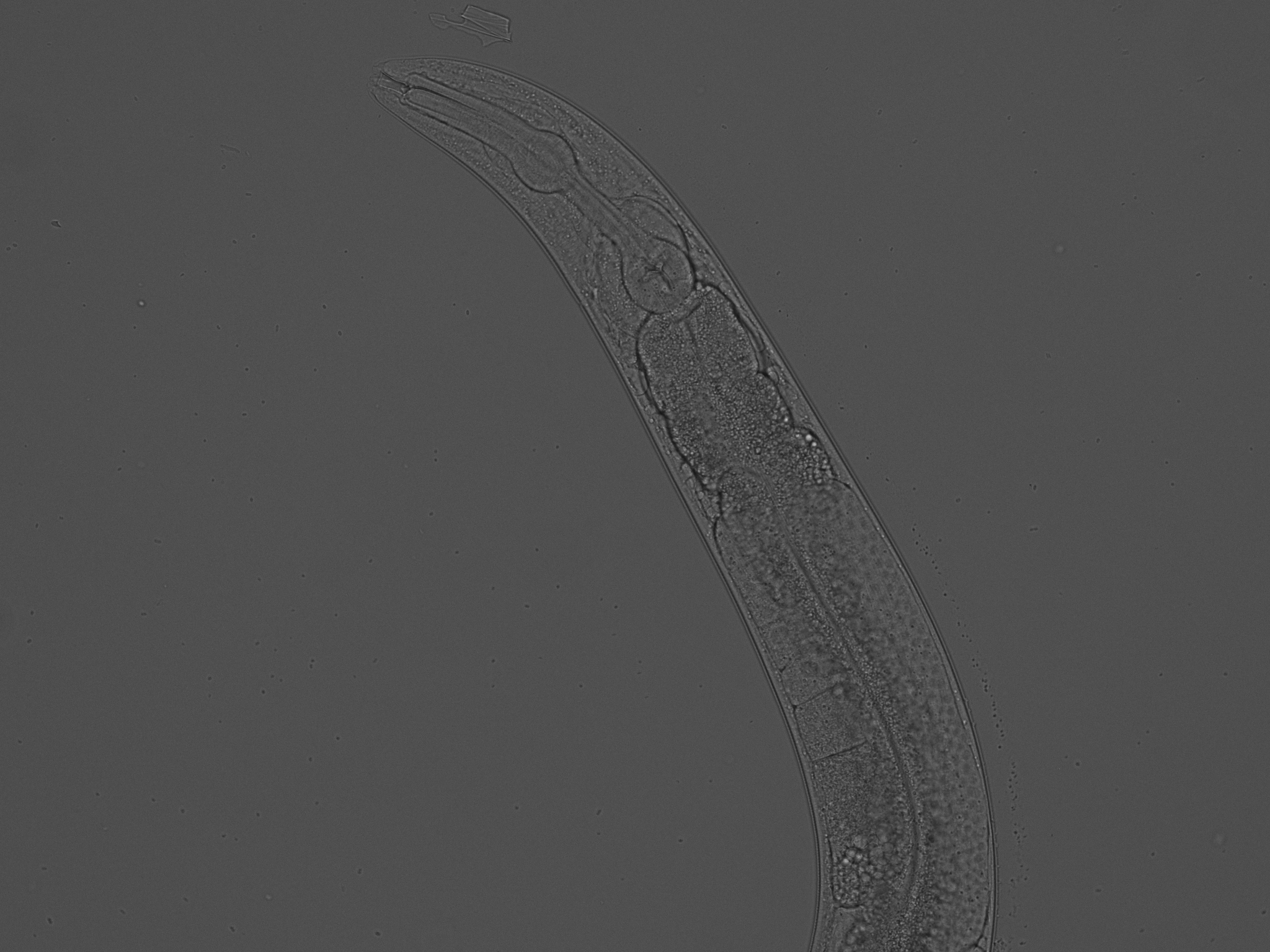

Supplement: Supplementary file 15 — Source data Fig. 7 [file 44320_2025_114_MOESM15_ESM.zip › Figure 7/7A/trans_images/N2_P4_3_0128_Trans.tif]

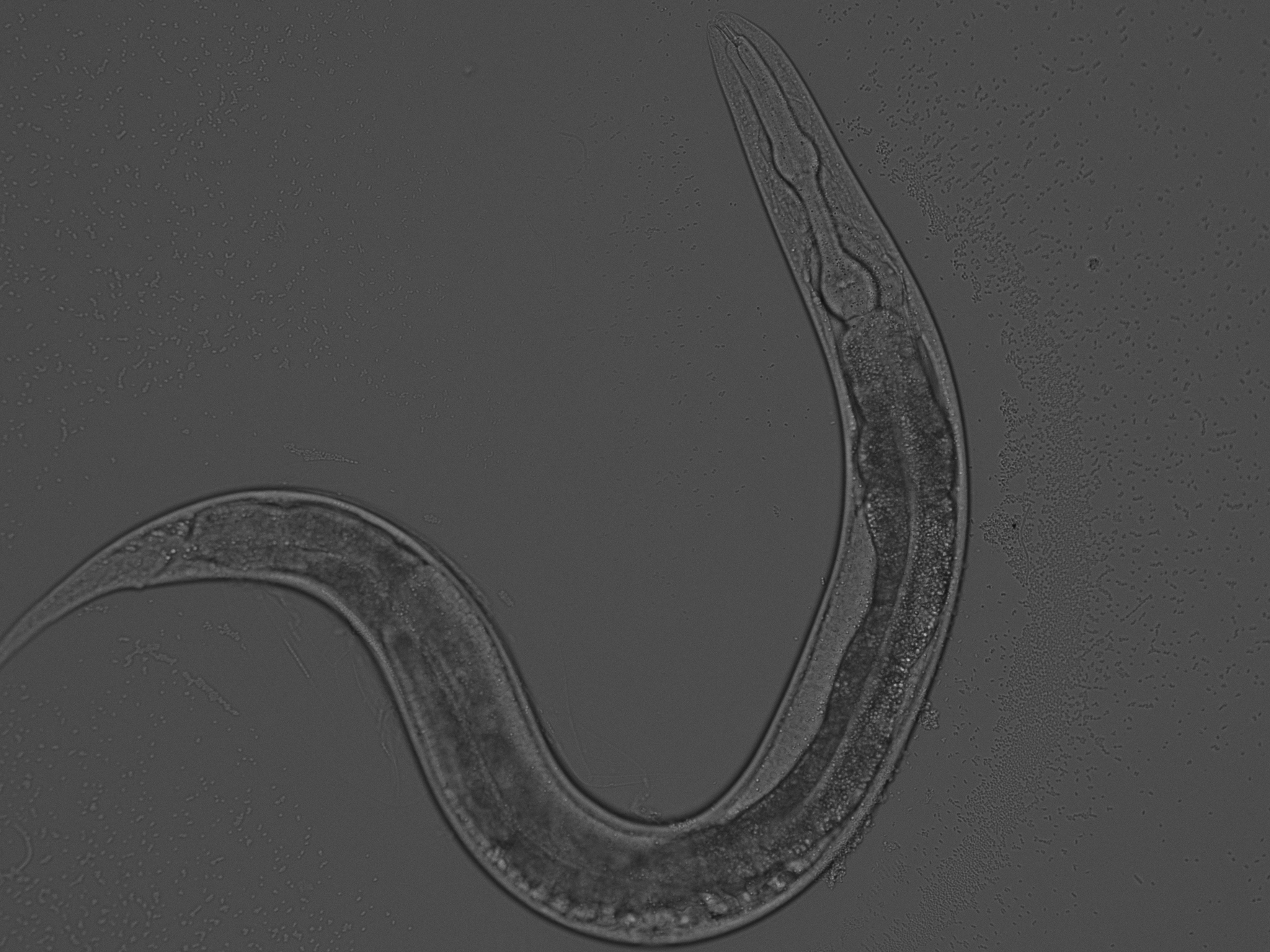

Supplement: Supplementary file 15 — Source data Fig. 7 [file 44320_2025_114_MOESM15_ESM.zip › Figure 7/7A/trans_images/N2_P9_4_0091_Trans.tif]

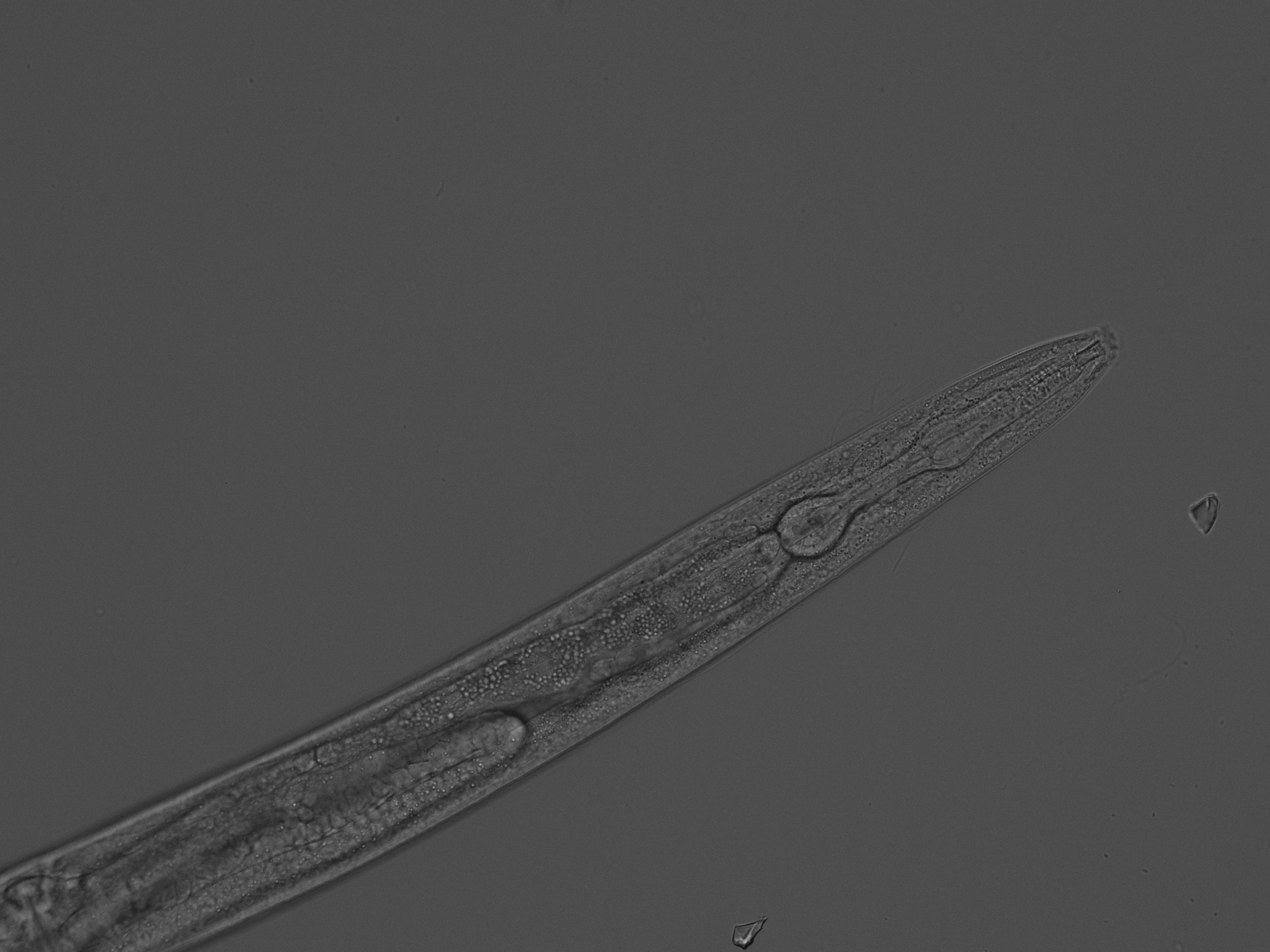

Supplement: Supplementary file 15 — Source data Fig. 7 [file 44320_2025_114_MOESM15_ESM.zip › Figure 7/7A/trans_images/N2_P5_9_0039_Trans.tif]

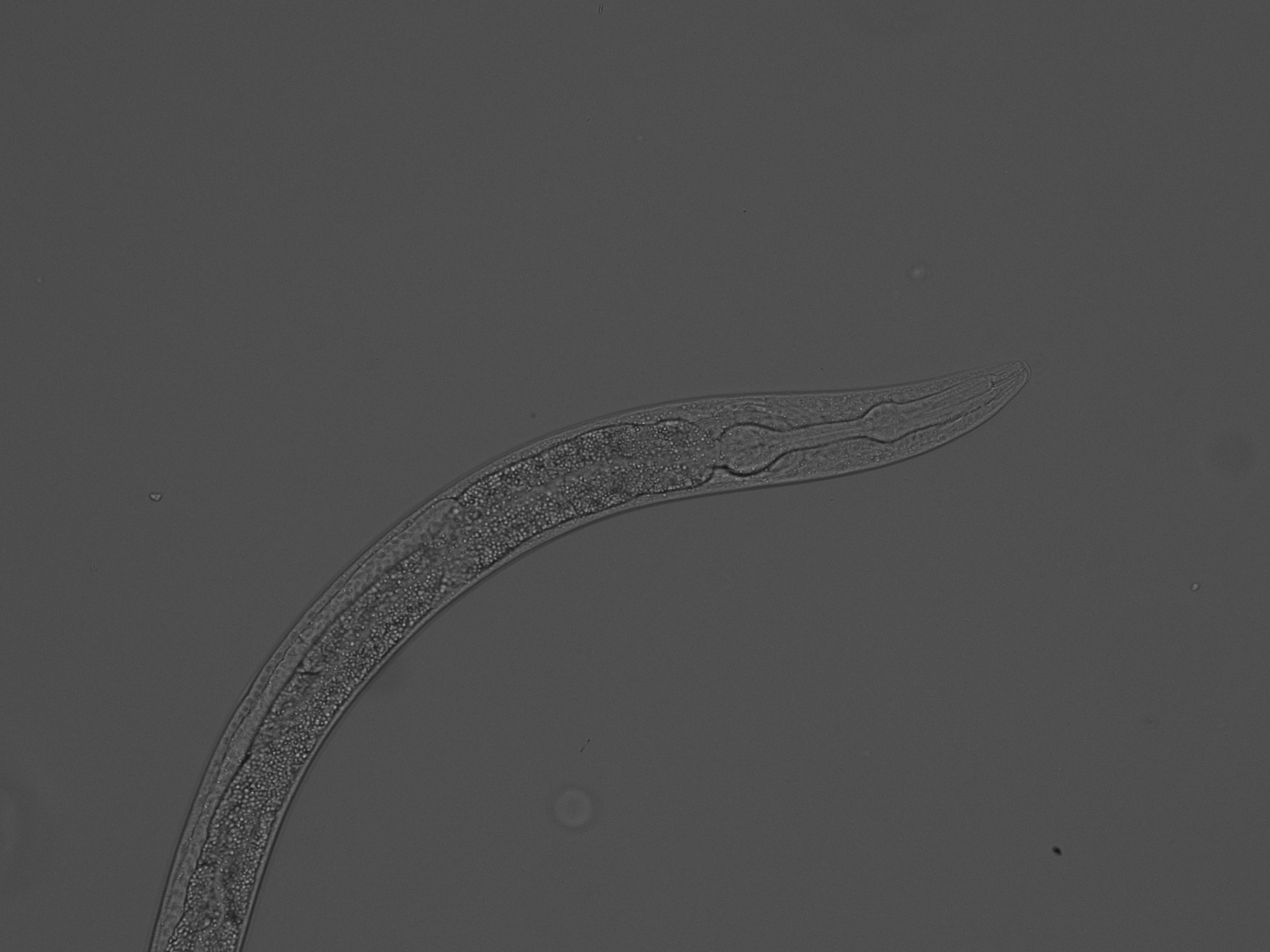

Supplement: Supplementary file 15 — Source data Fig. 7 [file 44320_2025_114_MOESM15_ESM.zip › Figure 7/7A/trans_images/N2_op50_2_0109_Trans.tif]

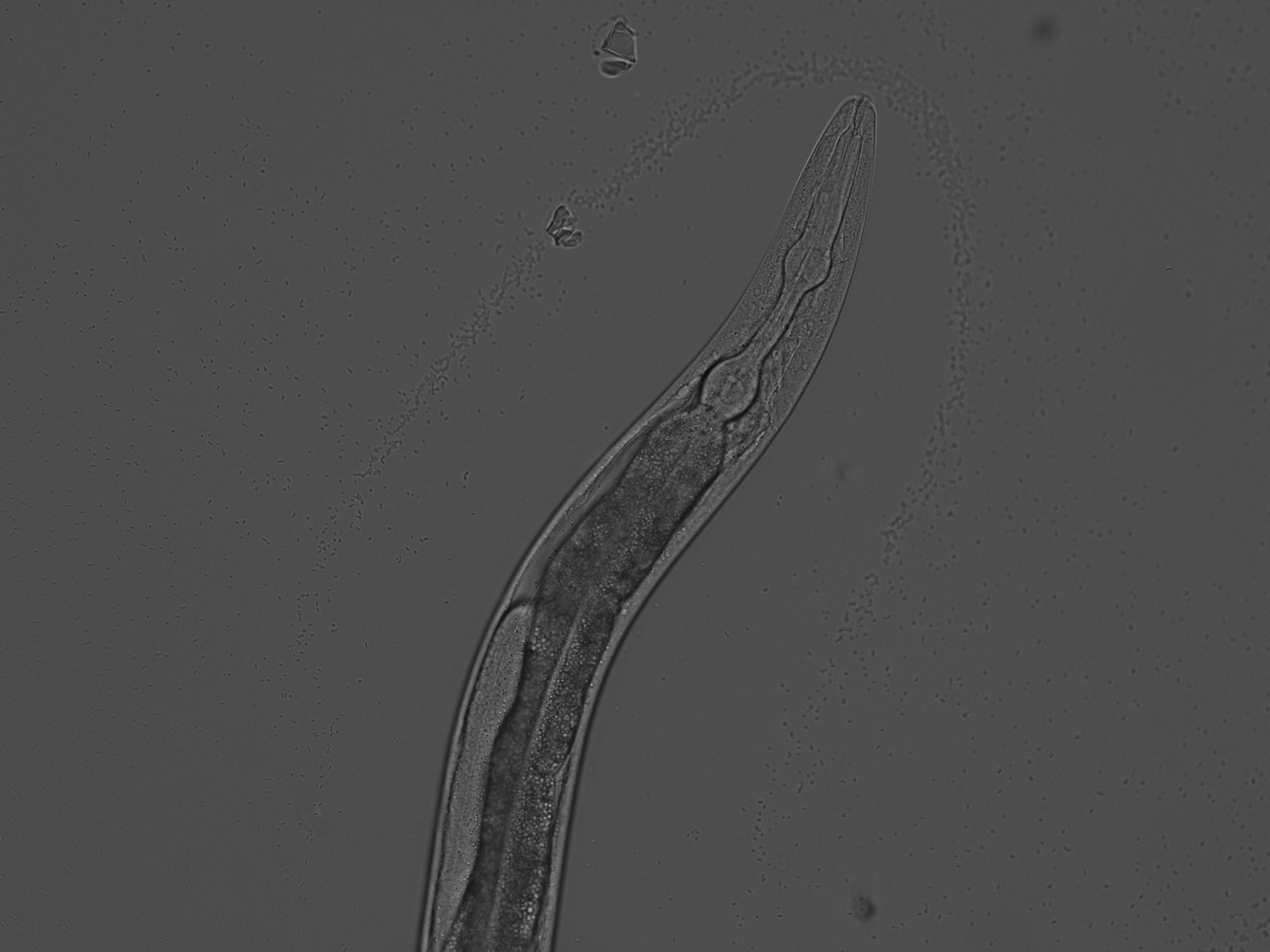

Supplement: Supplementary file 15 — Source data Fig. 7 [file 44320_2025_114_MOESM15_ESM.zip › Figure 7/7A/trans_images/N2_A1-40_8_0007_Trans.tif]

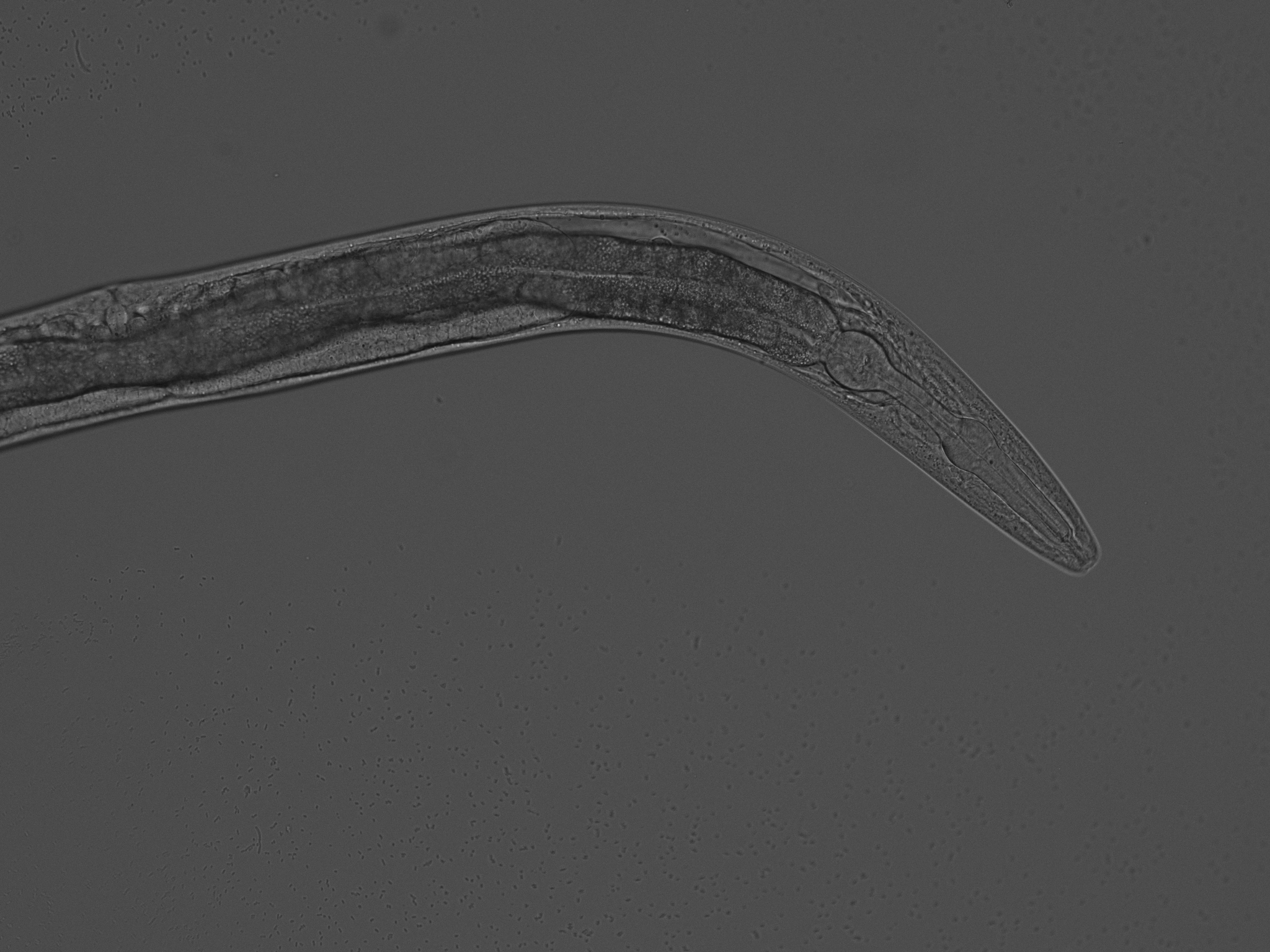

Supplement: Supplementary file 15 — Source data Fig. 7 [file 44320_2025_114_MOESM15_ESM.zip › Figure 7/7A/trans_images/N2_P6_10_0029_Trans.tif]

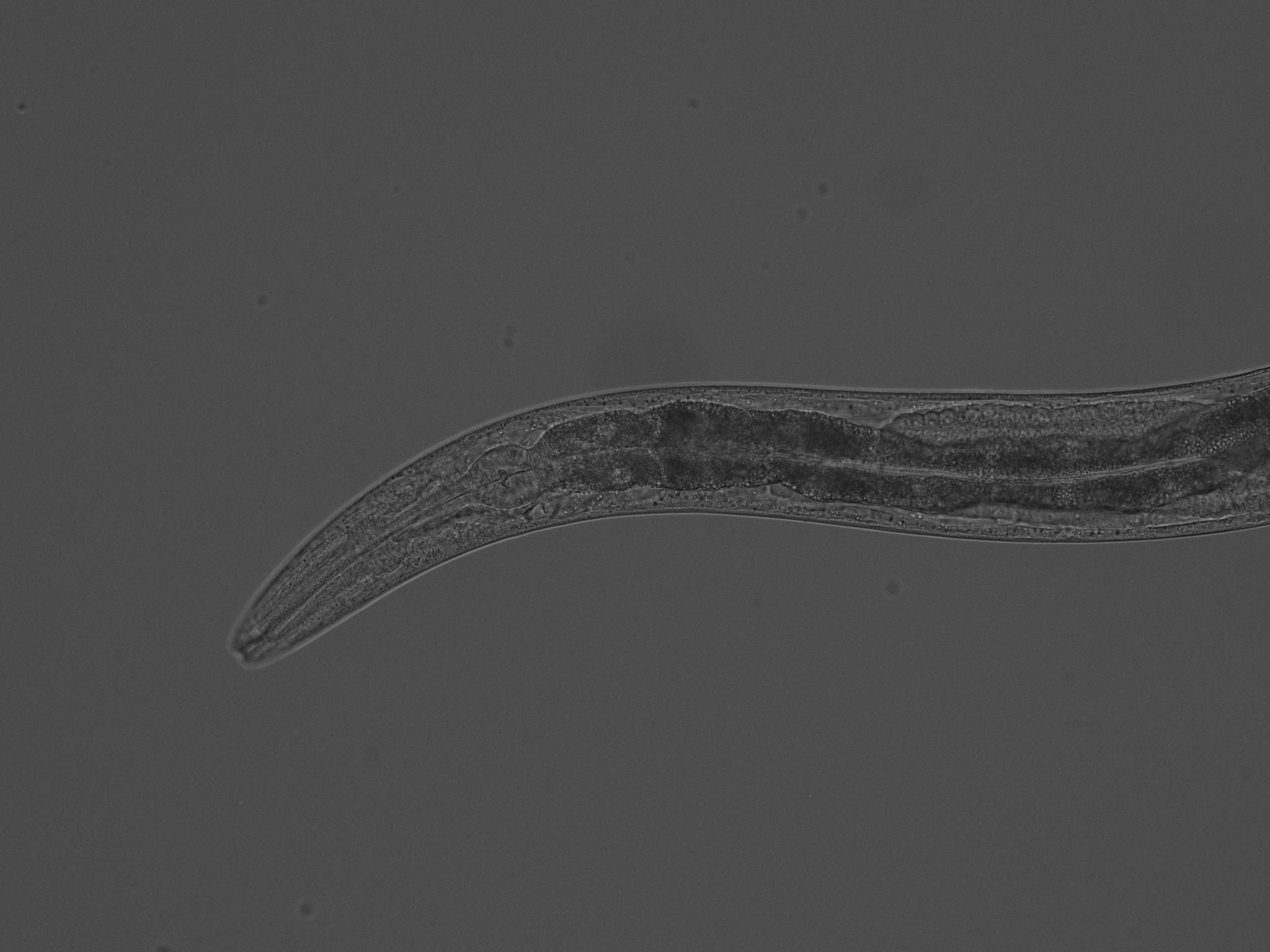

Supplement: Supplementary file 15 — Source data Fig. 7 [file 44320_2025_114_MOESM15_ESM.zip › Figure 7/7A/trans_images/N2_P8_5_0054_Trans.tif]

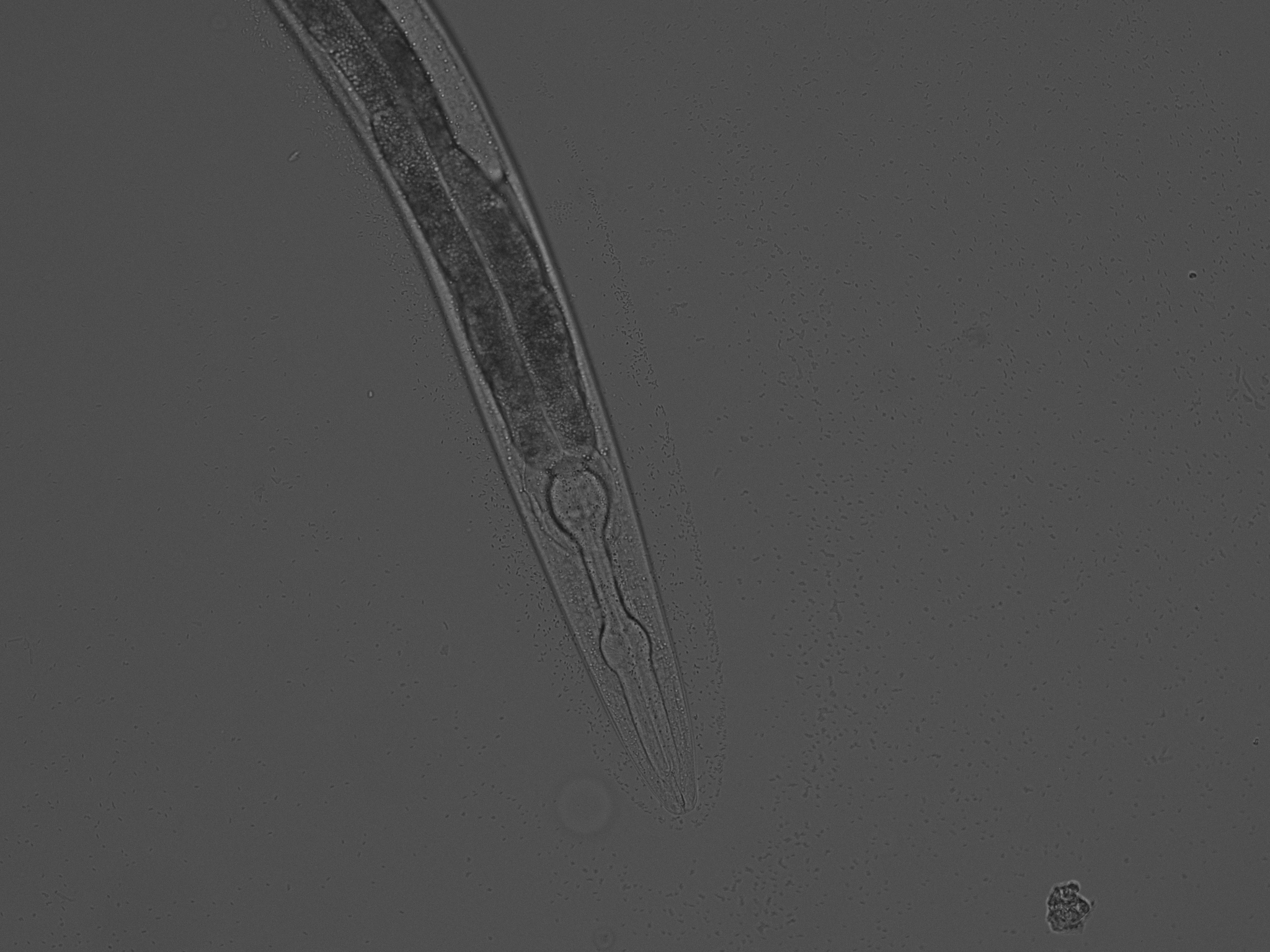

Supplement: Supplementary file 15 — Source data Fig. 7 [file 44320_2025_114_MOESM15_ESM.zip › Figure 7/7A/trans_images/N2_P11_4_0120_Trans.tif]

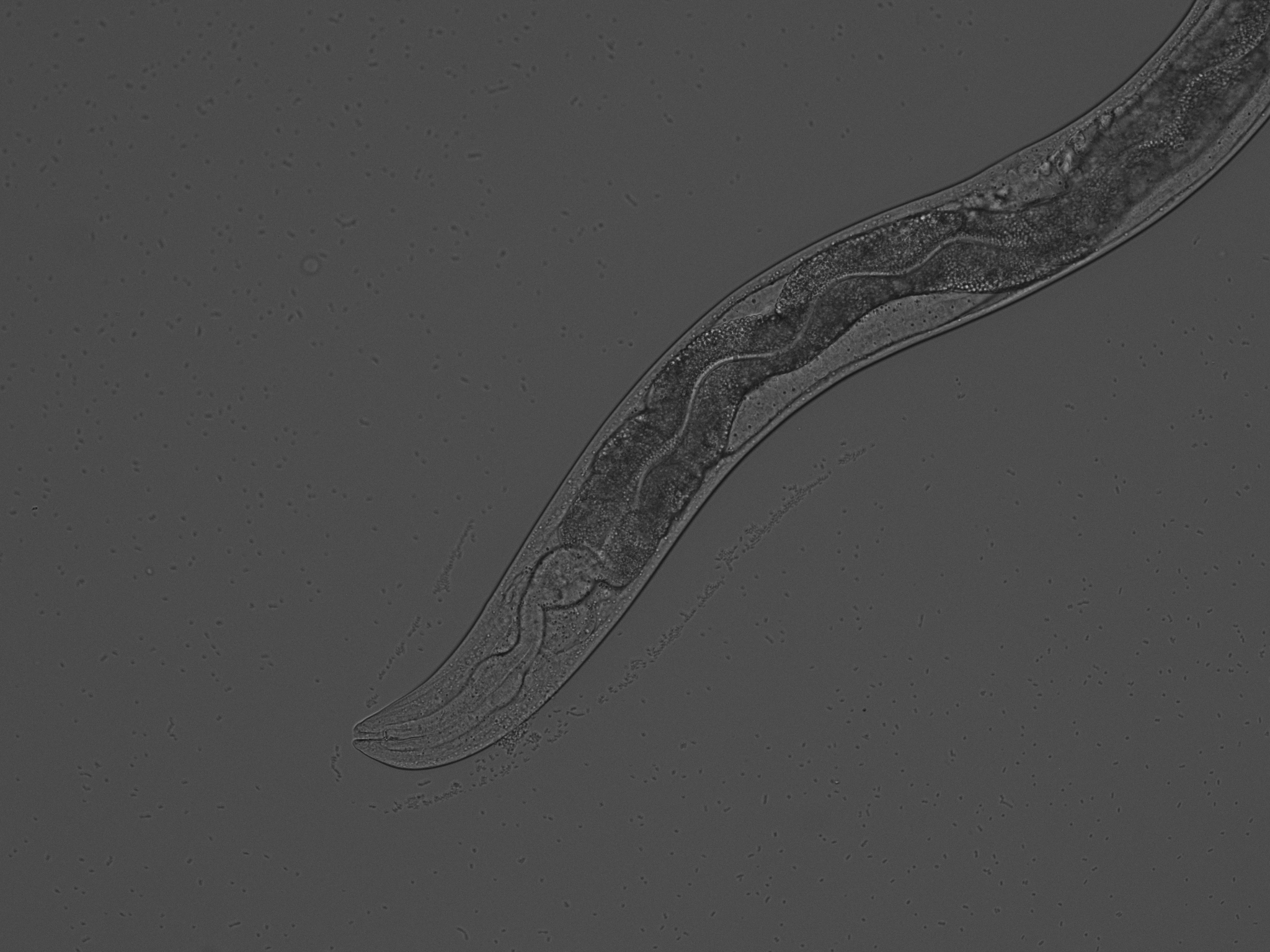

Supplement: Supplementary file 15 — Source data Fig. 7 [file 44320_2025_114_MOESM15_ESM.zip › Figure 7/7A/trans_images/N2_P10_2_0098_Trans.tif]

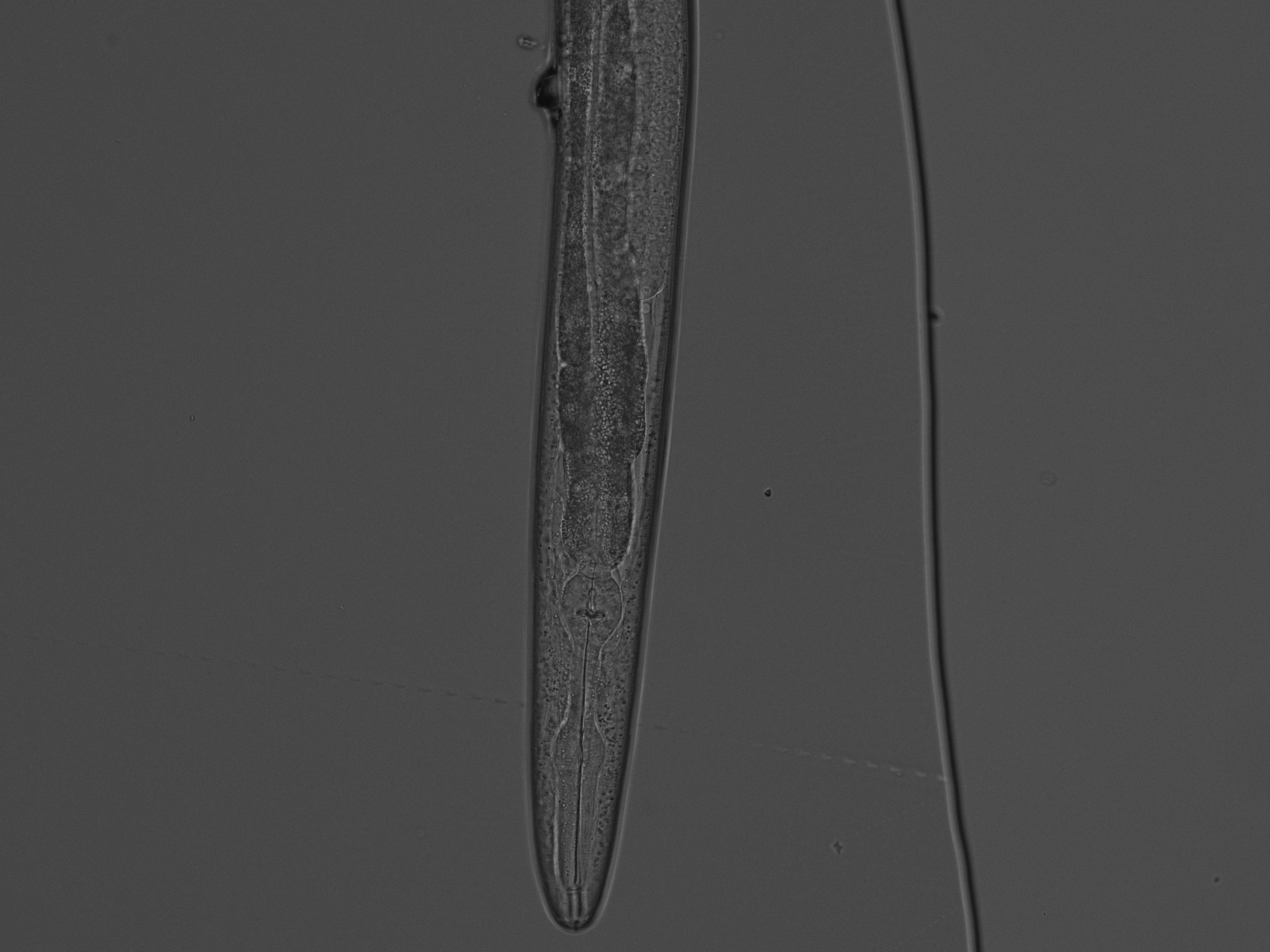

Supplement: Supplementary file 15 — Source data Fig. 7 [file 44320_2025_114_MOESM15_ESM.zip › Figure 7/7A/trans_images/N2_P2_1_0070_Trans.tif]

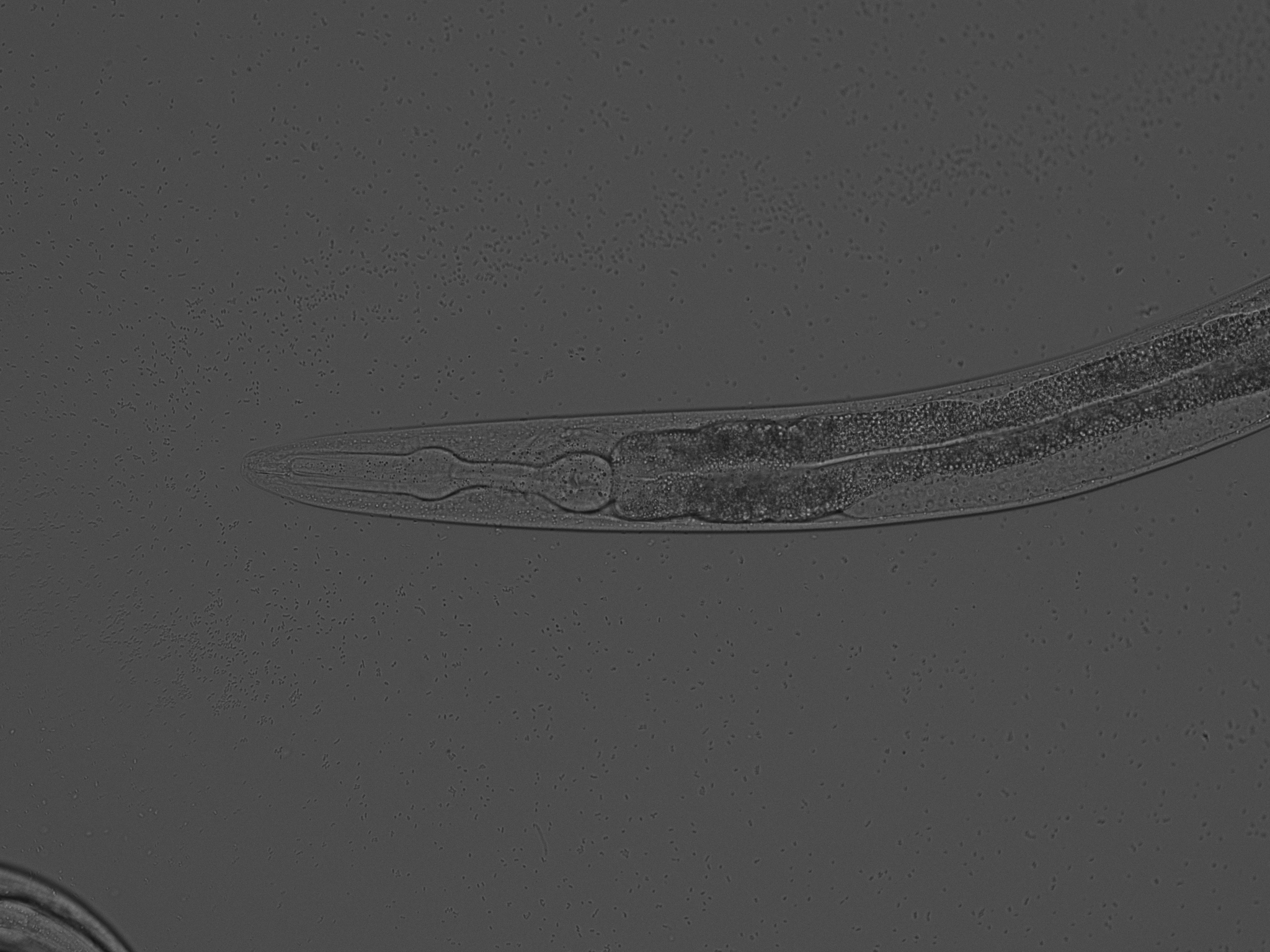

Supplement: Supplementary file 15 — Source data Fig. 7 [file 44320_2025_114_MOESM15_ESM.zip › Figure 7/7A/trans_images/N2_P3_3_0064_Trans.tif]

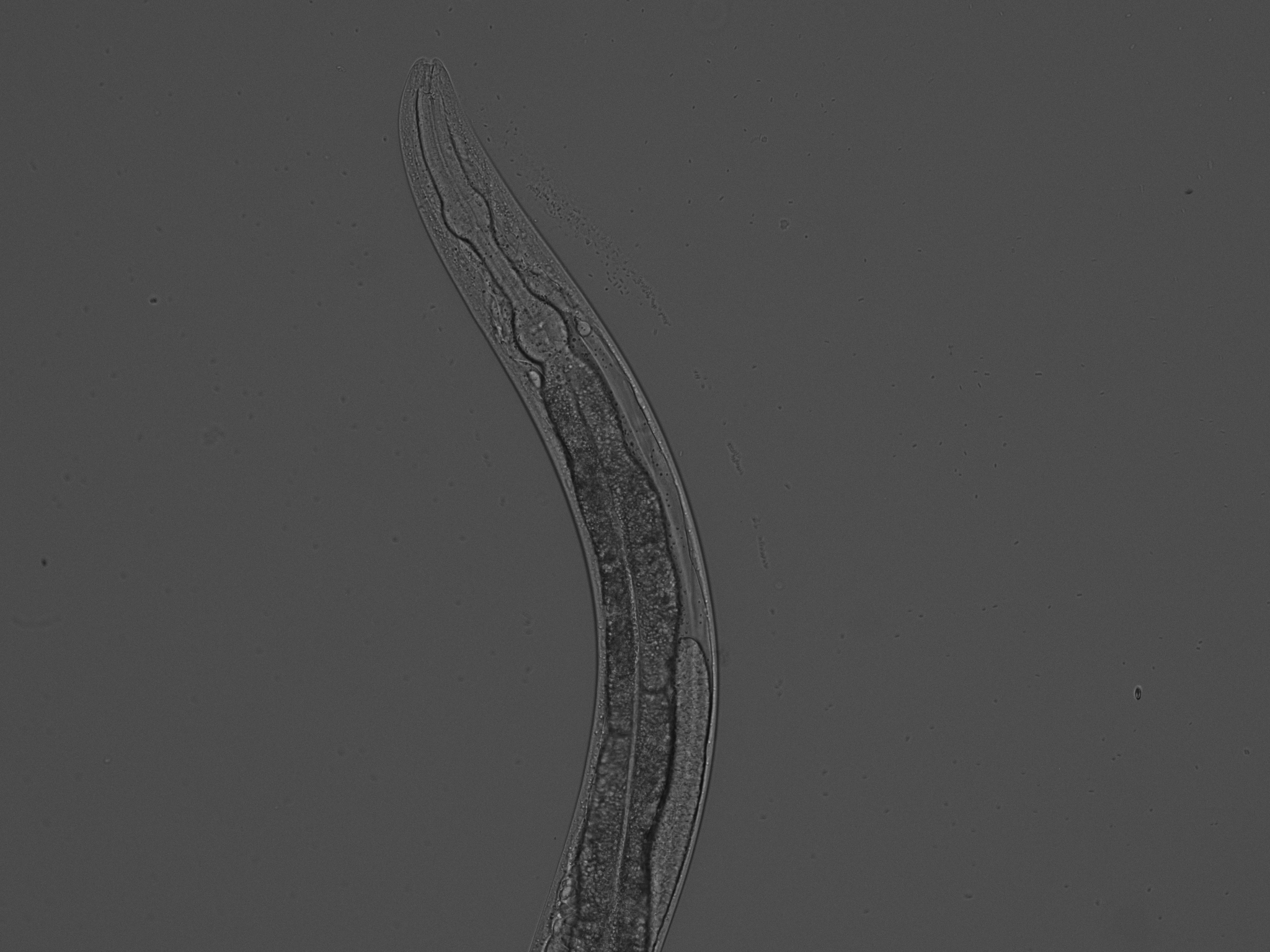

Supplement: Supplementary file 15 — Source data Fig. 7 [file 44320_2025_114_MOESM15_ESM.zip › Figure 7/7A/trans_images/N2_P7_2_0042_Trans.tif]

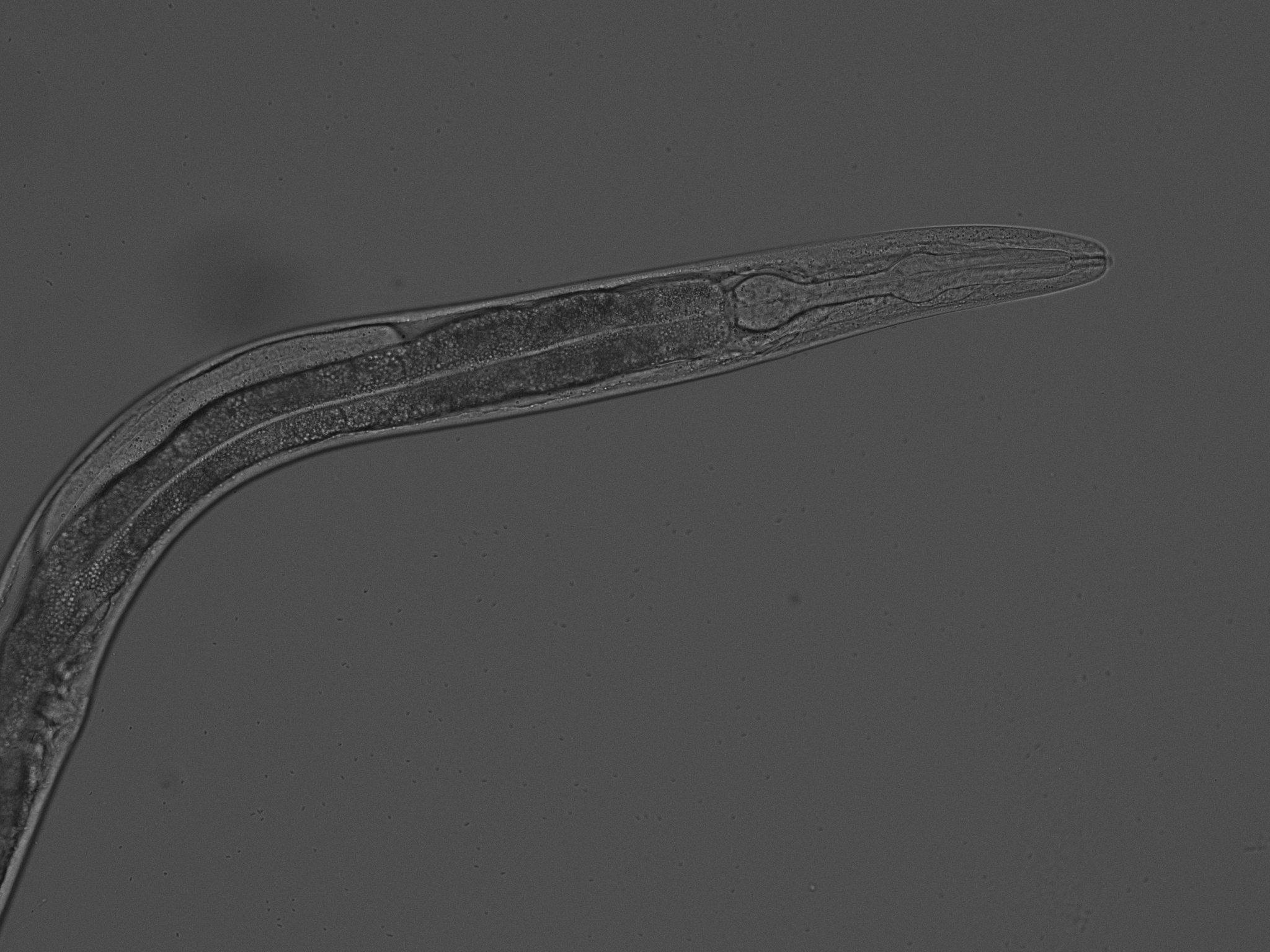

Supplement: Supplementary file 15 — Source data Fig. 7 [file 44320_2025_114_MOESM15_ESM.zip › Figure 7/7A/trans_images/N2_PVS72_5_0012_Trans.tif]
