# Supplementary material for: Exogenous prion-like proteins and their potential to trigger cognitive dysfunction
Source: Mol Syst Biol. 2025 May 27;21(8):1004–29. doi: 10.1038/s44320-025-00114-4 (PMC12322145; doi:10.1038/s44320-025-00114-4)
Supplement: Supplementary file 16 — Expanded View Figures [file 44320_2025_114_MOESM16_ESM.pdf]

# Expanded View Figures

**Figure EV1. Sequential information regarding the ten prion-like sequences selected from bacteria found in the gut.**

Diagram showing the prion-like regions predicted for all the amyloid cores selected. In green are the main sequences. In light blue are the prion domains (PrD) predicted by PLAAC. In dark blue are the prion domains predicted by PAPA. In purple, the amyloid cores predicted by pWALTZ. Turquoise and yellow are other domains.

© The Author(s)

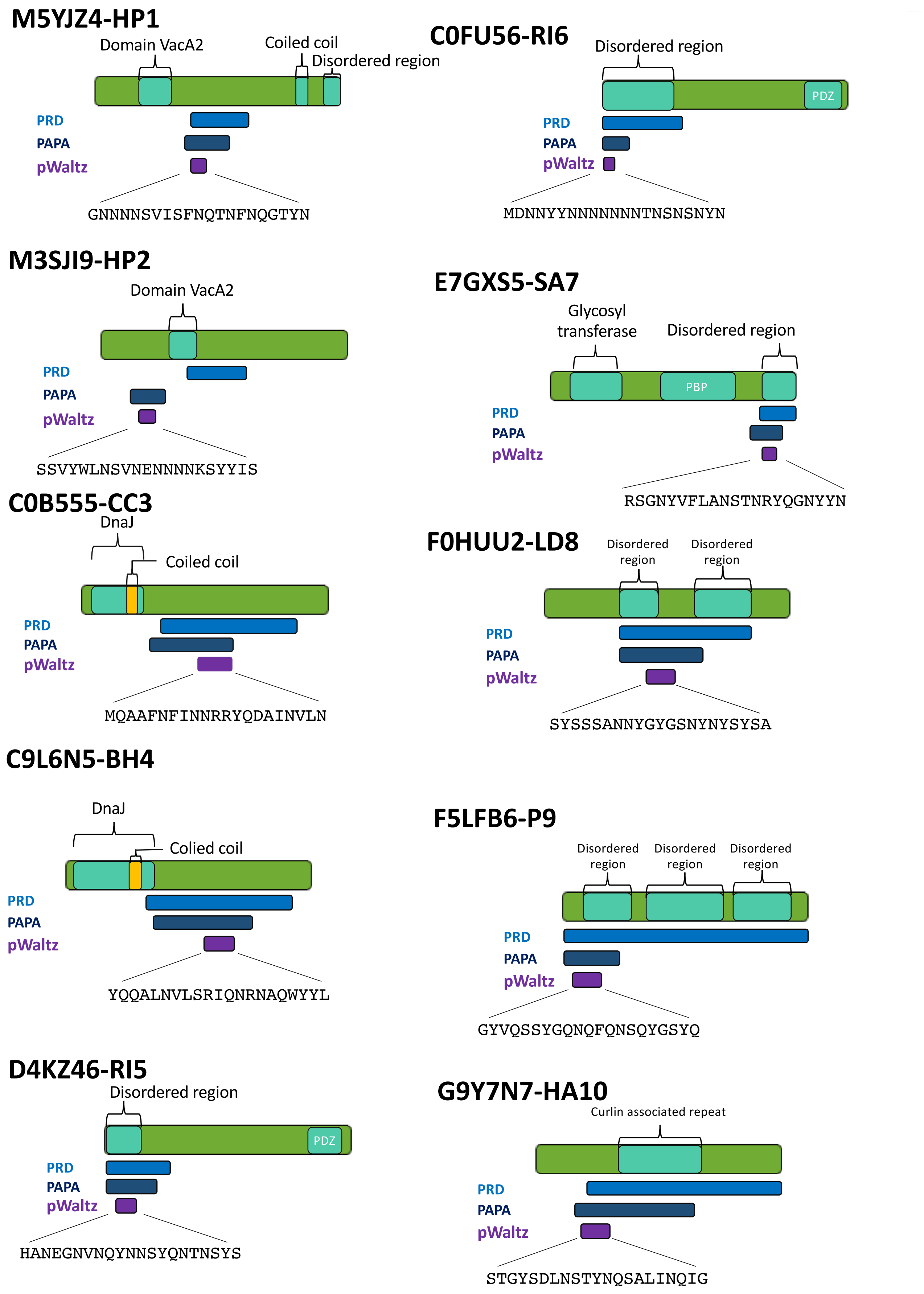
M5YJZ4-HP1
Domain VacA2
Coiled coil
Disordered region
PRD
PAPA
pWaltz
GNNNNSVISFNQTNFNQGTYN
C0FU56-RI6
Disordered region
PDZ
PRD
PAPA
pWaltz
MDNNYYNNNNNNNTNSNSNYN
M3SJI9-HP2
Domain VacA2
PRD
PAPA
pWaltz
SSVYWLNSVNENNNNKSYYIS
E7GXS5-SA7
Glycosyl transferase
Disordered region
PBP
PRD
PAPA
pWaltz
RSGNYVFLANSTNRYQGNYYN
C0B555-CC3
DnaJ
Coiled coil
PRD
PAPA
pWaltz
MQAAFNFINNRRYQDAINVLN
F0HUU2-LD8
Disordered region
Disordered region
PRD
PAPA
pWaltz
SYSSSANNYGYGSNYNYSYSA
C9L6N5-BH4
DnaJ
Colied coil
PRD
PAPA
pWaltz
YQQALNVLSRIQNRNAQWYYL
F5LFB6-P9
Disordered region
Disordered region
Disordered region
PRD
PAPA
pWaltz
GYVQSSYGQNQFQNSQYGSYQ
D4KZ46-RI5
Disordered region
PDZ
PRD
PAPA
pWaltz
HANEGNVNQYNNSYQNTNSYS
G9Y7N7-HA10
Curlin associated repeat
PRD
PAPA
pWaltz
STGYSDLNSTYNQSALINQIG


© The Author(s)

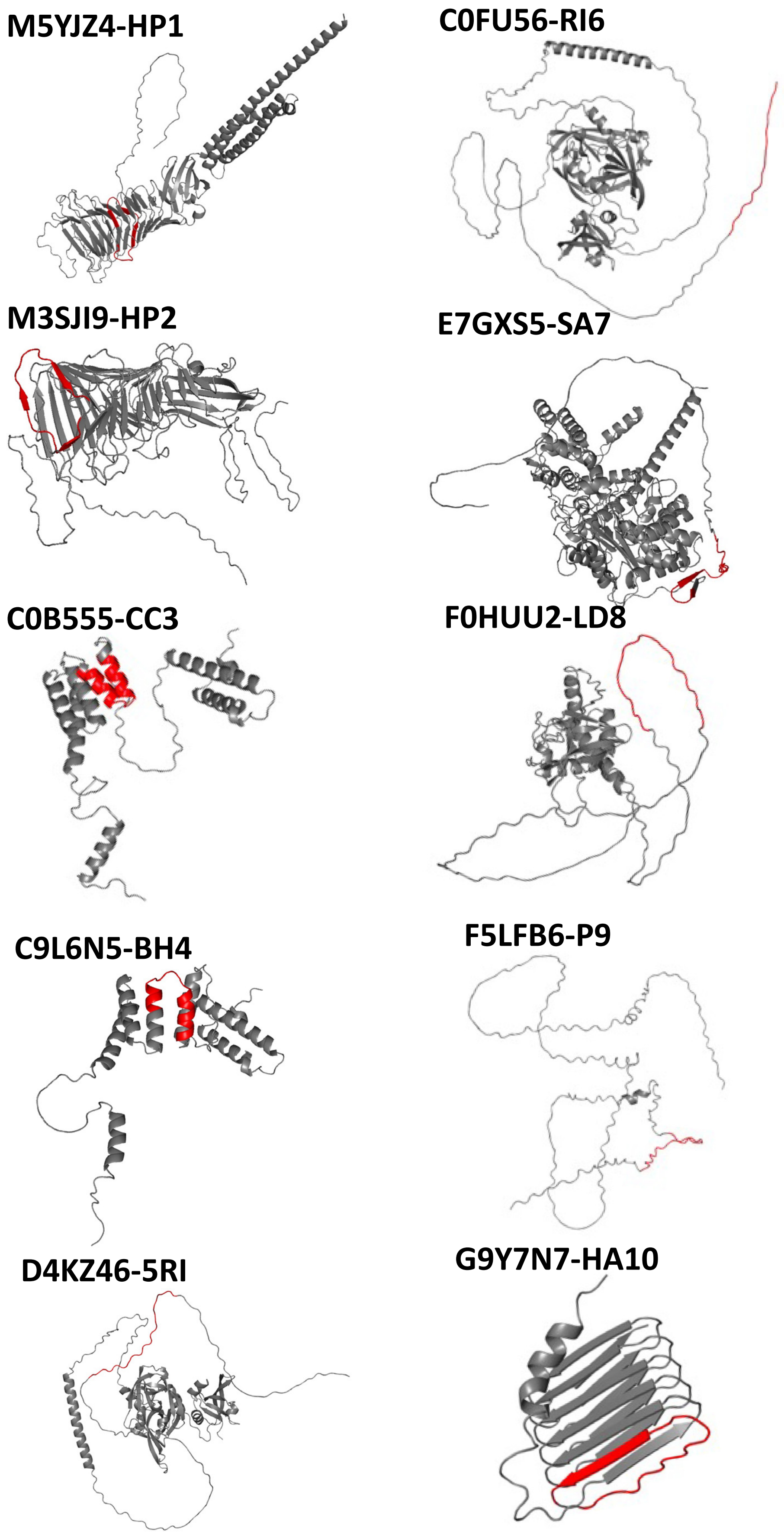
M5YJZ4-HP1
C0FU56-RI6
M3SJI9-HP2
E7GXS5-SA7
C0B555-CC3
F0HUU2-LD8
C9L6N5-BH4
F5LFB6-P9
D4KZ46-5RI
G9Y7N7-HA10

**Figure EV2. Predicted structure of the ten prion-like sequences selected from bacteria found in the gut.**

The images show the Alpha-fold prediction of the whole protein structure.


© The Author(s)
